# Mapping Protein–Protein Interactions at Birth: Single-Particle Cryo-EM Analysis of a Ribosome–Nascent Globin Complex

**DOI:** 10.1021/acscentsci.3c00777

**Published:** 2024-02-01

**Authors:** Meranda M. Masse, Rachel B. Hutchinson, Christopher E. Morgan, Heather J. Allaman, Hongqing Guan, Edward W. Yu, Silvia Cavagnero

**Affiliations:** † Department of Chemistry, University of Wisconsin−Madison, Madison, Wisconsin 53706, United States; ‡ Department of Pharmacology, Case Western Reserve University, Cleveland, Ohio 44106, United States

## Abstract

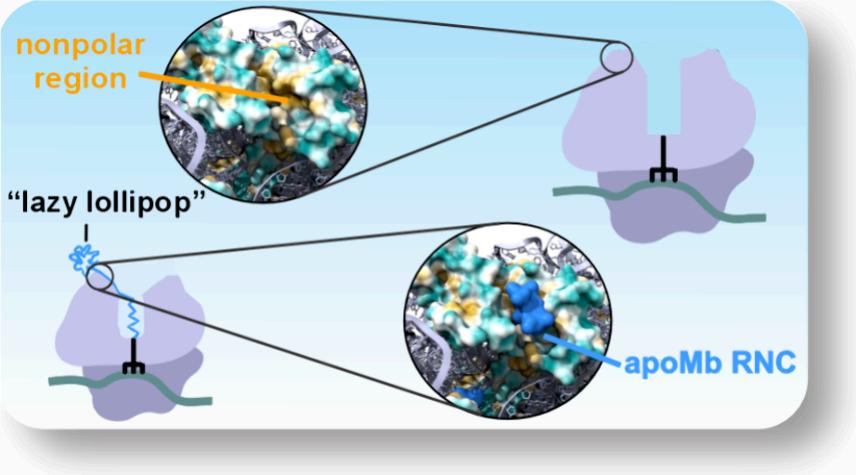

Interactions between ribosome-bound nascent chains (RNCs) and ribosomal components are critical to elucidate the mechanism of cotranslational protein folding. Nascent protein–ribosome contacts within the ribosomal exit tunnel were previously assessed mostly in the presence of C-terminal stalling sequences, yet little is known about contacts taking place in the absence of these strongly interacting motifs. Further, there is nearly no information about ribosomal proteins (r-proteins) interacting with nascent chains within the outer surface of the ribosome. Here, we combine chemical cross-linking, single-particle cryo-EM, and fluorescence anisotropy decays to determine the structural features of ribosome-bound apomyoglobin (apoMb). Within the ribosomal exit tunnel core, interactions are similar to those identified in previous reports. However, once the RNC enters the tunnel vestibule, it becomes more dynamic and interacts with ribosomal RNA (rRNA) and the L23 r-protein. Remarkably, on the outer surface of the ribosome, RNCs interact mainly with a highly conserved nonpolar patch of the L23 r-protein. RNCs also comprise a compact and dynamic N-terminal region lacking contact with the ribosome. In all, apoMb traverses the ribosome and interacts with it via its C-terminal region, while N-terminal residues sample conformational space and form a compact subdomain before the entire nascent protein sequence departs from the ribosome.

## Abstract 2

Single-particle cryogenic EM and fluorescence anisotropy decays show that a nascent globin interacts with the L23 ribosomal protein. The interaction site is highly nonpolar, suggesting a chaperone behavior.

## Introduction

Most previous studies on protein structure formation targeted understanding protein folding *in vitro*, upon refolding from denaturant, or following a temperature jump.^1−5^ The mechanism of protein folding within the cellular context, however, remains poorly understood.^6−11^ The ribosome is an essential biomolecule in the cell,^12,13^ and it is responsible for both peptide bond formation and protein folding during the early stages of a protein life. Despite the fact that the experimental modalities of cotranslational protein folding are still largely unknown,^6,14^ it is estimated that at least 30% of the *Escherichia coli* proteome folds cotranslationally.^15^ Given the importance of the ribosome in protein life and the paucity of high-resolution studies on ribosome-bound nascent chains,^16,17^ it is of compelling urgency and general significance to better understand the structural features of the complex translational machinery.

First and foremost, the ribosome catalyzes the formation of backbone amide bonds within the peptidyl transferase center (PTC). As translation proceeds, the nascent polypeptide progresses through the ribosomal exit tunnel. In addition, due to its peculiar funnel-like geometry that includes a ∼80 Å long tunnel core and a ∼20 Å long vestibule close to the outer surface,^18^ the ribosomal exit tunnel^19−22^ poses dramatic geometrical constrains to the conformational sampling of nascent polypeptides. The 30–40 residue C-terminal region of nascent proteins, typically buried within the tunnel core, has a natural tendency to attain some structure due to entropic arguments,^23^ and it sometimes populates local regions of native-like or non-native α-helical secondary structure.^22,24−28^ In the case of highly thermodynamically stable proteins, single domains can even acquire a native-like structure within the exit tunnel core.^29^ The exit tunnel vestibule and nearby outer ribosomal surface, on the other hand, are wider than the tunnel core and more generally support nascent chain conformational sampling, leading to tertiary structure formation. In the case of small- to medium-size single-domain proteins, these areas of the ribosome host compact nascent-chain subdomains^30−32^ that bear a native-like tertiary structure.^33^ Alternatively, in case the nascent chain bearing a C-terminal linker spanning ca. 7–35 residues, entire folded-like domains can be hosted.^34−36^ The C-terminal linkers enable most (or all) residues comprising the protein domain to interact with each other and generate the native state within the vestibule and upper regions of the tunnel. The ribosome has also been found to increase nascent chain solubility^30,37^ and to either destabilize^38^ or stabilize nascent chains^39^ relative to their native state. Further, the compact domains found in connection with the cotranslational folding of single-domain proteins are extremely spatially biased^30,37,40−42^ by bearing small-amplitude motions and are characterized by higher conformational dynamics than ribosome-released native states. In the case of intrinsically disordered nascent proteins, no compact regions are observed in any tunnel or outer ribosomal region, yet the ribosome participates in the process by severely limiting nascent chain conformational dynamics relative to ribosome-released proteins.^43−46^

The rate of translation is known to be affected by mRNA local structure, tRNA abundance, and codon composition, including rare codons.^47^ On the other hand, translation rates can also be modulated by the ribosome itself, especially when it interacts with nascent chains. For instance, positively charged regions of nascent proteins slow down translation rates and cause pausing^48^ as they progress through the ribosomal exit tunnel.^49,50^ This electrostatic effect is facilitated by the increasingly negatively charged potential across the wall of the ribosomal tunnel toward the exit site region.^51^

Interactions between ribosomal RNA (rRNA) or ribosomal proteins (r-proteins) and nascent chains have recently been found to play an increasingly important role. This is especially true in the case of nascent chains bearing C-terminal stalling sequences. For instance, r-proteins L22 and L4 and rRNA near the peptidyl transferase center (PTC) interact with nascent chains bearing C-terminal stalling residues.^52,53^ These interactions can, in turn, alter the geometry of the peptidyl transferase center (PTC)^54^ or even induce an α-helical secondary structure that contributes to translational stalling.^55^ Conversely, in the presence of nascent chains bearing N-terminal membrane-tagging signal sequences, proximity with L23, L24, and L29 r-proteins has been detected in the tunnel vestibule,^26,33,35,36^ though the authors did not explicitly quote the presence of actual interactions with these r-proteins. In the case of nascent chains lacking N- or C-terminal signal or stalling sequences, chemical cross-linking revealed the presence of interactions with specific ribosomal proteins (r-proteins) L23 and L29 (for ribosome-bound nascent chains (RNCs) of an intrinsically disordered protein)^45^ or only with L23 (for RNCs of *E. coli* apoHmpH, a foldable single-domain globin).^31^

Despite all of the above knowledge, which highlights the vital role played by the ribosome in protein biosynthesis and folding, crucial information on the specific r-protein and rRNA interaction sites, especially across the vestibule and outer ribosomal surface, is missing. Further, the nature of the ribosome–RNC interactions is not clear. This information is necessary to fully understand cotranslational folding and to devise future strategies to *ad hoc* reprogram its course.

In this work, we begin addressing the above lack of knowledge by determining the single-particle cryo-electron microscopy (sp-cryo-EM) structure of a full-length apomyoglobin (apoMb) RNC in the absence of any C-terminal stalling sequences or linkers. The work is carried out in the absence of the trigger factor (TF) chaperone and in the presence of a DnaK inhibitor (Hsp70 chaperone system) to rule out chaperone involvement. RNC generation in the presence of the zero-length cross-linker 1-ethyl-3-(3-(dimethylamino)propyl)carbodiimide hydrochloride (EDC) was crucial to obtain a density map revealing nascent chain interactions with specific regions of the ribosomal surface. Our results show that apoMb RNCs attain a predominantly extended conformation inside the ribosomal exit tunnel core, where they interact with rRNA and with the L22 and L4 r-proteins. In addition, we detected unprecedented interactions with r-protein L23 within the vestibule and outer ribosomal surface. Interestingly, this RNC interaction site of L23 is highly nonpolar, suggesting a chaperone role for this r-protein. A large portion of the N-terminal chain density is undetectable by sp-cryo-EM but is captured by fluorescence anisotropy in the frequency domain.^46^ This N-terminal RNC region is compact, and it comprises 68–83 residues and spans a cone semiangle of ca. 15°. In all, this study highlights the importance of a specific nonpolar region of the L23 r-protein for the cotranslational folding of single-domain proteins. In addition, our findings show that the ribosome enables protection of nascent chain nonpolar regions, much like a molecular chaperone. At the same time, the ribosome also permits a significant degree of cotranslational compaction, thereby supporting independent conformational sampling of ca. 50% of its sequence and thus promoting key steps in protein birth.

## Results and Discussion

### Experimental Design

The goal of this study is the identification and characterization of the structured and dynamic regions of a ribosome-bound nascent chain derived from a single-domain protein at the highest attainable degree of resolution. We adopted an integrative-biology approach, and our experimental design included the preparation of pure ribosome–nascent chain complexes devoid of C-terminal linkers or arrest sequences.^56^ RNCs were generated in the absence of molecular chaperones^56,57^ so that we could focus on identifying the effect of the ribosome alone on nascent-protein structure and dynamics. RNCs were then cross-linked (see below) to identify interacting ribosomal proteins at low resolution. High-resolution single-particle cryo-EM analysis followed. Finally, previously acquired^46^ frequency-domain fluorescence anisotropy decay experiments were critically evaluated to help identify N-terminal dynamic regions that were undetectable by sp-cryo-EM.

For the correct rendering of a 3D structure, single-particle cryo-EM (sp-cryo-EM) requires multiple 2D projections. As a consequence, highly dynamic macromolecular regions, which are heterogeneously represented within 2D projections, are typically hard to characterize.^58^ Ribosome-bound nascent chains (RNCs) encoding single-domain proteins and lacking C-terminal linkers or arrest sequences are known to be highly dynamic.^30,42,43,46,59,60^ This feature highly complicates the structural analysis of RNCs, especially outside the ribosomal exit tunnel core. In this work, we introduce zero-length chemical cross-linking^61,62^ as a strategy to partially overcome this challenge. While chemical cross-linkers have been previously used to facilitate 3D structure determination of dynamic macromolecules,^63^ this approach has not been utilized for RNCs. The zero-length chemical cross-linker EDC has previously been used to identify the interaction of r-proteins with nascent chains,^31,45^ rendering it an ideal candidate for cross-linking dynamic RNCs to the ribosomal surface. Given that (i) nascent chains only cross-link when they are in close proximity to the ribosome,^45^ (ii) prior kinetic studies showed that the majority of nascent chain and r-protein interactions occur very quickly,^45^ and (iii) EDC tends to provide an underestimate of the actual populations of interacting RNCs,^45^ it is appropriate to regard the cross-linked structure presented here as appropriately capturing the presence of RNC–r-protein contacts. Note that EDC does not cross-link to RNA under the imidazole-free conditions employed here.^62^ Therefore, RNC–rRNA contacts detected in this work are not influenced by the presence of this cross-linking agent.

**Figure 1:**
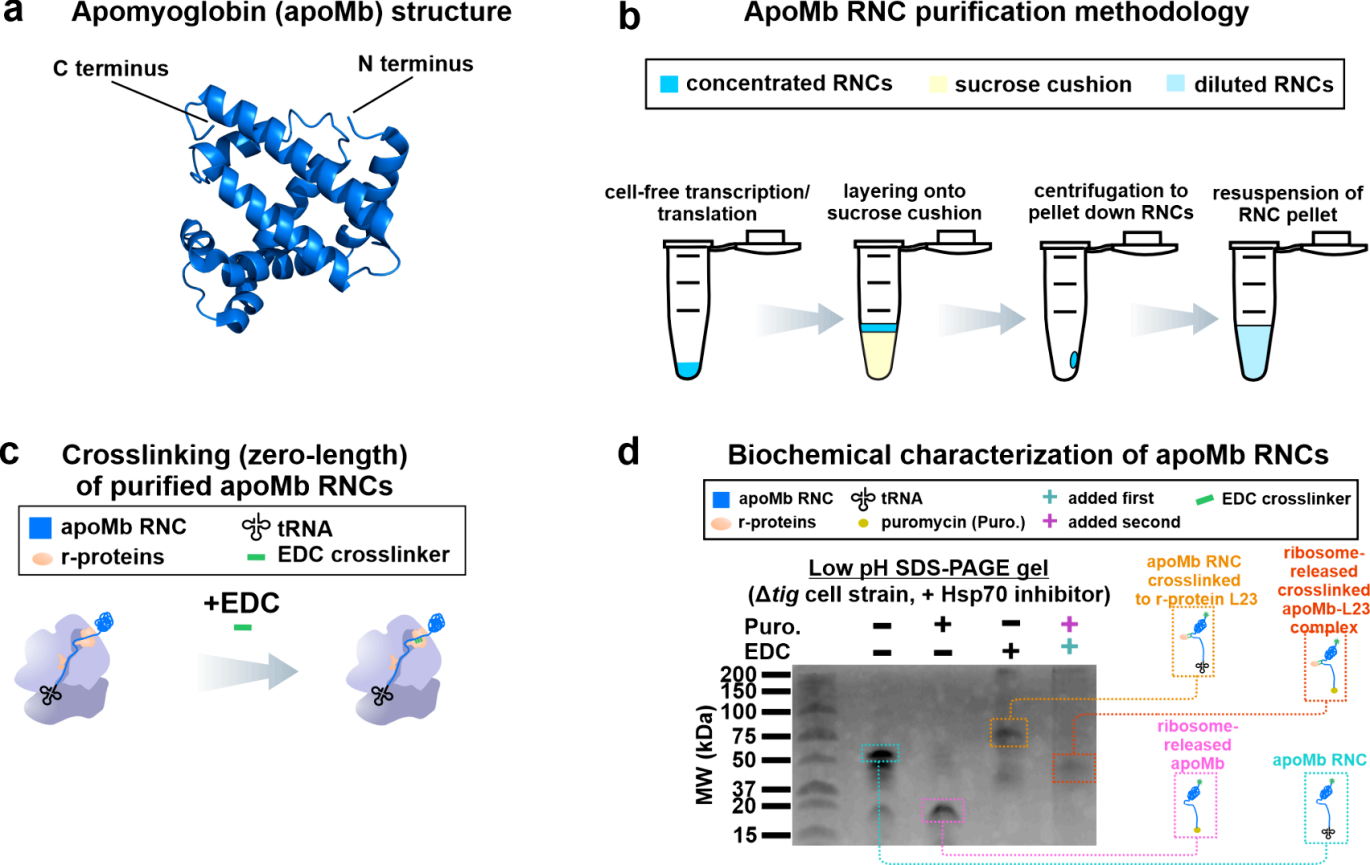
Structure of the single-domain model protein analyzed in this work and generation of ribosome-bound nascent chains (RNCs). (a) Structure of sperm whale myoglobin (apoMb), i.e., the holo-form of the model single-domain foldable protein analyzed in this work (PDB ID 1MBC). (b) Scheme illustrating the adopted purification procedure to generate apoMb RNCs for sp-cryo-EM analysis. (c) Schematic representation of the post-RNC purification treatment with the zero-length EDC cross-linking agent. (d) Representative low-pH SDS-PAGE gel illustrating RNC chemical cross-linking via EDC (*N* = 2).

The nascent chain analyzed in this work bears the full-length apomyoglobin (apoMb) amino acid sequence. apoMb is a single-domain and medium-size (ca. 17 kDa) folded protein that shares a similar abundance and amino acid composition with the *E. coli* proteome.^64^ In addition, apoMb carries the ubiquitous globin fold, has a well-defined structure (Figure 1a),^65−68^ and has been extensively characterized from both the structural, folding/unfolding/aggregation and biochemical standpoints *in vitro*.^69−73^ The apoMb sequence employed in this work is from *Physeter catodon* (sperm whale), and the ribosomes are from *E. coli*. Therefore, the data presented here serve as a model for conformational sampling pertaining to heterologous protein expression. This work is also generally representative of the structural features of ribosome–RNC complexes, where the RNC portion pertains to single-domain foldable proteins. It is worth noting that the employed gene sequence encoding apoMb has a codon usage optimized for *E. coli*.^74^ As shown in Figure 1b, apoMb RNCs were purified via a sucrose cushion followed by cross-linking to r-proteins via EDC (Figure 1c) before being applied to a cryo-EM grid and promptly vitrified. Data were processed via the CryoSPARC (v. 3.8–4.2) software^75^ according to routine procedures (see Materials and Methods). After routine processing, we employed 3D variability analysis to tease out particles, including p-site tRNA and nascent chain density. Particles containing the p-site tRNA were used to yield a sp-cryo-EM structure bearing an average resolution of 2.91 Å, before low-pass filtering at 4 Å via Relion,^76^ to best resolve nascent chain density. The low-pass filter cutoff was determined by systematically applying low-pass filtering from 3 to 5.5 Å within 0.5 intervals. Low-pass filtering removes the signal of higher resolution than the filter value. Therefore, we sought to achieve an optimal balance between identification/resolution of nascent chain density and the resolution of the non-RNC portion of the 70S ribosome structure.

### Nascent apoMb Interacts With R-Proteins

The presence of noncovalent contacts between RNCs and the L23 r-protein were previously detected for the nascent intrinsically disordered protein (IDP) PIR^45^ and for the nascent *E. coli* globin apoHmpH^31^ via a combination of EDC cross-linking and Western blotting. Here, we find that apoMb RNCs display an EDC-cross-linking gel-band pattern qualitatively similar to those of apoHmpH and PIR, as shown in Figure 1d. The RNC gel band detected in the presence of EDC bears a higher molecular weight by about 20–28 kDa. This result is consistent with the presence of cross-linking to either one large r-protein or to multiple smaller-size r-proteins. The molecular weight distribution of *E. coli* r-proteins is readily available in the literature and can be found, for instance, in the Supporting Information of a study by Guzman-Luna et al.^77^ Based on these values, interaction with only one of the plausible large r-proteins L1 and L2 (24.6 and 29.7 kDa, respectively) seems unlikely, as these proteins are not located in proximity of the ribosomal exit tunnel. A control experiment including the addition of EDC after puromycin-mediated RNC ribosome release shows no cross-linking (Figure 1d). The latter experiment demonstrates that the detected interactions occur only when the apoMb chain is in close contact with the ribosome as a RNC. The data presented above prove that apoMb RNCs interact with one or more r-proteins of a total 20–28 kDa size. The sp-cryo-EM results, shown in the sections below, indicate that the interacting proteins are L23 and L29. The respective sizes of these r-proteins are 11.2 kDa and 15.8 kDa. Given that these two values sum up to 27 kDa, the results from low-pH SDSP-PAGE (Figure 1d) and from sp-cryo-EM (see sections below) are entirely internally consistent and show that apoMb RNCs interact with both the L23 and L29 r-proteins.

### Apomyoglobin has a Predominantly Extended Conformation within the Ribosomal Exit-Tunnel Core

**Figure 2:**
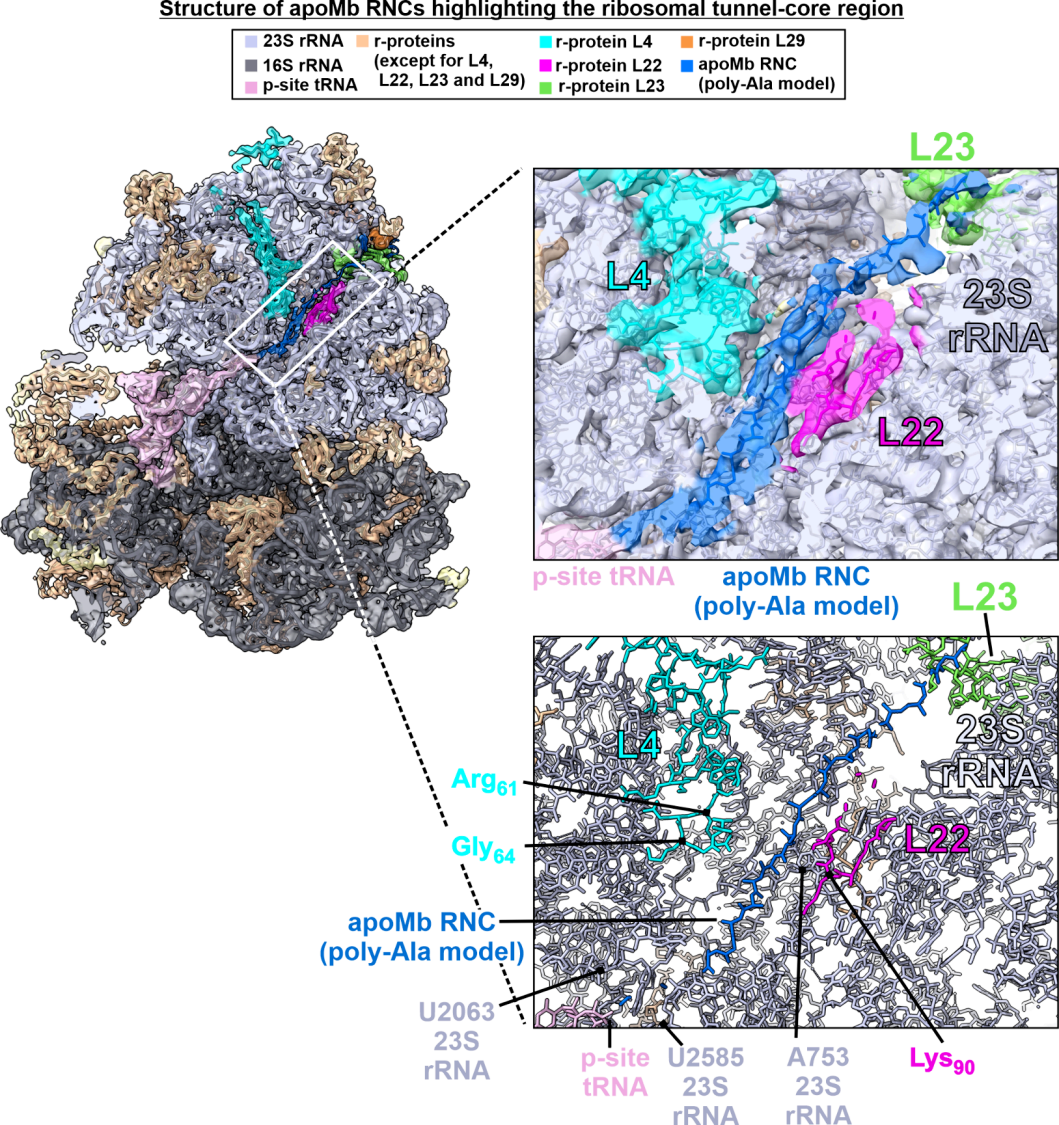
Single-particle cryo-EM structure of apoMb RNC–ribosome complex with emphasis on the ribosomal tunnel core. (a) Semitransparent cryo-EM density and corresponding structural model of the apoMb RNC–ribosome complex. The nascent chain density is shown in blue. (b) Enlarged box showing nascent chain density (blue) traversing the ribosomal exit tunnel core. (c) Structural model corresponding to the cryo-EM density of panel b.

We then proceeded to determine the structure (2.9 Å average resolution) of apoMb RNCs by sp-cryo-EM (details in Materials and Methods). The overall structure of the ribosome/apoMb-RNC complex is shown in Figure 2a. Given that the local resolution provided by CryoSPARC for the RNC-chain portion of the structure is only 6–7 Å, no information about side chains can be deduced. Therefore, we modeled the RNC density as a simple poly-Ala chain (Figure 2b and c). As a consequence, in the entirety of this study, the detected interactions are solely assessed upon estimating distances between the poly-Ala-modeled RNC and density arising from the ribosome.

The structural features of apoMb RNCs were first analyzed within the exit tunnel core (Figure 2b and c). Within this region, the apoMb RNC is overall unstructured, with widely distributed backbone dihedral angles suggesting a mostly extended chain (Suppporting Figures S6 and S7). In addition, we detected interactions between RNCs and the 23S rRNA (rRNA) near the PTC site, including well-known interacting regions near the U2585 ribonucleotide^33,55,78−80^ and near the C2063 and G2505 ribonucleotides.^33,79^ It is worth noting that the nascent chain density within the tunnel core region close to the PTC is better resolved than that in regions further up across the ribosomal exit tunnel core, vestibule, and outer ribosomal surface. This increase in local motion is likely a direct result of the extensive interactions between the nascent chain and rRNA at the PTC.

Proceeding toward the central region of the ribosomal-tunnel core, the apoMb RNC is found to interact with r-proteins L4 (at Gly64 and Arg61) and L22 (at Lys90). In addition, interactions with the 23S rRNA nucleotides near A753 are also detected (Figure 2c).

Interestingly, identical RNC-interacting regions along the *E. coli* ribosome tunnel core were previously reported in the context of entirely different nascent chain sequences, i.e., all β-sheet proteins and ribosome-stalling polypeptides.^33,55,79,80^ The recurring presence of these interaction sites across a variety of RNCs highlights their likely importance, regardless of the nascent chain sequence. Finally, as the nascent chain traverses the ribosomal exit tunnel into the spatially wider vestibule region, an increase in RNC dynamics is observed, as detailed in the next section.

### A Small Compact Region of apoMb RNCs Interacts with R-Protein L23 and with A63 23S rRNA within the Tunnel Vestibule

**Figure 3:**
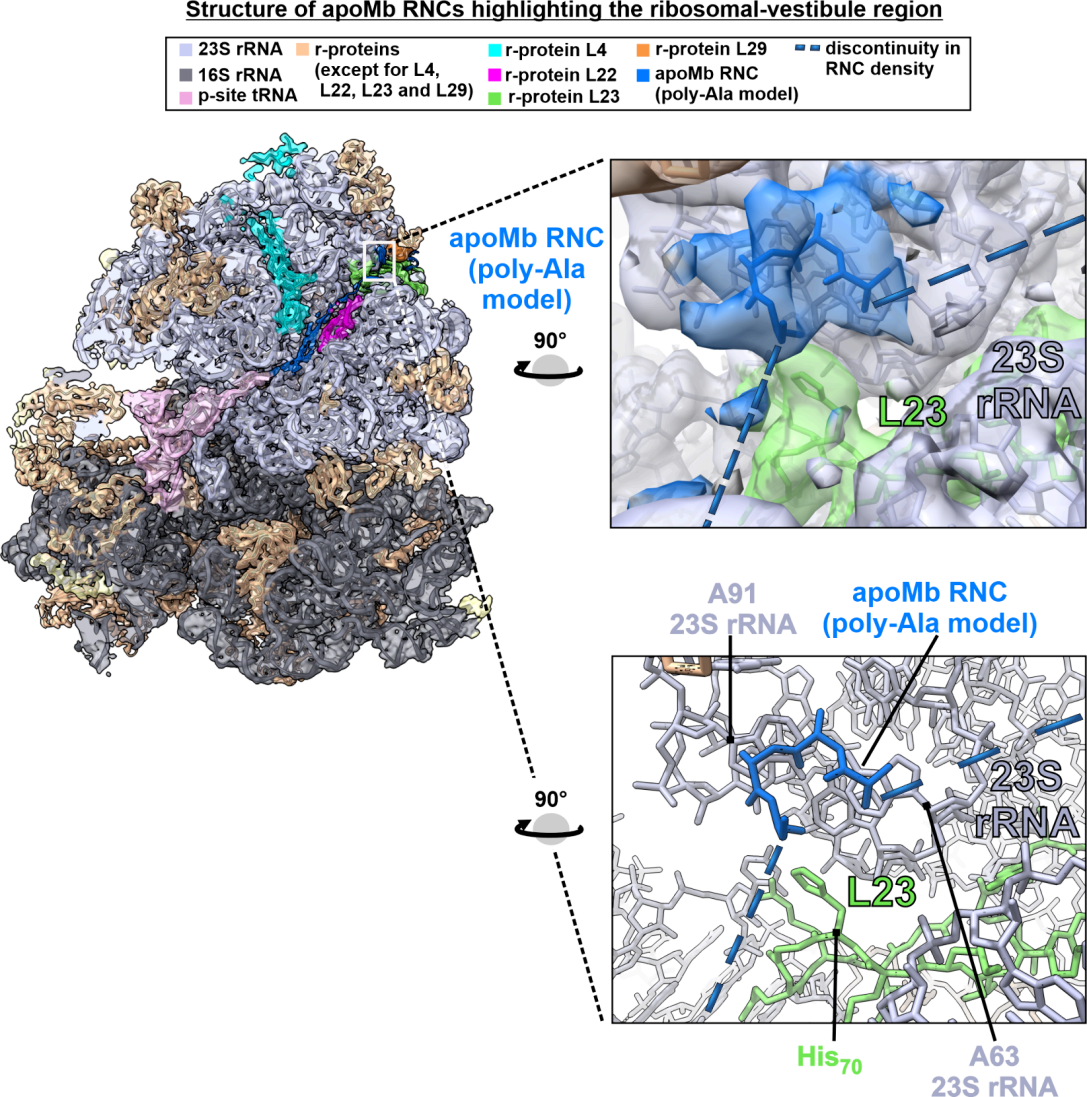
Single-particle cryo-EM structure of the apoMb RNC–ribosome complex with emphasis on the ribosomal vestibule. (a) Semitransparent cryo-EM density and corresponding structural model of the apoMb RNC–ribosome complex. The nascent chain density is shown in blue. (b) Enlarged box showing nascent chain density (blue) traversing the ribosomal vestibule. (c) Structural model corresponding to the cryo-EM density of panel b.

Moving toward the exit tunnel vestibule region of the ribosome, we were able to identify additional features of the apoMb RNC structure, including additional interactions with the ribosome. Figure 3 shows that the apoMb RNC (modeled as poly-Ala) bears a small compact region comprising ca. 4–5 residues resembling a turn. Interactions between the apoMb nascent chain and r-protein L23 (at Gln72 and His70) were also detected (Figure 3c). In addition, the nascent chain interacts with a region of the ribosome near nucleotides A91 and A63 (Figure 3c). While we should note that there may be additional existing interactions, the detected pattern suggests that the L23 r-protein serves to thread nascent apoMb along its surface, right past the exit tunnel core. Both sides of the small compact region bear the apoMb RNC also bear two flanking dynamic regions with interrupted density, denoted as blue dashed lines in Figure 3b and 3c. Clearly, nothing can be stated about the conformational nature of these regions based on this sp-cryo-EM structure. On the other hand, both of these regions are flanked by detectable RNC density, and all detectable RNC and ribosome densities are clearly distinct. Thus, it is evident that our apoMb RNC is characterized by overall uneven local dynamics and by interactions with rRNA and the L23 r-protein region that faces the exit tunnel vestibule.

### ApoMb RNCs Interact Primarily with a Nonpolar Region of the L23 R-Protein

The structure of RNCs of full-length single-domain proteins (8–18 kDa) lacking C-terminal linkers or added residues is very poorly understood at present. The only known example is CspA, a small (70 amino acids) all-β-sheet ca. 7.4 kDa *E. coli* protein.^24^ However, no high-resolution information is available about the structurs of RNCs representing larger nascent chains, including medium-sized single-domain proteins of 12–18 kDa. Yet, this information is sorely needed because the latter size range is highly representative of individual domains across proteomes of multiple organisms.^81−84^ Here, thanks to the EDC cross-linker’s ability to capture interacting moieties, we were able to identify some unique features of the apoMb nascent chain. Indeed, this RNC is 17.3 kDa in size; hence, it is representative of a typical protein domain.

**Figure 4:**
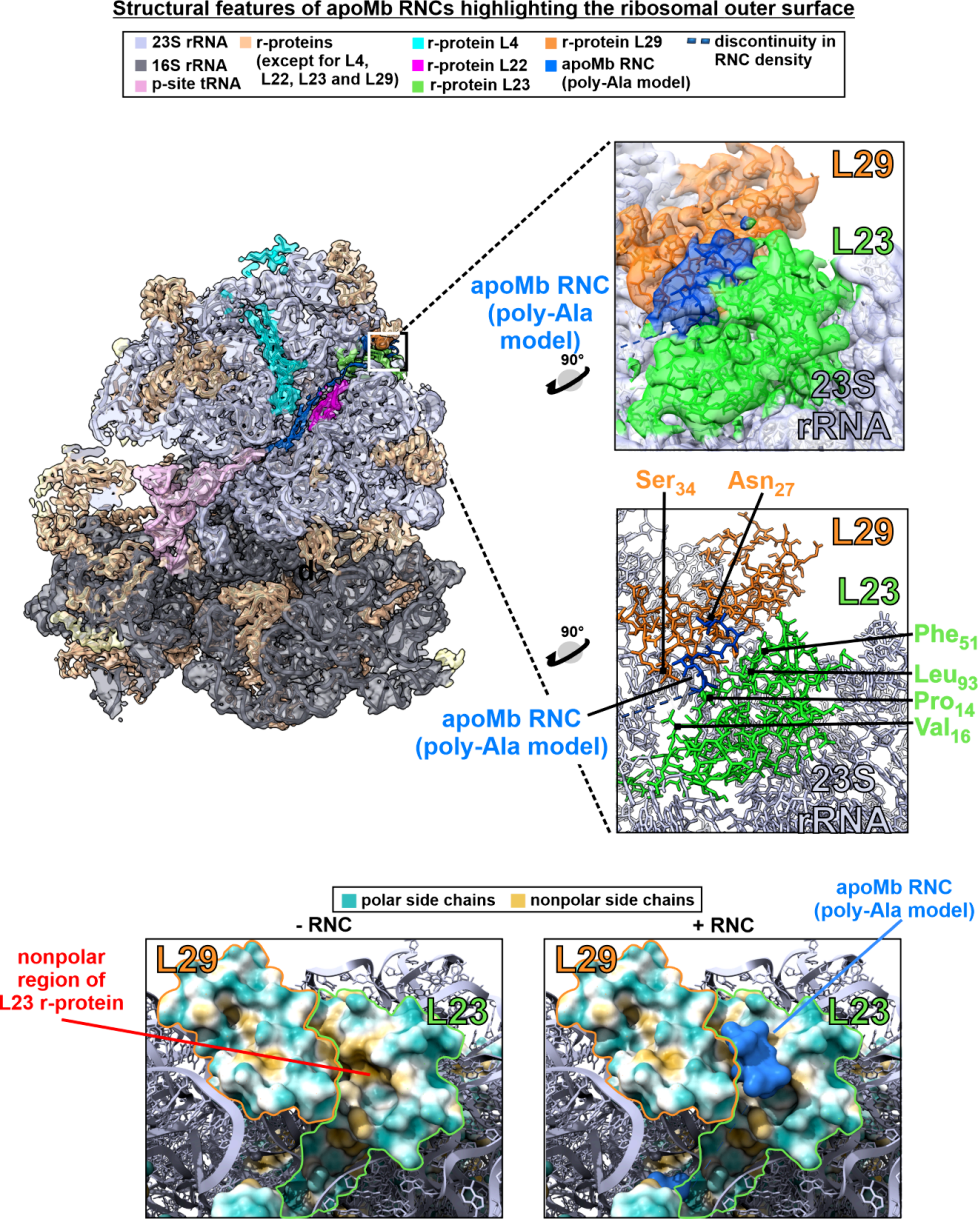
Single-particle cryo-EM structure of the apoMb RNC–ribosome complex with emphasis on the ribosomal outer surface close to the tunnel exit. (a) Semitransparent cryo-EM density and corresponding structural model of the apoMb RNC–ribosome complex. The nascent chain density is shown in blue. (b) Enlarged box showing nascent chain density (blue) interacting with the outer ribosomal surface close to the tunnel exit. (c) Structural model corresponding to the cryo-EM density of panel b. (d) Side view of sp-cryo-EM map focusing on the L23 and L29 r-proteins facing the outer ribosomal surface, with nonpolar and polar residues rendered in orange and cyan, respectively, and with RNC density omitted. Color coding of polar and nonpolar residues was carried out in ChimeraX according to the scale by Kyte and Doolittle.^137^ (e) Same image as in panel d, except that the RNC density is explicitly shown (in blue).

First, some clear cryo-EM density comprising ca. 8 residues could be captured in close proximity (3–8 Å backbone-to-backbone) of the L23 and L29 r-protein regions facing the outer surface of the ribosome (Figure 4a). The corresponding cryo-EM density and backbone trace are rendered in blue and are best visualized in Figure 4b and c, respectively. Note that all previously detected interactions involving RNCs of other proteins and the ribosome were limited to the tunnel core and tunnel vestibule regions. Hence, this study shows the first example of an interplay between RNCs and a specific protein region on the outer surface of the ribosome.

Second, the high local resolution of the L23 and L29 r-proteins (ca. 3 Å, generated via CryoSPARC) enabled the assessment of the specific residues of these proteins interacting with apoMb RNCs, namely, Pro14, Val16, Leu93, and Phe51 of L23 and Ser34 and Asn27 of L29 (Figure 4c and Supporting Figure S8). The RNC-interacting region of L23 is particularly interesting, given that the relevant residues have highly nonpolar side chains. As shown in panels d and e of Figure 4, the RNC-interacting L23 region bears a well-defined nonpolar cavity. Therefore, we propose that the interaction between L23 and the apoMb RNC is significantly contributed by the hydrophobic effect.

Interestingly, it was shown in 2009 that nonpolar cavities tend to be solvent-depleted, i.e., dewetted.^85,86^ We reason that it is possible that the L23 nonpolar cavity is dewetted and that this dewetting facilitates nonpolar-dominated interactions with RNCs. While the resolution of our structure is not sufficient to directly visualize apoMb side chains interacting with L23, the proximity of the RNC to the L23 nonpolar cavity is highly suggestive of the hydrophobic effect playing an important role. Thus, we speculate that the L23 r-protein may serve a chaperone-like function on the very surface of the *E. coli* ribosome.

### ApoMb RNCs Display Variable Local Dynamics as They Progress through the Ribosomal Tunnel and Ribosomal Outer Surface

**Figure 5:**
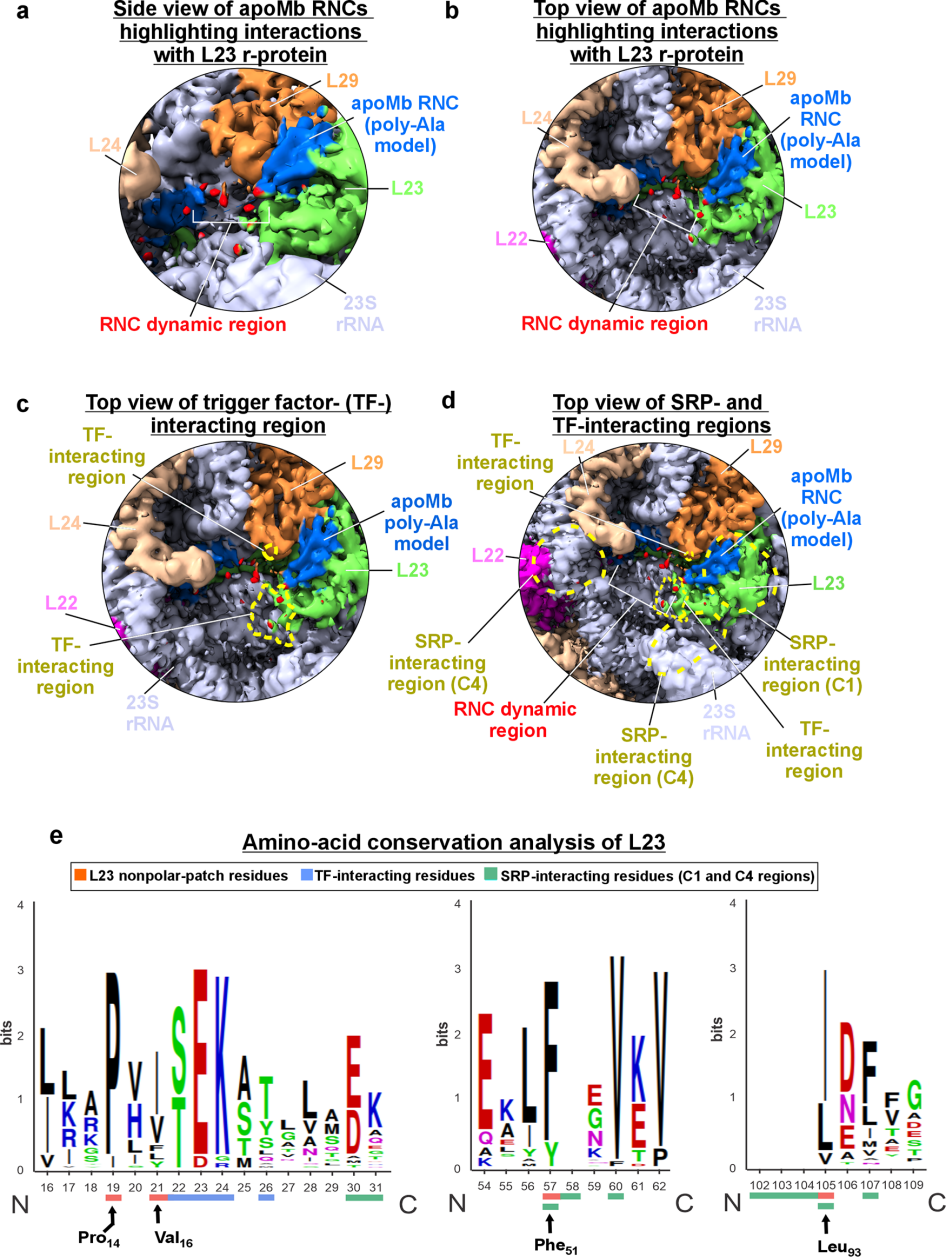
Single-particle cryo-EM density of the apoMb-RNC–ribosome complex focusing on an RNC dynamic region and docking sites for the trigger factor (TF) and signal recognition particle (SRP). (a) Side view of sp-cryo-EM map showing a low-signal portion of the map (rendered in red) likely corresponding to a dynamic apoMb RNC region. (b) Top view of image in panel a. (c) Mapping of TF docking sites across the outer ribosomal surface. (d) Same surface as in panel c with added dashed traces denoting the known SRP docking sites on the *E. coli* ribosomal outer surface. (e) Amino acid conservation analysis of the L23 ribosomal protein, highlighting the binding sites for apoMb RNCs (orange), TF (dim blue), and SRP (bright blue) (see details in Materials and Methods and Supporting Table S4 and Figure S11). The L23 residues comprising the nonpolar patch and interacting with the RNCs are labeled in black. This analysis was carried out upon comparing the amino acid sequence of *E. coli* L23 to those of all bacterial proteins in the DEG database for which the gene encoding L23 is essential.

While, as discussed in previous sections, some RNC regions have undetectable cryo-EM density, we were able to identify the pathway adopted by the apoMb nascent chain as it emerges from the ribosomal exit tunnel vestibule onto the ribosomal surface. About 6 Å away from any modeled structure, we detected a very weak and selectively localized signal, colored in red in Figure 5a and b, that directly connects the clearly detectable density reported in Figures 3b and 4b. We propose that this low-intensity signal is likely due to dynamic RNC regions within the intervening space connecting the regions of clearly detected RNC density.

Previous cross-linking-based experiments detected RNC interactions with L23^31,45^ and L29^45^ only at low resolution, and prior cryo-EM work only detected RNC-ribosome contacts within the ribosomal vestibule.^35,36^ In contrast, this study highlights for the first time the presence of RNC–r-protein contacts across the outer surface of the ribosome. In addition, our work shows that these interactions are competent to protect nonpolar regions of the nascent chain before all C-terminal residues have emerged from the ribosomal exit tunnel so that full conformational sampling can take place.

### Trigger Factor (TF) and apoMb RNCs Have Nearby Nonoverlapping Interaction Sites Across the Ribosomal Surface

As described in the previous sections, the L23 and L29 r-proteins interact with ApoMb RNCs. The L23 r-protein carries a major role due to its more numerous interacting residues and due to the predominantly nonpolar nature of the interaction site, which may facilitate the burial of RNC nonpolar residues. Interestingly, it is well-known that the L23 and L29 r-proteins also serve as the docking sites for the trigger factor (TF) chaperone, with the L23 residues Glu18 of L23 regarded as essential for TF’s function in the cell.^87^ It is therefore interesting to compare the L23 and L29 interaction sites of apoMb RNCs with those of TF.

Before proceeding with a comparison of RNC and TF docking sites, we should specify upfront that the RNC–ribosome structure reported here was determined in the absence of the TF (Materials and Methods). On the other hand, translation outcomes of wild-type apoMb are very similar in the absence and presence of TF, with the only detectable difference being the presence of ca. 30% fewer soluble aggregates when TF is present.^30^ Previous studies showed that the interaction of full-length nascent *E. coli* globin domains with ribosomal proteins are mostly displaced by interactions with TF at physiologically relevant TF concentrations (ca. 8–20 μM) in purified RNCs resuspended in buffer.^31^ On the other hand, competition for TF is expected to be fierce within the complex live-cell environment of *E. coli*, given the many types of RNCs present in the cytosol, the comparable TF and active ribosome concentrations, the fact that multiple TF molecules may bind single RNCs, and the variable affinity of TF for nascent protein chains.^77,88^ Therefore, we expect that some RNCs are TF-free in the cellular environment. As a consequence, the RNC mode of binding in the absence of TF, reported here, is likely to be relevant to the natural environment of live bacterial cells.

The TF site on the *E. coli* ribosome is mapped as yellow dashed curves in Figure 5c. This site is identified based on recent structural work that includes the entire TF molecule bound to the *E. coli ribosome*.^89^ While this structure includes an additional ribosome-bound factor (PDF) docking on a different region of the ribosome, the TF docking sites are entirely similar to those reported for the TF ribosome-binding domain on the eubacterial *Deinococcus radiodurans* ribosome.^90,91^

Interestingly, upon comparison of the TF docking site and bound apoMb RNC location in Figure 5c, it is evident that the two ligands (TF and RNCs) do not share the same binding site, although the binding regions are close in space. On the other hand, there are two nearby TF docking sites on L23/29 that virtually “cross” the RNC’s progression from the vestibule to the outer ribosomal surface. This spatial arrangement makes it difficult to imagine how apoMb RNCs could concurrently bind to the L23/L29 site detected here (on the outer surface of the ribosome) and to a ribosome-bound TF chaperone. Therefore, the data available at present suggest that the two binding sites may be mutually exclusive and that, in the presence of TF, RNCs may either bind the outer ribosomal surface or the TF chaperone. Future high-resolution studies in the presence of both RNCs and TF are needed. These investigations will hopefully be able to shed further light on this topic.

### The Signal Recognition Particle (SRP) and apoMb RNCs Have Overlapping Interaction Sites Across the Ribosomal Surface

In addition, the signal recognition particle (SRP) has multiple docking sites on the *E. coli* ribosome, as shown in Figure 5e.^92^ Interestingly, the major SRP docking site does not overlap with the TF binding sites on the ribosome, consistent with the biochemically detected concurrent TF/SRP binding,^93^ but it does overlap with the RNC docking region. This result suggests that nascent chain binding to the outer ribosomal surface and to SRP may be mutually exclusive. Additional studies are needed to shed light on this possibility.

In any case, under the conditions of the present studies, SRP is likely absent due to the following arguments. First, SRP does not extensively interact with nascent chains lacking a signal sequence, including the apoMb RNCs examined here.^94^ Second, SRP has a very low binding affinity for ribosomes carrying RNCs lacking a signal sequence.^95^ Third, the SRP concentration in *E. coli* cells (ca. 400 nΜ)^96^ is much lower than a typical ribosome concentration (ca. 20 μM).^97^ Lastly, in support of the lack of SRP in our system, the multiple known SRP docking sites on the *E. coli* ribosome, mapped in Figure 5e, would imply likely detection of additional nonribosome cryo-EM density at these additional binding-site locations. Given that no trace of such additional density was found and in consideration of all the above arguments, we conclude that the cryo-EM density reported here arises from the apoMb nascent chain and is not due to SRP.

### The Nonpolar Nascent Chain Interaction Site of L23 is Highly Conserved

Conservation analysis was performed on the L23 ribosomal protein to assess whether the primary site of interaction of L23 with the nascent chain, which is highly nonpolar (Figure 4), is shared among different organisms. The gene encoding the L23 ribosomal protein is essential for cell viability in *E. coli*.^98^ Keeping this in mind, we carried out the conservation analysis across all bacteria of a database known as DEG (Database of Essential Genes),^99,100^ which contains genomes of organisms that bear one or more essential genes. Interestingly, in most of the bacteria belonging to the DEG database, the gene encoding L23 is also essential.

Therefore, we initially focused on comparing the bacteria in the DEG database, where L23 serves as an essential protein. The results are shown in Figure 5e, Supporting Figure S11 and Supporting Table S4. First, the data in Supporting Table S4 and Supporting Figure S11, which displays the entire output, show that L23 bears both unconserved, moderately conserved, and highly conserved regions. This observation suggests that specific portions of this protein (i.e., the highly conserved ones) play a well-defined role that is recurrent among species. Second and most importantly, Figure 5e shows that all residues comprising the nonpolar region of L23 that interacts with the RNC (i.e., Pro14, Val16, Phe51, and Leu93, see also Figure 4c) are highly conserved. Third, unsurprisingly, the interaction sites with the TF chaperone are highly conserved. Finally, the major interaction regions with SRP are only partially conserved. The origin of the latter finding deserves further investigation but is beyond the scope of this study.

Second, we also performed conservation analysis upon comparing *E. coli* L23 to all L23s in bacteria (within the DEG database) where L23 is a nonessential protein. The results, shown in Supporting Table S5 and Supporting Figure S12, show a conservation pattern very similar to the one discussed in the previous paragraph. We deduce that the Pro14, Val16, Phe51, and Leu93 residues of *E. coli* L23 preserve a very similar physical character across all bacteria in the DEG database, regardless of the essential nature of the L23 protein.

In summary, our conservation analysis shows that the residues comprising the nonpolar RNC interaction site of *E. coli* L23 are highly conserved across the bacteria in the DEG database. This finding suggests that the L23 nonpolar site may serve a specific function in both *E. coli* and other bacteria.

### Ribosome-Bound apoMb Nascent Chains in the Presence of EDC Have an N-Terminal Compact Region

Interestingly, the detectable cryo-EM density modeled as a poly-Ala chain accounts for ca. 34 residues, and the missing-density regions account for an additional ca. 5 + 10 = 15 residues, assuming an extended chain. Therefore, the apoMb RNC region within the ribosomal tunnel, vestibule, and bound to the outer ribosomal surface accounts for a total of at least 49 residues. While this is a lower estimate, this number strongly suggests that a considerable N-terminal nascent protein density is missing from our analysis. It is likely that the missing density is due to the highly dynamic nature of this N-terminal nascent chain region.

**Figure 6:**
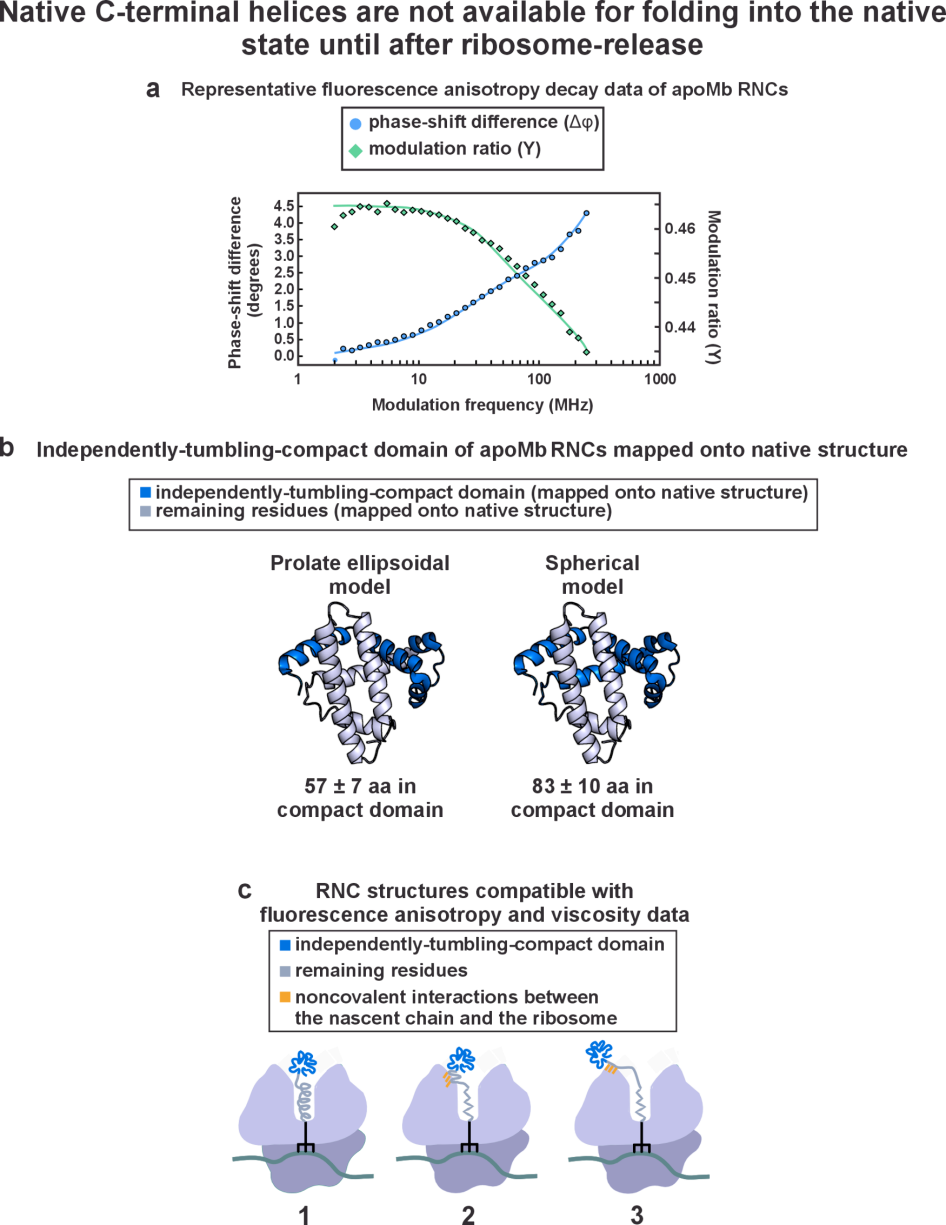
Fluorescence-anisotropy decays reveal that apoMb nascent chains have a compact N-terminal region. (a) Representative frequency-domain fluorescence anisotropy decay^46^ data of full-length wild-type apoMb RNCs. (b) Expected size (rendered in dark blue) of an independently tumbling compact N-terminal subdomain of apoMb RNCs mapped onto the native Mb structure. (c) Models of apoMb RNCs consistent with fluorescence anisotropy decay data: Model 1 is incompatible with the sp-cryo-EM data presented in this work. Models 2 and 3 are feasible, though the sp-cryo-EM data show that apoMb RNCs interact with both tunnel vestibule and outer ribosomal surface regions. Data in this figure are reproduced with permission (and modified, in the case of panel c) from ref (42). Copyright 2021 American Chemical Society.

**Figure 7:**
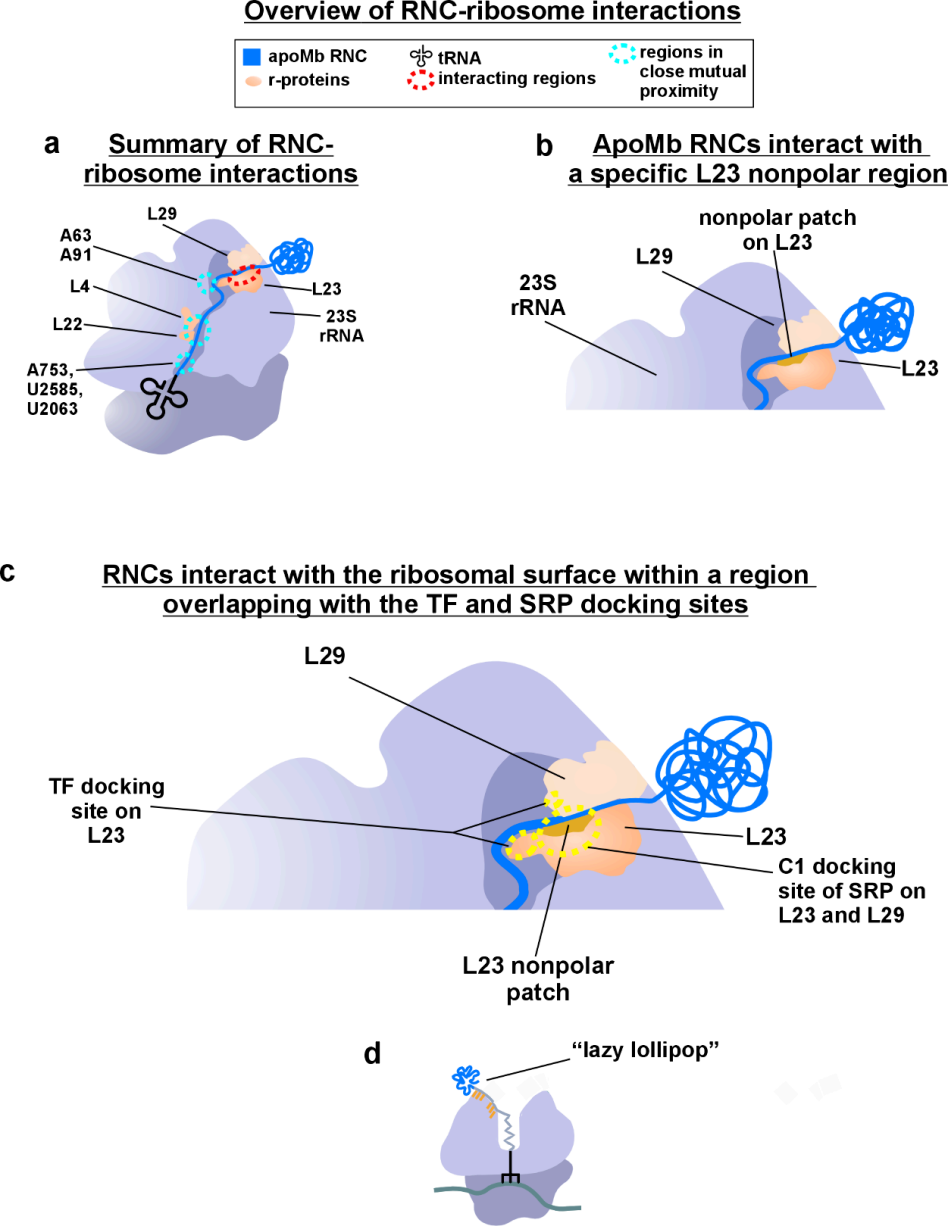
Cartoons illustrating the main findings of this study. In all cartoons, the viewing angle is adjusted to collectively show interactions with the tunnel core, vestibule, and outer surface. (a) Graphical overview of apoMb RNC–ribosome interactions detected in this work. (b) Cartoon illustration of the nonpolar L23 patch that comprises most of the apoMb RNC docking site. (c) Pictorial representation of the apoMb RNC in the context of the known TF and SRP docking sites. (d) Overall model (“lazy lollipop”) deduced from the combination of sp-cryo-EM and fluorescence anisotropy decay data. The model highlights the fact that the RNC’s N-terminal region is compact and independently tumbling, while the C-terminal portion of the RNC interacts with the L23 ribosomal protein within both the vestibule and the outer surface of the ribosome.

Fluorescence anisotropy decay in the frequency domain is a technique that provides valuable complementary information on the rotational characteristics of dynamic N-terminal portions of RNCs.^46,101−103^ As long as RNCs are site-specifically labeled with an N-terminal fluorophore as described,^60^ the time scale and spatial amplitude of the RNC motions can be accurately mapped upon assessing rotational correlation times (τ_c_) and cone semiangles.^42,46,59,60^ In addition, in combination with microscale-volume viscometry, it is also possible to estimate the size of the tumbling unit, assuming a roughly globular shape of the tumbling unit.^42^Figure 6 reproduces published fluorescence depolarization data of apoMb RNCs.^42^ As shown in this figure, a non-ribosome-bound N-terminal dynamic region comprising 67–83 residues was detected. This compact subdomain and its size are fully consistent with the extent of the missing cryo-EM density. Further, the high order parameter (*S* = 0.947 ± 0.005)^42^ and small cone semiangle (15.3 ± 0.7°)^42^ of the detected nascent chain N-terminal compact subdomain are fully consistent with the spatially constrained environment close to the ribosomal outer surface implied by the cryo-EM structural analysis described here. While the original publication^42^ reported that either structures of type 2 or 3 in Figure 6c are feasible, the cryo-EM analysis shown in this work reveals that both types of structure are populated, in the sense that the nascent chain experiences interactions with the ribosome both within the tunnel and across the outer ribosomal surface. Corresponding cartoons, highlighting the resulting structural model, are shown in Figure 7a–c. In addition, Figure 7d highlights the overall take-home message, recapitulated by a simple model denoted as a “lazy lollipop”, according to which the N-terminal portion of the RNC is compact, while the C terminal region interacts extensively with the ribosomal surface.

In addition, we performed new experiments under the same conditions as in Figure 6 except that fluorescence lifetimes and anisotropy decays were determined in both the absence and presence of the EDC cross-linker. The results, shown in Supporting Figures S9 and S10, are qualitatively consistent with the data of Figure 6, including the recurrent identification of a compact N-terminal region tumbling with a low-nanosecond rotational correlation time. The apparent RNC number of residues comprising the compact region, however, was not realistic (ca. 155 residues) in the presence of EDC. We believe that this is an artifact due to some EDC intramolecular cross-linking modifying the detailed shape of the RNC’s N-terminal compact domain, consistent with prior observations.^77^

### The L23 R-Protein May Serve As a Molecular Chaperone and/or a Nascent Chain Solubilizing Region

Recent studies in reconstituted *E. coli* cell-free systems showed that, even in the absence of molecular chaperones, at least 28% of the nonmembrane *E. coli* proteome is biosynthesized in soluble form (i.e., it has ≥80% solubility).^104−106^ In addition, activity assays,^107−109^ antibody binding,^110^ and treatment with Lon-protease under controlled conditions^111^ showed that several newly synthesized proteins are not only soluble but actually reach their native state upon release from the bacterial ribosome in the absence of molecular chaperones. These proteins include dihydrofolate reductase (DHFR),^107^ λ-lysozyme,^107^ green fluorescent protein,^107^ firefly luciferase,^108,109^ tailspike,^110^ and several others.^111^ Additional recent work on apoMb revealed that the ribosome has a nascent chain solubilizing character.^30,59^ The above results show that the ribosome has a remarkable ability to support the solubility and folding of a variety of proteins even in the complete absence of external molecular chaperones. The findings presented above, in combination with the research presented here, suggest a link between the nonpolar character of the newly discovered L23 interaction site and the known nascent chain solubilizing action of the ribosome.

Undoubtedly, intrinsic properties of the protein sequence can also contribute to chaperone-free in-cell structure formation. These properties include the propensity of low-contact-order proteins to undergo vectorial cotranslational folding^6,106,112−114^ and the tendency of contiguous domains to fold cooperatively while ribosome-bound.^115^ Further, in some cases the translational machinery facilitates folding via cotranslational pausing and synonymous codon substitution^116^ and via the solubility-enhancing negatively charged nature of the ribosomal surface.^30,59,117^

The present work highlights an additional feature of the ribosome by showing that the L23 ribosomal protein bears a highly nonpolar region surrounded by negatively charged residues (Figure 4d and refs (77) and (117)) that interact with apoMb RNCs (Figure 4e). Therefore, we propose that the highly conserved portion of the outer surface of the ribosome comprising the L23 nonpolar cavity may promote nascent chain solubility and effectively serve as a molecular chaperone. While further studies are necessary to test this concept in the context of protein folding and aggregation assays, this work sets the stage for these future experiments by identifying a distinct nascent chain interaction site.

It has recently been shown that proteins bearing nonpolar and negatively charged regions are less likely to aggregate than proteins bearing nonpolar and positively charged side-chain regions,^118^ explaining why chaperone-binding motifs of client proteins often include nonpolar and positively charged regions.^118^ apoMb bears two major and three minor such “chaperone-friendly” regions,^119^ rendering it a good candidate to interact with L23 while ribosome-bound. Conversely, to maximize nonpolar–nonpolar and positive–negative electrostatic interactions, chaperones including TF and Hsp70 bear client protein binding regions containing negatively charged and nonpolar regions. In the case of the ribosomal outer surface, nonpolar patches close to the exit tunnel are relatively rare (yet, see L23’s and, to a lesser extent, L29’s nonpolar regions in Figure 4), while all rRNAs (due to the phosphate groups) and all ribosomal proteins (due to their charge segregation^117^) bear a highly negatively charged outer surface. These features render the ribosome intrinsically fit to serve as a molecular chaperone candidate.

Interestingly, the L23 r-protein underwent evolution late in time relative to other ribosomal proteins.^120^ Thus, given that molecular chaperones tend to accelerate evolution,^121^ the L23 r-protein may have played an evolutionary role in the promotion of chaperone-assisted protein folding.^122−124^ The high level of conservation of the L23 nonpolar residues interacting with the nascent chain, shown in Figure 5e, supports this concept.

## Conclusions and Outlook

The main findings of this work can be summarized as follows. First, as schematically shown in Figure 7a–c, apoMb RNCs interact extensively with both r-proteins and rRNA. Importantly, these interactions encompass not only the ribosomal tunnel core and vestibule, as previously believed, but also the outer ribosomal surface close to the tunnel exit. Second, nascent chains of a medium-size single-domain protein (ca. 150 residues) like apoMb take advantage of both interactions with the ribosomal surface (including the L23 nonpolar region) and independent conformational sampling of the N-terminal subdomain. Therefore, within this single-domain size regime, protein birth seems to be the result of an intriguing combination of ribosome-mediated assistance and independent conformational sampling of the N-terminal regions.

Despite the progress achieved to date, there is still a compelling need to elucidate nascent chain conformation and dynamics to an even greater extent than what has been possible so far, including the present work. The recent availability of multiple appropriate technical tools and the many viable target nascent proteins raise our hopes that this lofty goal will soon be attained.

## Materials and Methods

### Generation of Fluorophore-Labeled Ribosome-Bound Nascent Chains (RNCs)

RNCs of full-length sperm whale apomyoglobin (apoMb) were generated via a transcription–translation *E. coli* cell-free system based on an S30 extract (*Δtig* A19 cell strain, lacking the trigger factor gene) generated in house as described.^56^ Hsp70 chaperone activity was suppressed via the KLR-70 inhibitory peptide^57^ to a final concentration of 0.2 mM. The BODIPY-FL-Met-tRNA^fMet^ fluorophore-labeled charged tRNA, prepared as described,^56,60^ was added to the cell-free mixture to generate RNCs bearing a site-specifically incorporated BODIPY-FL fluorophore covalently linked to the amino group of the RNC’s N-terminal methionine. The cell-free mixture contained a pET-Blue1 plasmid (0.03 μg/μL) encoding the *E. coli* codon-optimized sperm whale apoMb gene,^74^ BODIPY-FL-Met-tRNA^fMet^ (1.1 μM), antisense oligonucleotides (0.15 μg/μL, see Supporting Information) targeting ribosome stalling immediately before the apoMb-gene stop codon via RNase H-mediated oligodeoxynucleotide-directed mRNA cleavage,^6,60,125,126^ and an anti-ssrA oligonucleotide (0.15 μg/μL) to prevent ribosomal rescue of stalled nascent chains.^56,127^ Briefly, the preassembled cell-free transcription-translation mixture was incubated at 37 ^ο^C for 30 min, followed by quenching upon further incubation on ice for 15 min. RNCs were then loaded onto a sucrose cushion (1.1 M sucrose, 20 mM tris base, 10 mM Mg(OAc)_2_, 500 mM NH_4_Cl, and 0.5 mM EDTA, pH 7.0) and then pelleted down by centrifugation at 160,000 g for 1 h at 4 ^ο^C.^56^ The RNC pellet was then redissolved in resuspension buffer (10 mM Tris-HCl, 10 mM Mg(OAc)_2_, 60 mM NH_4_Cl, 0.5 mM EDTA, and 1.0 mM DTT, pH 7.0) by shaking at 250 rpm for 1 h on ice.

### Generation of Cross-Linked RNCs

Resuspended RNCs, generated as described in the previous section, were aliquoted and incubated on ice until they were ready for use. An 800 mM stock solution of the EDC cross-linker (1-ethyl-3-(3-(dimethylamino)propyl)carbodiimide hydrochloride, Thermo Fisher Scientific) was then freshly prepared in DNase/RNase free water (Corning) followed by pH adjustment to 6.8–7.0 via the addition of 1.0 M KOH. Resuspended RNCs were then incubated with the EDC cross-linker at a final concentration of 80 mM at 30 °C for 30 min. The cross-linking reaction was then quenched via the addition of quenching buffer (1 M Tris-HCl, pH 7, 1 M glycine, and 1 M KOAc) at a ratio of 0.74 μL every 6 μL of resuspended RNCs. Cross-linked RNCs were then flash-frozen in liquid nitrogen and stored at −80 °C.

### Grid Preparation for Cryo-EM Data Collection

The grids employed for single-particle cryo-EM (sp-cryo-EM) data collection were cleaned as follows. Multiple UltrAuFoil 300 mesh holey carbon R2/2 (Quantifoil) grids were placed on filter paper. Chloroform was then gently deposited onto the grids and allowed to dry overnight in a fume hood. The next day, the grids were rinsed with HPLC-grade isopropyl alcohol (Thermo Fisher Scientific), followed by HPLC-grade acetone (Thermo Fisher Scientific), and allowed to air-dry. Any extra grids not to be used immediately were stored in a desiccator at ambient temperature.

The grids to be used for sp-cryo-EM data collection were coated with a graphene oxide film according to the following procedure. A graphene oxide solution (0.002 mg/mL in water, Thermo Fisher Scientific) was spun at 350 °C for 30 s. While not disturbing the graphene oxide pellet, 80% of the initial graphene oxide solution was removed, and the pellet was resuspended. Grids were then glow-discharged by using a Glo Cube (Quorum Technologies) at 20 mA for 1 min. At this stage, 3 μL of graphene oxide solution was placed onto each grid, followed by cleaning with fresh RNase-free water (Corning). Grids were allowed to air-dry in their grid box, which was stored in a desiccator at ambient temperature.

After being coated with graphene oxide (see previous section), blank grids were loaded into a Vitrobot (Thermo Fisher Scientific) for plunging and freezing. After loading onto the Vitrobot, 5 μL of the resuspended cross-linked apoMb RNC solution (thawed on ice) was placed onto the graphene coated side of grids and set to a waiting time of 30 s. Grids were then blotted for 12 s using Electron Microscopy Sciences TM Filter Paper grade 595, 50 mm (Thermo Fisher Scientific), and plunged at a force (a.u.) of −10 at 100% humidity. Grids were then stored in liquid nitrogen until they were ready for screening.

### sp-Cryo-EM Data Collection and Processing

sp-Cryo-EM movies on cross-linked resuspended apoMb RNCs were collected at the S^2^C^2^ Stanford-SLAC Cryo-EM facility and screened on site to assess grid quality (assessing particle concentration, orientation, and film quality were adequate for data collection) on a Titan Krios (Thermo Fisher Scientific) electron microscope operating at 300 keV and equipped with a K3 detector and an objective aperture of 100 μm, resulting in a pixel size of 0.946 Å. Data were then collected with the EPU software (Thermo Fisher Scientific) with a defocus range of 1.5–2.1 μm and a total electron dose of 50 e^–^/Å^2^/frame. Movie frames were aligned in 5 × 5 patches and dose weighted via the MotionCor2 software.^128^ The 9112 movies acquired according to the above procedure were uploaded onto the CryoSPARC (v 3.8–4.2) image processing software.^75^ All movies underwent initial patch-motion correction and patch contrast transfer function (CTF) estimation. As a result of this process, 1 487 887 particles were extracted and underwent 2D classification. A total of 821 587 particles were chosen. Additional particle picking was done via ab initio reconstruction after particles were sorted into four clusters. The cluster that resembled 70S ribosomes, containing 397 467 particles, underwent another round of the same ab initio reconstruction, leading to a final choice of two clusters containing 348,521 particles. A final ab initio reconstruction procedure was employed to generate an initial model including all 348 521 particles. A subsequent round of 3D variability analysis, eliminating particles lacking p-site tRNA, resulted in 213 770 particles that underwent nonuniform refinement, which resulted in an average resolution of 2.91 Å with a 0.143 Fourier shell correlation (FSC). Local resolution was estimated via the CryoSPARC local resolution job by importing the mask and volume from the particles (see Supporting Information). The final cryo-EM density map was obtained via the RELION (v 2.1) software^76^ with a 4 Å low-pass filter to enable identification of nascent chain density while maintaining a reasonable 70S ribosome resolution. Finally, 3D structural-model building and refinement were carried out as follows. The initial *E. coli* 70S ribosome structure (PDBID:7k00^129^) was uploaded onto the ChimeraX software,^130^ and individual r-proteins and rRNA were fit via the ridged-body routine. The nascent chain structure and corresponding poly-Ala loops were built with Coot (v 0.9).^131^ Phenix (v 1.18^132^) was used to validate the final model and to assess the quality of the fit. Density-fit analysis, rotamer analysis, and Ramachandran plots are available in the Supporting Information.

### Amino Acid Conservation Analysis of the L23 Ribosomal Protein

Conservation analysis of the L23 r-protein was carried out with the Clustal Omega^133^ and visualized with the WebLogo (v 2.8.2)^134,135^ software packages. Sequence comparisons and matching graphical representations of amino acid conservation patterns across a variety of bacteria were generated from the Database of Essential Genes (DEG).^99,100^ All amino acid sequences were deduced from Uniprot. First, the amino acid sequence of *E. coli* L23 was compared to the sequences of the same protein from all of the other bacteria (within the DEG database) carrying L23 as an essential protein. This analysis included the following organisms (Uniprot-ID listed in parentheses): *Rhizorhabdus wittichii* (A5 V602), *Mycoplasma genitalium* (P47399), *Porphyromonas gingivalis* (B2RLZ0), *Bacteroides fragilis* (E1WPD1), *Bacteroides thetaiotaomicron* (Q8A478), *Campylobacter jejuni* (Q0P7S6), *Helicobacter pylori* (P66119), *Francisella tularensis* (A0A6N3JFX8), *Caulobacter vibrioides* (B8H4D6*), Acinetobacter baylyi* (Q6F7R4), *Burkholderia pseudomallei* (Q63Q13), *Burkholderia thailandensis* (Q2SU29), *Pseudomonas aeruginosa* (Q9HWD7), *Haemophilus influenzae* (P44361), *Vibrio cholerae* (A0A085SZR1), *E. coli* (P0ADZ0), *Salmonella typhi* (Q8XGM6), *Mycobacterium tuberculosis* (P9WHB9), *Streptococcus sanguinis* (A3CK65), *Bacillus subtilis* (P42924), *Staphylococcus aureus* (Q7A459), *Mycoplasma pulmonis* (Q98PY3), *Shewanella oneidensis* (Q8EK66), *Synechococcus elongatus* (Q31L09), *Acinetobacter baumannii* (B7IA37), *Agrobacterium fabrum* (Q8UE20), *Fransicella tularensis SCHU* (Q5NHW6), *Burkholderia cenocepacia* (B4E5C2), and *Providencia stuartii* (A0A1L311Y2). Second, the same *E. coli* strain was compared to the bacteria (in the DEG database) that carry L23 as a nonessential gene. These bacteria include the following: *Rhodopseudomonas palustris* (Q6N4T7), *Streptococcus agalactiae* (R4Z804), *Brevundimonas subvibrioides* (A0A258HC12), *Bacillus thuringiensis* (A0R8I2), *Streptococcus mutans* (A0A2J9QGG4), *Neisseria gonorrheae* (Q5F5S9), *Ralstonia nicotianae* (Q8XV14), *Streptococcus suis* (A4VSF6), *Mycobacterium avium* (A0QL16), *Streptococcus pneumoniae* (C1CC08), and *Streptococcus pyogenes* (Q9A1 × 2). Note that while the analysis reported here is limited to the bacteria in the DEG database, more comprehensive comparisons across thousands of bacteria could also be carried out; however, these comparisons are beyond the scope of this study.

### Fluorescence Lifetimes and Anisotropy Decays

Fluorescence lifetime and anisotropy decay experiments were carried out on a Chronos spectrofluorometer (ISS Inc.), which operates in the frequency domain.^102^ Data were collected upon excitation with a laser diode operating at 477 nm. A 480 nm band-pass excitation filter and a 495 nm long-pass emission filter (HHQ495lp, Chroma) were used, respectively. The excitation polarizer was set to a vertical position in both lifetime and anisotropy decay experiments. The emission polarizer was rotated between vertical and horizontal positions as customary in anisotropy experiments, while it was set to a fixed angle of 54.7° for lifetime experiments. The fluorometer temperature of the compartment surrounding the cuvette was maintained at 25 °C via a circulating water bath. Samples were temperature-equilibrated upon incubation in the cuvette inside the spectrometer for at least 30 min before data collection. Fluorescence anisotropy data were corrected upon measuring the G-factor on each experimental day as described.^136^ Fluorescence lifetime and anisotropy-decay data were fit with the Globals Software Suite (LFD), and χ^2^ values were assessed for each fit upon assuming instrumental errors for the modulation and phase of 0.004 and 0.2°, respectively.^103^ Fluorescence lifetime data were fit to three-component exponential decay expressions. The first two discrete components yielded fluorescence lifetimes, while the third component was set to a fixed fictitious lifetime value of 0.001 ns to account for small potential extents of light scattering. Fluorescence anisotropy decay data on both non-cross-linked and EDC-cross-linked RNCs were fit to three exponential-decay expressions, given that twofold or greater decreases in reduced χ^2^ were observed upon comparing the results to two-component fits. Finally, order parameters and cone semiangles were determined from anisotropy decay pre-exponential factors as described.^43^

## Supporting Information Available

Summary of cryo-EM data collection and processing parameters, cryo-EM local-resolution map of apoMb RNCs, histogram and directional FSC plot (CryoSPARC) of apoMb RNCs, Ramachandran plots, secondary-structure maps of apoMb RNCs, and uncropped gel images (PDF)
Transparent Peer Review report available (PDF)

## References

[ref1] Bhatia S.; Udgaonkar J. B. Heterogeneity in Protein Folding and Unfolding Reactions. Chem. Rev. 2022, 122 (9), 8911–8935. 10.1021/acs.chemrev.1c00704. 35275612

[ref2] Chilom C. G.; Popescu A. I. Biophysics of Protein Folding. A Short Review. Roman. Rep. Phys. 2020, 72 (3), 604.

[ref3] Zhang H.; Gong W. B.; Wu S.; Perrett S. Studying Protein Folding in Health and Disease Using Biophysical Approaches. Emerg. Top. Life Sci. 2021, 5 (1), 29–38. 10.1042/ETLS20200317. 33660767 PMC8138949

[ref4] Gruebele M.; Sabelko J.; Ballew R.; Ervin J. Laser Temperature Jump Induced Protein Refolding. Acc. Chem. Res. 1998, 31 (11), 699–707. 10.1021/ar970083x.

[ref5] Sosnick T. R.; Barrick D. The Folding of Single Domain Proteins - Have We Reached a Consensus?. Curr. Opin. Struct. Biol. 2011, 21 (1), 12–24. 10.1016/j.sbi.2010.11.002. 21144739 PMC3039110

[ref6] Fedyukina D. V.; Cavagnero S. Protein Folding at the Exit Tunnel. Annu. Rev. Biophys. 2011, 40, 337–359. 10.1146/annurev-biophys-042910-155338. 21370971 PMC5807062

[ref7] Gruebele M.; Dave K.; Sukenik S. Globular Protein Folding in Vitro and in Vivo. In Annu. Rev. Biophys., Dill K. A., Ed.; Annual Reviews of Biophysics, Vol. 45; 2016; 233–251. 10.1146/annurev-biophys-062215-011236 27391927

[ref8] Gershenson A.; Gierasch L. M. Protein Folding in the Cell: Challenges and Progress. Curr. Opin. Struct. Biol. 2011, 21 (1), 32–41. 10.1016/j.sbi.2010.11.001. 21112769 PMC3072030

[ref9] Jaenicke R. Protein Folding and Association: In Vitro Studies for Self-Organization and Targeting in the Cell. Curr. Top. Cell. Regul. 1996, 34, 209–314. 10.1016/S0070-2137(96)80008-2. 8646849

[ref10] Cascella R.; Bigi A.; Cremades N.; Cecchi C. Effects of Oligomer Toxicity, Fibril Toxicity and Fibril Spreading in Synucleinopathies. Cell. Mol. Life Sci. 2022, 79 (3), 174 10.1007/s00018-022-04166-9. 35244787 PMC8897347

[ref11] Englander S. W.; Mayne L.; Krishna M. M. G. Protein Folding and Misfolding: Mechanism and Principles. Q. Rev. Biophys. 2007, 40 (4), 287–326. 10.1017/S0033583508004654. 18405419 PMC3433742

[ref12] Bohnsack K. E.; Bohnsack M. T. Uncovering the Assembly Pathway of Human Ribosomes and Its Emerging Links to Disease. EMBO J. 2019, 38 (13), e100278 10.15252/embj.2018100278. 31268599 PMC6600647

[ref13] Steitz T. A.; Moore P. B. Rna, the First Macromolecular Catalyst: The Ribosome Is a Ribozyme. Trends Biochem. Sci. 2003, 28 (8), 411–418. 10.1016/S0968-0004(03)00169-5. 12932729

[ref14] Liutkute M.; Samatova E.; Rodnina M. V. Cotranslational Folding of Proteins on the Ribosome. Biomol. 2020, 10 (1), 97 10.3390/biom10010097. PMC7023365 31936054

[ref15] Ciryam P.; Morimoto R. I.; Vendruscolo M.; Dobson C. M.; O’Brien E. P. In Vivo Translation Rates Can Substantially Delay the Cotranslational Folding of the Escherichia Coli Cytosolic Proteome. Proc. Natl. Acad. Sci. U.S.A. 2013, 110 (2), E132–E140. 10.1073/pnas.1213624110. 23256155 PMC3545769

[ref16] Gilbert R. J. C.; Fucini P.; Connell S.; Fuller S. D.; Nierhaus K. H.; Robinson C. V.; Dobson C. M.; Stuart D. I. Three-Dimensional Structures of Translating Ribosomes by Cryo-Em. Mol. Cell 2004, 14 (1), 57–66. 10.1016/S1097-2765(04)00163-7. 15068803

[ref17] Frank J.; Penczek P.; Grassucci R. A.; Heagle A.; Spahn C. M. T.; Agrawal R. K. Cryo-Electron Microscopy of the Translational Apparatus: Experimental Evidence for the Paths of Mrna, Trna, and the Polypeptide Chain. In The Ribosome: Structure, Function, Antibiotics, and Cellular Interactions; ASM Press: Washington, D.C., 2000; pp 45–51.

[ref18] Voss N. R.; Gerstein M.; Steitz T. A.; Moore P. B. The Geometry of the Ribosomal Polypeptide Exit Tunnel. J. Mol. Biol. 2006, 360 (4), 893–906. 10.1016/j.jmb.2006.05.023. 16784753

[ref19] Kramer G.; Ramachandiran V.; Hardesty B. Cotranslational Folding—Omnia Mea Mecum Porto?. Int. J. Biochem. Cell Biol. 2001, 33 (6), 541–553. 10.1016/S1357-2725(01)00044-9. 11378437

[ref20] Malkin L. I.; Rich A. Partial Resistance of Nascent Polypeptide Chains to Proteolytic Digestion Due to Ribosomal Shielding. J. Mol. Biol. 1967, 26 (2), 329–346. 10.1016/0022-2836(67)90301-4. 4962271

[ref21] Tsalkova T.; Odom O.; Kramer G.; Hardesty B. Different Conformations of Nascent Peptides on Ribosomes. J. Mol. Biol. 1998, 278 (4), 713–723. 10.1006/jmbi.1998.1721. 9614937

[ref22] Woolhead C. A.; McCormick P. J.; Johnson A. E. Nascent Membrane and Secretory Proteins Differ in Fret-Detected Folding Far inside the Ribosome and in Their Exposure to Ribosomal Proteins. Cell 2004, 116 (5), 725–736. 10.1016/S0092-8674(04)00169-2. 15006354

[ref23] Ziv G.; Haran G.; Thirumalai D. Ribosome Exit Tunnel Can Entropically Stabilize Α-Helices. Proc. Natl. Acad. Sci. U.S.A. 2005, 102 (52), 18956 10.1073/pnas.0508234102. 16357202 PMC1323178

[ref24] Agirrezabala X.; Samatova E.; Macher M.; Liutkute M.; Maiti M.; Gil-Carton D.; Novacek J.; Valle M.; Rodnina M. V. A Switch from Α-Helical to Β-Strand Conformation During Co-Translational Protein Folding. EMBO J. 2022, 41 (4), e109175 10.15252/embj.2021109175. 34994471 PMC8844987

[ref25] Bañó-Polo M.; Baeza-Delgado C.; Tamborero S.; Hazel A.; Grau B.; Nilsson I.; Whitley P.; Gumbart J. C.; von Heijne G.; Mingarro I. Transmembrane but Not Soluble Helices Fold inside the Ribosome Tunnel. Nat. Commun. 2018, 9, 5246 10.1038/s41467-018-07554-7. 30531789 PMC6286305

[ref26] Bhushan S.; Gartmann M.; Halic M.; Armache J.-P.; Jarasch A.; Mielke T.; Berninghausen O.; Wilson D. N.; Beckmann R. Α-Helical Nascent Polypeptide Chains Visualized within Distinct Regions of the Ribosomal Exit Tunnel. Nat. Struct. Mol. Biol. 2010, 17 (3), 313–317. 10.1038/nsmb.1756. 20139981

[ref27] Wilson D. N.; Beckmann R. The Ribosomal Tunnel as a Functional Environment for Nascent Polypeptide Folding and Translational Stalling. Curr. Opin. Struct. Biol. 2011, 21 (2), 274–282. 10.1016/j.sbi.2011.01.007. 21316217

[ref28] Nilsson O. B.; Hedman R.; Marino J.; Wickles S.; Bischoff L.; Johansson M.; Müller-Lucks A.; Trovato F.; Puglisi J. D.; O’Brien E. P.; et al. Cotranslational Protein Folding inside the Ribosome Exit Tunnel. Cell Rep. 2015, 12 (10), 1533–1540. 10.1016/j.celrep.2015.07.065. 26321634 PMC4571824

[ref29] Nilsson O. B.; Hedman R.; Marino J.; Wickles S.; Bischoff L.; Johansson M.; Muller-Lucks A.; Trovato F.; Puglisi J. D.; O’Brien E. P.; et al. Cotranslational Protein Folding inside the Ribosome Exit Tunnel. Cell Rep. 2015, 12 (10), 1533–1540. 10.1016/j.celrep.2015.07.065. 26321634 PMC4571824

[ref30] Addabbo R. M.; Hutchinson R. B.; Allaman H. J.; Dalphin M. D.; Mecha M. F.; Liu Y.; Staikos A.; Cavagnero S. Critical Beginnings: Selective Tuning of Solubility and Structural Accuracy of Newly Synthesized Proteins by the Hsp70 Chaperone System. J. Phys. Chem. B 2023, 127, 3990–4014. 10.1021/acs.jpcb.2c08485. 37130318 PMC10829761

[ref31] Masse M. M.; Guzman-Luna V.; Varela A. E.; Hutchinson R. B.; Srivastava A.; Wei W.; Fuchs A. M.; Cavagnero S. Nascent-Chain Interaction Networks and Their Effect on the Bacterial Ribosome. bioRxiv 2022, 2022.2010.2031.514555 10.1101/2022.10.31.514555.

[ref32] Hutchinson R. B.; Chen X.; Zhou N. K.; Cavagnero S. Fluorescence Anisotropy Decays and Microscale-Volume Viscometry Reveal the Compaction of Ribosome-Bound Nascent Proteins. J. Phys. Chem. B 2021, 125 (24), 6543–6558. 10.1021/acs.jpcb.1c04473. 34110829 PMC8741338

[ref33] Agirrezabala X.; Samatova E.; Macher M.; Liutkute M.; Maiti M.; Gil-Carton D.; Novacek J.; Valle M.; Rodnina M. V. A Switch from Α-Helical to Β-Strand Conformation During Co-Translational Protein Folding. EMBO J. 2022, 41 (4), e109175 10.15252/embj.2021109175. 34994471 PMC8844987

[ref34] Ahn M.; Wlodarski T.; Mitropoulou A.; Chan S. H. S.; Sidhu H.; Plessa E.; Becker T. A.; Budisa N.; Waudby C. A.; Beckmann R. Modulating Co-Translational Protein Folding by Rational Design and Ribosome Engineering. Nat. Commun. 2022, 13, 4243 10.1038/s41467-022-31906-z. 35869078 PMC9307626

[ref35] Tian P.; Steward A.; Kudva R.; Su T.; Shilling P. J.; Nickson A. A.; Hollins J. J.; Beckmann R.; Von Heijne G.; Clarke J.; et al. Folding Pathway of an Ig Domain Is Conserved on and Off the Ribosome. Proc. Natl. Acad. Sci. U.S.A. 2018, 115 (48), E11284–E11293. 10.1073/pnas.1810523115. 30413621 PMC6275497

[ref36] Nilsson O. B.; Nickson A. A.; Hollins J. J.; Wickles S.; Steward A.; Beckmann R.; von Heijne G.; Clarke J. Cotranslational Folding of Spectrin Domains Via Partially Structured States. Nat. Struct. Mol. Biol. 2017, 24 (3), 221–225. 10.1038/nsmb.3355. 28112730

[ref37] Addabbo R. M.; Dalphin M. D.; Mecha M. F.; Liu Y.; Staikos A.; Guzman-Luna V.; Cavagnero S. Complementary Role of Co-and Post-Translational Events in De Novo Protein Biogenesis. J. Phys. Chem. B 2020, 124 (30), 6488–6507. 10.1021/acs.jpcb.0c03039. 32456434

[ref38] Samelson A. J.; Jensen M. K.; Soto R. A.; Cate J. H.; Marqusee S. Quantitative Determination of Ribosome Nascent Chain Stability. Proc. Natl. Acad. Sci. U.S.A. 2016, 113 (47), 13402–13407. 10.1073/pnas.1610272113. 27821780 PMC5127326

[ref39] Wruck F.; Tian P. F.; Kudva R.; Best R. B.; von Heijne G.; Tans S. J.; Katranidis A. The Ribosome Modulates Folding inside the Ribosomal Exit Tunnel. Commun. Biol. 2021, 4, 523 10.1038/s42003-021-02055-8. 33953328 PMC8100117

[ref40] Ellis J. P.; Bakke C. K.; Kirchdoerfer R. N.; Jungbauer L. M.; Cavagnero S. Chain Dynamics of Nascent Polypeptides Emerging from the Ribosome. ACS Chem. Biol. 2008, 3 (9), 555–566. 10.1021/cb800059u. 18717565 PMC2572860

[ref41] Ellis J. P.; Culviner P. H.; Cavagnero S. Confined Dynamics of a Ribosome-Bound Nascent Globin: Cone Angle Analysis of Fluorescence Depolarization Decays in the Presence of Two Local Motions. Protein Sci. 2009, 18 (10), 2003–2015. 10.1002/pro.196. 19569194 PMC2786964

[ref42] Hutchinson R. B.; Chen X.; Zhou N.; Cavagnero S. Fluorescence Anisotropy Decays and Microscale-Volume Viscometry Reveal the Compaction of Ribosome-Bound Nascent Proteins. J. Phys. Chem. B 2021, 125 (24), 6543–6558. 10.1021/acs.jpcb.1c04473. 34110829 PMC8741338

[ref43] Ellis J. P.; Culviner P. H.; Cavagnero S. Confined Dynamics of a Ribosome-Bound Nascent Globin: Cone Angle Analysis of Fluorescence Depolarization Decays in the Presence of Two Local Motions. Protein Sci. 2009, 18 (10), 2003–2015. 10.1002/pro.196. 19569194 PMC2786964

[ref44] Knight A. M.; Culviner P. H.; Kurt-Yilmaz N. e.; Zou T.; Ozkan S. B.; Cavagnero S. Electrostatic Effect of the Ribosomal Surface on Nascent Polypeptide Dynamics. ACS Chem. Biol. 2013, 8 (6), 1195–1204. 10.1021/cb400030n. 23517476

[ref45] Guzman-Luna V.; Fuchs A. M.; Allen A. J.; Staikos A.; Cavagnero S. An Intrinsically Disordered Nascent Protein Interacts with Specific Regions of the Ribosomal Surface near the Exit Tunnel. Commun. Biol. 2021, 4, 1236 10.1038/s42003-021-02752-4. 34716402 PMC8556260

[ref46] Weinreis S. A.; Ellis J. P.; Cavagnero S. Dynamic Fluorescence Depolarization: A Powerful Tool to Explore Protein Folding on the Ribosome. Methods 2010, 52 (1), 57–73. 10.1016/j.ymeth.2010.06.001. 20685617 PMC2934862

[ref47] Samatova E.; Daberger J.; Liutkute M.; Rodnina M. V. Translational Control by Ribosome Pausing in Bacteria: How a Non-Uniform Pace of Translation Affects Protein Production and Folding. Front. Microbiol. 2021, 11, 619430 10.3389/fmicb.2020.619430. 33505387 PMC7829197

[ref48] Lu J.; Deutsch C. Electrostatics in the Ribosomal Tunnel Modulate Chain Elongation Rates. J. Mol. Biol. 2008, 384 (1), 73–86. 10.1016/j.jmb.2008.08.089. 18822297 PMC2655213

[ref49] Charneski C. A.; Hurst L. D. Positively Charged Residues Are the Major Determinants of Ribosomal Velocity. PLoS Biol. 2013, 11 (3), e1001508 10.1371/journal.pbio.1001508. 23554576 PMC3595205

[ref50] Requião R. D.; de Souza H. J. A.; Rossetto S.; Domitrovic T.; Palhano F. L. Increased Ribosome Density Associated to Positively Charged Residues Is Evident in Ribosome Profiling Experiments Performed in the Absence of Translation Inhibitors. RNA Biol. 2016, 13 (6), 561–568. 10.1080/15476286.2016.1172755. 27064519 PMC4962802

[ref51] Lu J. L.; Kobertz W. R.; Deutsch C. Mapping the Electrostatic Potential within the Ribosomal Exit Tunnel. J. Mol. Biol. 2007, 371 (5), 1378–1391. 10.1016/j.jmb.2007.06.038. 17631312

[ref52] Seidelt B.; Innis C. A.; Wilson D. N.; Gartmann M.; Armache J.-P.; Villa E.; Trabuco L. G.; Becker T.; Mielke T.; Schulten K.; et al. Structural Insight into Nascent Polypeptide Chain–Mediated Translational Stalling. Science 2009, 326 (5958), 1412–1415. 10.1126/science.1177662. 19933110 PMC2920484

[ref53] Cruz-Vera L. R.; Rajagopal S.; Squires C.; Yanofsky C. Features of Ribosome-Peptidyl-Trna Interactions Essential for Tryptophan Induction of Tna Operon Expression. Mol. Cell 2005, 19 (3), 333–343. 10.1016/j.molcel.2005.06.013. 16061180

[ref54] Bhushan S.; Hoffmann T.; Seidelt B.; Frauenfeld J.; Mielke T.; Berninghausen O.; Wilson D. N.; Beckmann R. Secm-Stalled Ribosomes Adopt an Altered Geometry at the Peptidyl Transferase Center. PLoS Biol. 2011, 9 (1), e1000581 10.1371/journal.pbio.1000581. 21267063 PMC3022528

[ref55] Su T.; Cheng J.; Sohmen D.; Hedman R.; Berninghausen O.; von Heijne G.; Wilson D. N.; Beckmann R. The Force-Sensing Peptide Vemp Employs Extreme Compaction and Secondary Structure Formation to Induce Ribosomal Stalling. eLife 2017, 6, e25642 10.7554/eLife.25642. 28556777 PMC5449182

[ref56] Bakke C. K.; Jungbauer L. M.; Cavagnero S. In Vitro Expression and Characterization of Native Apomyoglobin under Low Molecular Crowding Conditions. Prot. Expr. Purif. 2006, 45 (2), 381–392. 10.1016/j.pep.2005.08.001. 16169747

[ref57] Dalphin M. D.; Stangl A. J.; Liu Y.; Cavagnero S. Klr-70: A Novel Cationic Inhibitor of the Bacterial Hsp70 Chaperone. Biochemistry 2020, 59, 1946–1960. 10.1021/acs.biochem.0c00320. 32326704 PMC8322834

[ref58] Murata K.; Wolf M. Cryo-Electron Microscopy for Structural Analysis of Dynamic Biological Macromolecules. Biochim. Biophys. Acta Gen. Subj. 2018, 1862 (2), 324–334. 10.1016/j.bbagen.2017.07.020. 28756276

[ref59] Addabbo R. M.; Dalphin M. D.; Mecha M. F.; Liu Y.; Staikos A.; Guzman-Luna V.; Cavagnero S. Complementary Role of Co- and Post-Translational Events in De Novo Protein Biogenesis. J. Phys. Chem. B 2020, 124 (30), 6488–6507. 10.1021/acs.jpcb.0c03039. 32456434

[ref60] Ellis J. P.; Bakke C. K.; Kirchdoerfer R. N.; Jungbauer L. M.; Cavagnero S. Chain Dynamics of Nascent Polypeptides Emerging from the Ribosome. ACS Chem. Biol. 2008, 3 (9), 555–566. 10.1021/cb800059u. 18717565 PMC2572860

[ref61] Hoare D. t.; Koshland D. A Method for the Quantitative Modification and Estimation of Carboxylic Acid Groups in Proteins. J. Biol. Chem. 1967, 242 (10), 2447–2453. 10.1016/S0021-9258(18)95981-8. 6026234

[ref62] Hermanson G. Bioconjugate Techniques, 3rd ed.; Elsevier, 2013.

[ref63] Carragher B.; Cheng Y.; Frost A.; Glaeser R. M.; Lander G. C.; Nogales E.; Wang W. Current Outcomes When Optimizing ‘Standard’ Sample Preparation for Single-Particle Cryo-Em. J. Microsc. 2019, 276 (1), 39–45. 10.1111/jmi.12834. 31553060 PMC7050573

[ref64] Ishihama Y.; Schmidt T.; Rappsilber J.; Mann M.; Hartl F. U.; Kerner M. J.; Frishman D. Protein Abundance Profiling of the *Escherichia Coli* Cytosol. BMC Genomics 2008, 9, 102 10.1186/1471-2164-9-102. 18304323 PMC2292177

[ref65] Lecomte J. T. J.; Sukits S. F.; Bhattacharya S.; Falzone C. J. Conformational Properties of Native Sperm Whale Apomyoglobin in Solution. Protein Sci. 1999, 8 (7), 1484–1491. 10.1110/ps.8.7.1484. 10422837 PMC2144374

[ref66] Lecomte J. T. J.; Kao Y. H.; Cocco M. J. The Native State of Apomyoglobin Described by Proton Nmr Spectroscopy: The a-B-G-H Interface of Wild-Type Sperm Whale Apomyoglobin. Proteins 1996, 25 (3), 267–285. 10.1002/(SICI)1097-0134(199607)25:3<267::AID-PROT1>3.0.CO;2-D. 8844864

[ref67] Cocco M. J.; Lecomte J. T. J. The Native-State of Apomyoglobin Described by Proton Nmr-Spectroscopy - Interaction with the Paramagnetic Probe Hytempo and the Fluorescencent Dye Ans. Protein Sci. 1994, 3 (2), 267–281. 10.1002/pro.5560030211. 8003963 PMC2142796

[ref68] Eliezer D.; Wright P. E. Is Apomyoglobin a Molten Globule? Structural Characterization by Nmr. J. Mol. Biol. 1996, 263 (4), 531–538. 10.1006/jmbi.1996.0596. 8918936

[ref69] Dyson H. J.; Wright P. E. How Does Your Protein Fold? Elucidating the Apomyoglobin Folding Pathway. Acc. Chem. Res. 2017, 50 (1), 105–111. 10.1021/acs.accounts.6b00511. 28032989 PMC5241236

[ref70] Cavagnero S.; Nishimura C.; Schwarzinger S.; Dyson H. J.; Wright P. E. Conformation and Dynamic Characterization of the Molten Globule State of an Apomyoglobin Mutant with an Altered Folding Pathway. Biochemistry 2001, 40, 14459–14467. 10.1021/bi011500n. 11724558

[ref71] Garcia C.; Nishimura C.; Cavagnero S.; Dyson H. J.; Wright P. E. Changes in the Apomyoglobin Folding Pathway Caused by Mutation of the Distal Histidine Residue. Biochemistry 2000, 39, 11227–11237. 10.1021/bi0010266. 10985768

[ref72] Cavagnero S.; Dyson H. J.; Wright P. E. Effect of H Helix Destabilizing Mutations on the Kinetic and Equilibrium Folding of Apomyoglobin. J. Mol. Biol. 1999, 285 (1), 269–282. 10.1006/jmbi.1998.2273. 9878405

[ref73] Tsui V.; Garcia C.; Cavagnero S.; Siuzdak G.; Dyson H. J.; Wright P. E. Quench-Flow Experiments Combined with Mass Spectrometry Show Apomyoglobin Folds through an Obligatory Intermediate. Protein Sci. 1999, 8, 45–49. 10.1110/ps.8.1.45. 10210182 PMC2144105

[ref74] Springer B. A.; Sligar S. G. High-Level Expression of Sperm Whale Myoglobin in *Escherichia Coli*. Proc. Natl. Acad. Sci. U.S.A. 1987, 84 (24), 8961–8965. 10.1073/pnas.84.24.8961. 3321062 PMC299671

[ref75] Punjani A.; Rubinstein J. L.; Fleet D. J.; Brubaker M. A. Cryosparc: Algorithms for Rapid Unsupervised Cryo-Em Structure Determination. Nat. Methods 2017, 14 (3), 290–296. 10.1038/nmeth.4169. 28165473

[ref76] Scheres S. H. Relion: Implementation of a Bayesian Approach to Cryo-Em Structure Determination. J. Struct. Biol. 2012, 180 (3), 519–530. 10.1016/j.jsb.2012.09.006. 23000701 PMC3690530

[ref77] Guzman-Luna V.; Fuchs A. M.; Allen A. J.; Staikos A.; Cavagnero S. An Intrinsically Disordered Nascent Protein Interacts with Specific Regions of the Ribosomal Surface near the Exit Tunnel. Commun. Biol. 2021, 4, 1236 10.1038/s42003-021-02752-4. 34716402 PMC8556260

[ref78] Marino J.; von Heijne G.; Beckmann R. Small Protein Domains Fold inside the Ribosome Exit Tunnel. FEBS Lett. 2016, 590 (5), 655–660. 10.1002/1873-3468.12098. 26879042

[ref79] Su T.; Kudva R.; Becker T.; Buschauer R.; Komar T.; Berninghausen O.; von Heijne G.; Cheng J.; Beckmann R. Structural Basis of L-Tryptophan-Dependent Inhibition of Release Factor 2 by the Tnac Arrest Peptide. Nucleic Acids Res. 2021, 49 (16), 9539–9547. 10.1093/nar/gkab665. 34403461 PMC8450073

[ref80] Bhushan S.; Hoffmann T.; Seidelt B.; Frauenfeld J.; Mielke T.; Berninghausen O.; Wilson D. N.; Beckmann R. Secm-Stalled Ribosomes Adopt an Altered Geometry at the Peptidyl Transferase Center. PLoS Biol. 2011, 9, e1000581 10.1371/journal.pbio.1000581. 21267063 PMC3022528

[ref81] Berezovsky I. N.; Trifonov E. N. Evolutionary Aspects of Protein Structure and Folding. Mol. Biol. 2001, 35 (2), 233–239. 10.1023/A:1010491518368. 11357410

[ref82] Berman A. L.; Kolker E.; Trifonov E. N. Underlying Order in Protein Sequence Organization. Proc. Natl. Acad. Sci. U.S.A. 1994, 91 (9), 4044–4047. 10.1073/pnas.91.9.4044. 8171033 PMC43719

[ref83] Lewin R. Unexpected Size Pattern in Bacterial Proteins. Science 1986, 232 (4752), 825–826. 10.1126/science.3518057. 3518057

[ref84] Trifonov E. N. Segmented Genome: Elementary Units of Genome Structure. Russ. J. Genet. 2002, 38 (6), 659–663. 10.1023/A:1016091917759. 12138778

[ref85] Berne B. J.; Weeks J. D.; Zhou R. H. Dewetting and Hydrophobic Interaction in Physical and Biological Systems. Annu. Rev. Phys. Chem. 2009, 60, 85–103. 10.1146/annurev.physchem.58.032806.104445. 18928403 PMC3898792

[ref86] Giovambattista N.; Rossky P. J.; Debenedetti P. G. Effect of Temperature on the Structure and Phase Behavior of Water Confined by Hydrophobic, Hydrophilic, and Heterogeneous Surfaces. J. Phys. Chem. B 2009, 113 (42), 13723–13734. 10.1021/jp9018266. 19435300

[ref87] Kramer G.; Rauch T.; Rist W.; Vorderwulbecke S.; Patzelt H.; Schulze-Specking A.; Ban N.; Deuerling E.; Bukau B. L23 Protein Functions as a Chaperone Docking Site on the Ribosome. Nature 2002, 419 (6903), 171–174. 10.1038/nature01047. 12226666

[ref88] Raine A.; Lovmar M.; Wikberg J.; Ehrenberg M.n. Trigger Factor Binding to Ribosomes with Nascent Peptide Chains of Varying Lengths and Sequences. J. Biol. Chem. 2006, 281 (38), 28033–28038. 10.1074/jbc.M605753200. 16829677

[ref89] Akbar S.; Bhakta S.; Sengupta J. Structural Insights into the Interplay of Protein Biogenesis Factors with the 70s Ribosome. Structure 2021, 29 (7), 755 10.1016/j.str.2021.03.005. 33761323

[ref90] Schlunzen F.; Wilson D. N.; Tian P. S.; Harms J. M.; McInnes S. J.; Hansen H. A. S.; Albrecht R.; Buerger J.; Wilbanks S. M.; Fucini P. The Binding Mode of the Trigger Factor on the Ribosome: Implications for Protein Folding and Srp Interaction. Structure 2005, 13 (11), 1685–1694. 10.1016/j.str.2005.08.007. 16271892

[ref91] Baram D.; Pyetan E.; Sittner A.; Auerbach-Nevo T.; Bashan A.; Yonath A. Structure of Trigger Factor Binding Domain in Biologically Homologous Complex with Eubacterial Ribosome Reveals Its Chaperone Action. Proc. Natl. Acad. Sci. U.S.A. 2005, 102 (34), 12017–12022. 10.1073/pnas.0505581102. 16091460 PMC1183488

[ref92] Schaffitzel C.; Oswald M.; Berger I.; Ishikawa T.; Abrahams J. P.; Koerten H. K.; Koning R. I.; Ban N. Structure of the E-Coli Signal Recognition Particle Bound to a Translating Ribosome. Nature 2006, 444 (7118), 503–506. 10.1038/nature05182. 17086205

[ref93] Ullers R. S.; Houben E. N.; Raine A.; ten Hagen-Jongman C. M.; Ehrenberg M.n.; Brunner J.; Oudega B.; Harms N.; Luirink J. Interplay of Signal Recognition Particle and Trigger Factor at L23 near the Nascent Chain Exit Site on the Escherichia Coli Ribosome. J. Cell Biol. 2003, 161 (4), 679–684. 10.1083/jcb.200302130. 12756233 PMC2199365

[ref94] Noriega T. R.; Chen J.; Walter P.; Puglisi J. D. Real-Time Observation of Signal Recognition Particle Binding to Actively Translating Ribosomes. Elife 2014, 3, e04418 10.7554/eLife.04418. 25358118 PMC4213662

[ref95] Bornemann T.; Jockel J.; Rodnina M. V.; Wintermeyer W. Signal Sequence-Independent Membrane Targeting of Ribosomes Containing Short Nascent Peptides within the Exit Tunnel. Nat. Struct. Mol. Biol. 2008, 15 (5), 494–499. 10.1038/nsmb.1402. 18391966

[ref96] Jensen C. G.; Pedersen S. Concentrations of 4.5s Rna and Ffh Protein in *Escherichia Coli* - the Stability of Ffh Protein Is Dependent on Concentration of 4.5s Rna. J. Bacteriol. 1994, 176 (23), 7148–7154. 10.1128/jb.176.23.7148-7154.1994. 7525539 PMC197101

[ref97] Matsuyama S.; Fujita Y.; Sagara K.; Mizushima S. Overproduction, Purification and Characterization of Secd and Secf, Intergral Membrane Components of the Protein Translocation Machinery of *Escherichia Coli*. Biochim. Biophys. Acta 1992, 1122 (1), 77–84. 10.1016/0167-4838(92)90130-6. 1633199

[ref98] Shoji S.; Dambacher C. M.; Shajani Z.; Williamson J. R.; Schultz P. G. Systematic Chromosomal Deletion of Bacterial Ribosomal Protein Genes. J. Mol. Biol. 2011, 413 (4), 751–761. 10.1016/j.jmb.2011.09.004. 21945294 PMC3694390

[ref99] Luo H.; Lin Y.; Gao F.; Zhang C. T.; Zhang R. Deg 10, an Update of the Database of Essential Genes That Includes Both Protein-Coding Genes and Noncoding Genomic Elements. Nucleic Acids Res. 2014, 42 (D1), D574–D580. 10.1093/nar/gkt1131. 24243843 PMC3965060

[ref100] Zhang R.; Lin Y. Deg 5.0, a Database of Essential Genes in Both Prokaryotes and Eukaryotes. Nucleic Acids Res. 2009, 37, D455–D458. 10.1093/nar/gkn858. 18974178 PMC2686491

[ref101] Beechem J. M.; Gratton E. Fluorescence Spectroscopy Data Analysis Environment: A Second Generation Global Analysis Program. In Time-Resolved Laser Spectroscopy in Biochemistry; Lakowicz J. R., Ed.; SPIE, 1988; Vol. 909, pp 70–81.

[ref102] Jameson D. M.; Gratton E.; Hall R. D. The Measurement and Analysis of Heterogeneous Emissions by Multifrequency Phase and Modulation Fluorometry. Appl. Spectrosc. Rev. 1984, 20 (1), 55–106. 10.1080/05704928408081716. 6378065

[ref103] Ross J. A.; Jameson D. M. Time-Resolved Methods in Biophysics. 8. Frequency Domain Fluorometry: Applications to Intrinsic Protein Fluorescence. Photochem. Photobiol. Sci. 2008, 7 (11), 1301–1312. 10.1039/b804450n. 18958316

[ref104] Niwa T.; Kanamori T.; Ueda T.; Taguchi H. Global Analysis of Chaperone Effects Using a Reconstituted Cell-Free Translation System. Proc. Natl. Acad. Sci. U. S. A. 2012, 109 (23), 8937 10.1073/pnas.1201380109. 22615364 PMC3384135

[ref105] Niwa T.; Ying B. W.; Saito K.; Jin W.; Takada S.; Ueda T.; Taguchi H. Bimodal Protein Solubility Distribution Revealed by an Aggregation Analysis of the Entire Ensemble of *Escherichia Coli* Proteins. Proc. Natl. Acad. Sci. U.S.A. 2009, 106 (11), 4201–4206. 10.1073/pnas.0811922106. 19251648 PMC2657415

[ref106] Mecha M. F.; Hutchinson R. B.; Lee J. H.; Cavagnero S. Protein Folding *in Vitro* and in the Cell: From a Solitary Journey to a Team Effort. Biophys. Chem. 2022, 287, 106821 10.1016/j.bpc.2022.106821. 35667131 PMC9636488

[ref107] Shimizu Y.; Inoue A.; Tomari Y.; Suzuki T.; Yokogawa T.; Nishikawa K.; Ueda T. Cell-Free Translation Reconstituted with Purified Components. Nat. Biotechnol. 2001, 19 (8), 751–755. 10.1038/90802. 11479568

[ref108] Li J.; Gu L. C.; Aach J.; Church G. M. Improved Cell-Free Rna and Protein Synthesis System. PLoS One. 2014, 9 (9), e106232 10.1371/journal.pone.0106232. 25180701 PMC4152126

[ref109] Svetlov M. S.; Kommer A.; Kolb V. A.; Spirin A. S. Effective Cotranslational Folding of Firefly Luciferase without Chaperones of the Hsp70 Family. Protein Sci. 2006, 15 (2), 242–247. 10.1110/ps.051752506. 16385000 PMC2242454

[ref110] Evans M. S.; Sander I. M.; Clark P. L. Cotranslational Folding Promotes Beta-Helix Formation and Avoids Aggregation in Vivo. J. Mol. Biol. 2008, 383 (3), 683–692. 10.1016/j.jmb.2008.07.035. 18674543 PMC2597226

[ref111] Niwa T.; Uemura E.; Matsuno Y.; Taguchi H. Translation-Coupled Protein Folding Assay Using a Protease to Monitor the Folding Status. Protein Sci. 2019, 28 (7), 1252–1261. 10.1002/pro.3624. 30993770 PMC6567683

[ref112] Mounce B. C.; Kurt N.; Ellison P. A.; Cavagnero S. Nonrandom Distribution of Intramolecular Contacts in Native Single-Domain Proteins. Proteins: Struct. Funct. Bioinform. 2009, 75 (2), 404–412. 10.1002/prot.22258. 18831044

[ref113] Kurt N.; Mounce B. C.; Ellison P. A.; Cavagnero S. Residue-Specific Contact Order and Contact Breadth in Single-Domain Proteins: Implications for Folding as a Function of Chain Elongation. Biotechnol. Prog. 2008, 24 (3), 570–575. 10.1021/bp070475v. 18471028

[ref114] Zhao V.; Jacobs W. M.; Shakhnovich E. I. Effect of Protein Structure on Evolution of Cotranslational Folding. Biophys. J. 2020, 119 (6), 1123–1134. 10.1016/j.bpj.2020.06.037. 32857962 PMC7499064

[ref115] Kemp G.; Nilsson O. B.; Tian P. F.; Best R. B.; von Heijne G. Cotranslational Folding Cooperativity of Contiguous Domains of Alpha-Spectrin. Proc. Natl. Acad. Sci. U.S.A. 2020, 117 (25), 14119–14126. 10.1073/pnas.1909683117. 32513720 PMC7322005

[ref116] Walsh I. M.; Bowman M. A.; Santarriaga I. F. S.; Rodriguez A.; Clark P. L. Synonymous Codon Substitutions Perturb Cotranslational Protein Folding in Vivo and Impair Cell Fitness. Proc. Natl. Acad. Sci. U.S.A. 2020, 117 (7), 3528–3534. 10.1073/pnas.1907126117. 32015130 PMC7035613

[ref117] Fedyukina D. V.; Jennaro T. S.; Cavagnero S. Charge Segregation and Low Hydrophobicity Are Key Features of Ribosomal Proteins from Different Organisms. J. Biol. Chem. 2014, 289 (10), 6740–6750. 10.1074/jbc.M113.507707. 24398678 PMC3945335

[ref118] Houben B.; Michiels E.; Ramakers M.; Konstantoulea K.; Louros N.; Verniers J.; van der Kant R.; De Vleeschouwer M.; Chicoria N.; Vanpoucke T. Autonomous Aggregation Suppression by Acidic Residues Explains Why Chaperones Favour Basic Residues. EMBO J. 2020, 39 (11), e102864 10.15252/embj.2019102864. 32237079 PMC7265246

[ref119] Vega C. A.; Kurt N.; Chen Z.; Rüdiger S.; Cavagnero S. Binding Specificity of an Α-Helical Protein Sequence to a Full-Length Hsp70 Chaperone and Its Minimal Substrate-Binding Domain. Biochemistry 2006, 45 (46), 13835–13846. 10.1021/bi061432a. 17105202

[ref120] Kovacs N. A.; Petrov A. S.; Lanier K. A.; Williams L. D. Frozen in Time: The History of Proteins. Mol. Biol. Evol. 2017, 34 (5), 1252–1260. 10.1093/molbev/msx086. 28201543 PMC5400399

[ref121] Alvarez-Ponce D.; Aguilar-Rodriguez J.; Fares A. A. Molecular Chaperones Accelerate the Evolution of Their Protein Clients in Yeast. Genome Biol. Evol. 2019, 11 (8), 2360–2375. 10.1093/gbe/evz147. 31297528 PMC6735891

[ref122] Bogumil D.; Dagan T. Cumulative Impact of Chaperone-Mediated Folding on Genome Evolution. Biochemistry 2012, 51 (50), 9941–9953. 10.1021/bi3013643. 23167595

[ref123] Pahl A.; Brune K.; Bang H. Fit for Life? Evolution of Chaperones and Folding Catalysts Parallels the Development of Complex Organisms. Cell Stess Chapter 1997, 2 (2), 78–86. 10.1379/1466-1268(1997)002<0078:FFLEOC>2.3.CO;2. PMC312984 9250398

[ref124] Rebeaud M. E.; Mallik S.; Goloubinoff P.; Tawfik D. S. On the Evolution of Chaperones and Cochaperones and the Expansion of Proteomes across the Tree of Life. Proc. Natl. Acad. Sci. U.S.A. 2021, 118 (21), e2020885118 10.1073/pnas.2020885118. 34001607 PMC8166112

[ref125] Donis-Keller H. Site Specific Enzymatic Cleavage of Rna. Nucleic Acids Res. 1979, 7, 179–192. 10.1093/nar/7.1.179. 386279 PMC328004

[ref126] Behrmann M.; Koch H.-G.; Hengelage T.; Wieseler B.; Hoffschulte H. K.; Müller M. Requirements for the Translocation of Elongation-Arrested, Ribosome-Associated Ompa across the Plasma Membrane Ofescherichia Coli. J. Biol. Chem. 1998, 273 (22), 13898–13904. 10.1074/jbc.273.22.13898. 9593737

[ref127] Hanes J.; Plückthun A. In Vitro Selection and Evolution of Functional Proteins by Using Ribosome Display. Proc. Natl. Acad. Sci. U.S.A. 1997, 94 (10), 4937–4942. 10.1073/pnas.94.10.4937. 9144168 PMC24609

[ref128] Zhang K. Gctf: Real-Time Ctf Determination and Correction. J. Struct. Biol. 2016, 193 (1), 1–12. 10.1016/j.jsb.2015.11.003. 26592709 PMC4711343

[ref129] Watson Z. L.; Ward F. R.; Meheust R.; Ad O.; Schepartz A.; Banfield J. F.; Cate J. H. D. Structure of the Bacterial Ribosome at 2 Angstrom Resolution. Elife 2020, 9, e60482 10.7554/eLife.60482. 32924932 PMC7550191

[ref130] Pettersen E. F.; Goddard T. D.; Huang C. C.; Couch G. S.; Greenblatt D. M.; Meng E. C.; Ferrin T. E. Ucsf Chimera - a Visualization System for Exploratory Research and Analysis. J. Comput. Chem. 2004, 25 (13), 1605–1612. 10.1002/jcc.20084. 15264254

[ref131] Emsley P.; Cowtan K. Coot: Model-Building Tools for Molecular Graphics. Acta Crystallogr. Sect. D. Biol. Crystallogr. 2004, 60 (12), 2126–2132. 10.1107/S0907444904019158. 15572765

[ref132] Liebschner D.; Afonine P. V.; Baker M. L.; Bunkoczi G.; Chen V. B.; Croll T. I.; Hintze B.; Hung L. W.; Jain S.; McCoy A. J.; et al. Macromolecular Structure Determination Using X-Rays, Neutrons and Electrons: Recent Developments in Phenix. Acta Cryst. Sect. D-Struct. Biol. 2019, 75, 861–877. 10.1107/S2059798319011471. 31588918 PMC6778852

[ref133] Sievers F.; Wilm A.; Dineen D.; Gibson T. J.; Karplus K.; Li W.; Lopez R.; McWilliam H.; Remmert M.; Söding J.; et al. Fast, Scalable Generation of High-Quality Protein Multiple Sequence Alignments Using Clustal Omega. Mol. Syst. Biol. 2011, 7, 539 10.1038/msb.2011.75. 21988835 PMC3261699

[ref134] Crooks G. E.; Hon G.; Chandonia J. M.; Brenner S. E. Weblogo: A Sequence Logo Generator. Genome Res. 2004, 14 (6), 1188–1190. 10.1101/gr.849004. 15173120 PMC419797

[ref135] Schneider T. D.; Stephens R. M. Sequence Logos - a New Way to Display Consensus Sequences. Nucleic Acids Res. 1990, 18 (20), 6097–6100. 10.1093/nar/18.20.6097. 2172928 PMC332411

[ref136] Knight A. M.; Culviner P. H.; Kurt-Yilmaz N.; Zou T. S.; Ozkan S. B.; Cavagnero S. Electrostatic Effect of the Ribosomal Surface on Nascent Polypeptide Dynamics. ACS Chem. Biol. 2013, 8 (6), 1195–1204. 10.1021/cb400030n. 23517476

[ref137] Kyte J.; Doolittle R. F. A Simple Method for Displaying the Hydropathic Character of a Protein. J. Mol. Biol. 1982, 157 (1), 105–132. 10.1016/0022-2836(82)90515-0. 7108955

