# Correction to “Mapping Protein–Protein Interactions at Birth: Single-Particle Cryo-EM Analysis of a Ribosome–Nascent Globin Complex”

**DOI:** 10.1021/acscentsci.6c01105

**Published:** 2026-09-02

**Authors:** Meranda M. Masse, Rachel B. Hutchinson, Christopher E. Morgan, Neomi Millan, Joyati Das, Heather J. Allaman, Hongqing Guan, Edward W. Yu, Silvia Cavagnero

**Affiliations:** † Department of Chemistry, 5228 University of Wisconsin—Madison, Madison, Wisconsin 53706, United States; ‡ Department of Pharmacology, 2546 Case Western Reserve University, Cleveland, Ohio 44106, United States

## Overview

After the article was published, small inaccuracies in the text and minor errors in the published single-particle cryo-EM (sp-cryo-EM) structure were identified. Relevant corrections are provided here.

Some additions were also implemented, to supplement the original SDS-PAGE analysis with Western blotting. In this way, additional evidence, independent of sp-cryo-EM, provides further support for the presence of interactions between nascent apomyoglobin (apoMb) and *E. coli* ribosomal proteins L23 and L29.

In order to take care of the Additions and Corrections presented here, it was necessary to recruit two new coauthors (Neomi Millan and Joyati Das). A detailed description of the pertinent corrections and additions is provided below.

## Corrections to Main Figures and Text

### SDS-PAGE Analysis

The SDS-PAGE image of Figure 1d of the original article had some incorrectly marked molecular weight (M.W) standards. The correct MW labels are now shown in Figure 1d. In the Results and Discussion of the original article (page 388, line 4) the molecular weight of the L29 ribosomal protein was incorrectly listed as 15.8 kDa. The correct molecular weight is 7.3 kDa. Therefore, the text of page 388 (lines 4–5), should be corrected as follows: “The respective sizes of r-proteins are 11.2 kDa and 7.3 kDa. Given that these two values sum up to 18.5 kDa.”

**Figure 1:**
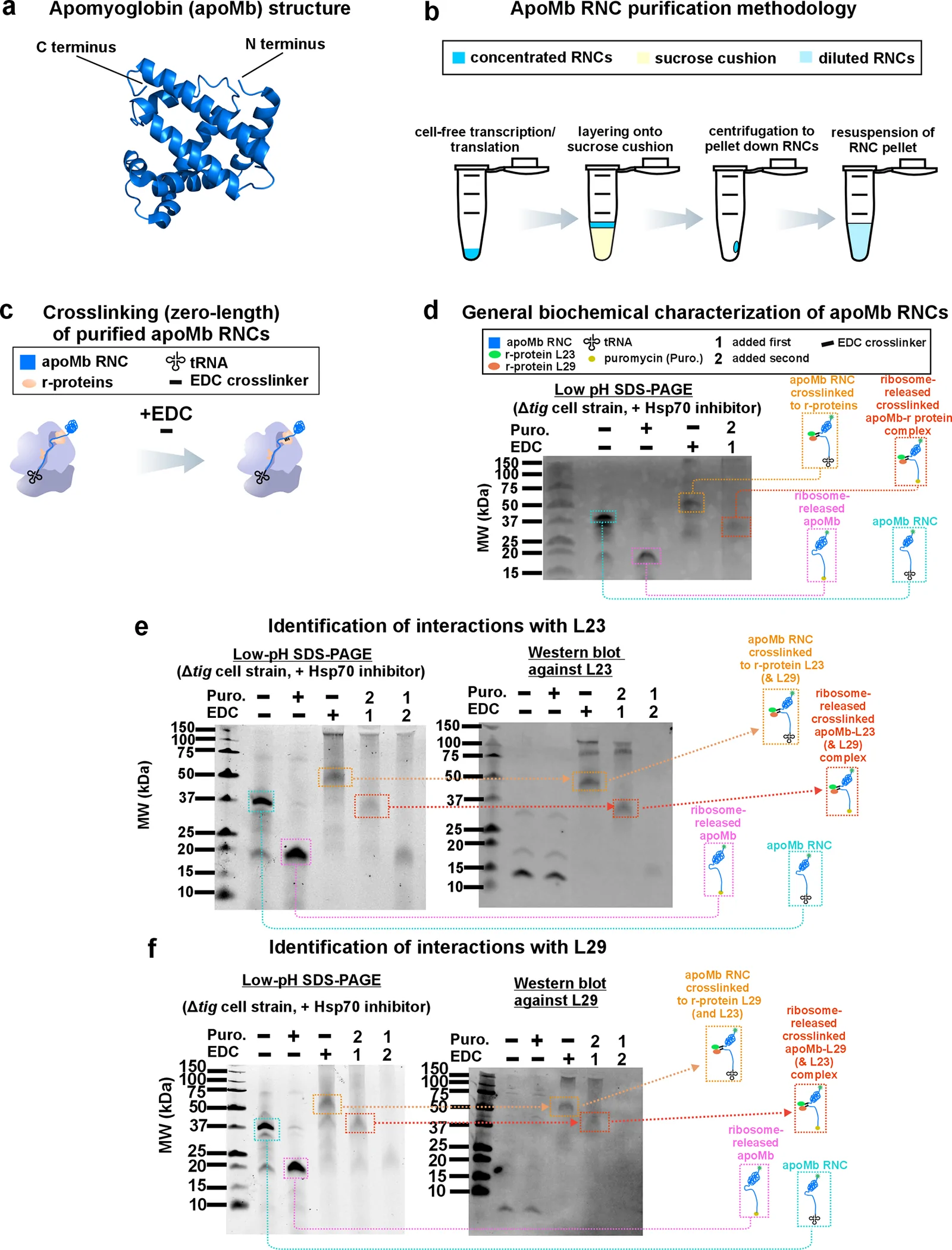
Structure of the single-domain model protein analyzed in this work and generation of ribosome-bound nascent chains (RNCs). (a) Structure of sperm whale myoglobin (apoMb), i.e., the holo-form of the model single-domain foldable protein analyzed in this work (PDB ID 1MBC). (b) Scheme illustrating the adopted purification procedure to generate apoMb RNCs for sp-cryo-EM analysis. (c) Schematic representation of the post-RNC purification treatment with the zero-length EDC cross-linking agent. (d) Representative low-pH SDS-PAGE gel illustrating RNC chemical cross-linking via EDC (*N* = 2). As a summary, in the image corresponding to this figure, the MW marker values (on the left of the gel image) were updated. (e) Low-pH SDS-PAGE and corresponding Western blots of apoMb RNCs and ribosome-released proteins, probed against ribosomal protein L23. Representative data are displayed, out of *N* = 2. (f) Low-pH SDS-PAGE and corresponding Western blots of apoMb RNCs and ribosome-released proteins, probed against ribosomal protein L29. Representative data are displayed, out of *N* = 3. Note that all the updates (including corrections and additions) to this legend are underlined, for easy reference.

### sp-cryo-EM Analysis

While reviewing the cryo-EM density map for the apomyoglobin (apoMb) ribosome-bound nascent chain (RNC) complex reported in this work, we realized that we had accidentally incorrectly assigned 2 out of 8 residues to the apoMb nascent chain (on the outer ribosomal surface) rather than to the C terminus of the L23 ribosomal protein (r-protein). This inaccuracy has now been fixed, and the images of Figure 4d and 4e have now been updated, as shown in the Figure 4 provided here.

**Figure 4:**
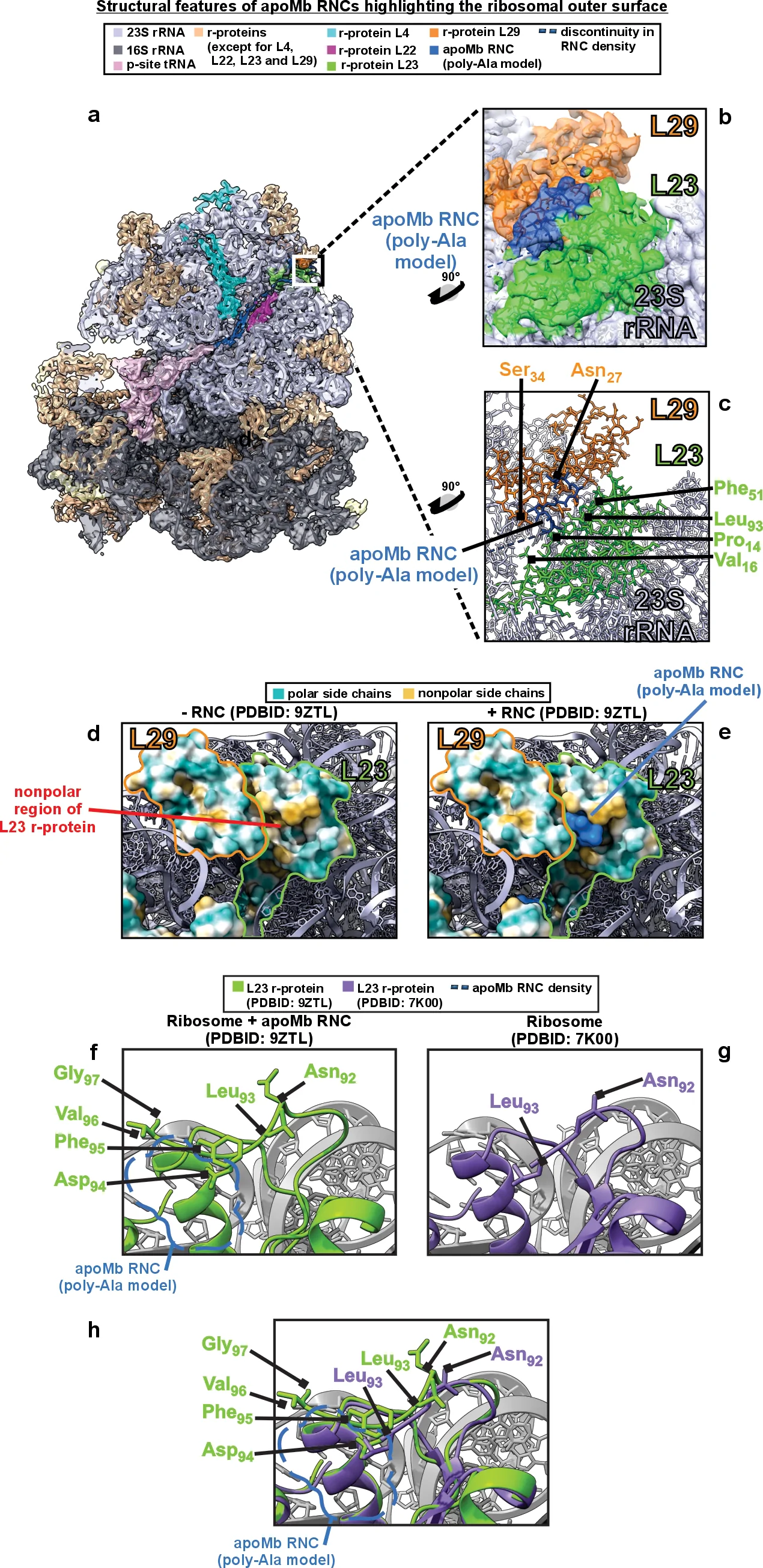
Single-particle cryo-EM structure of apoMb RNC/ribosome complex with emphasis on the ribosomal outer surface close to the tunnel exit. (a) Semitransparent cryo-EM density and corresponding structural model of apoMb RNC/ribosome complex (PDBID: 9ZTL). The ribosome-bound nascent chain (RNC) density is shown in blue. (b) Enlarged box showing nascent chain density (blue) interacting with the outer ribosomal surface close to the tunnel exit. (c) Structural model corresponding to the cryo-EM density of panel b. (d) Side view of sp-cryo-EM structural model (PDBID: 9ZTL) focusing on the L23 and L29 r-proteins facing the outer ribosomal surface, shown as molecular surface, with nonpolar and polar residues rendered in dark yellow and cyan, respectively, and with the RNC surface omitted. Color coding of polar and nonpolar residues was carried out in ChimeraX according to the scale by Kyte and Doolittle.^5^ (e) Same image as in panel d, except that the RNC surface is explicitly shown (in blue). (f) same image as in panel e, except that the PDBID 9ZTL structural model is shown, instead of the molecular surface. The C-terminal L23 residues are labeled in green, for easy identification. (g) image corresponding to the one in panels f, except that the “empty ribosome” structure (PDBID: 7K00 ) is shown. Note that the last L23 C-terminal detectable residue is Leu93. As a summary, the following additions and corrections were made to this figure: the missing panel labels (a, b, c, d and e) were added. In addition, panels d and e were replaced with corresponding images resulting from the updated analysis of the cryo-EM density (including the currently deposited 7ZTL PDBID of the nascent chain-ribosome complex). Further, new panels f, g and h were added, to document the small, yet significant, structural and dynamic differences within the L23 protein’s C terminus, for structures that either lack (7K00) or include ( 7ZTL ) the nascent protein. Note that all the updates (including corrections and additions) to this legend are underlined, for easy reference..

Rerefinement of the sp-cryo-EM structure led to minor changes in the structural validation parameters of Supporting Information Tables S1–S3. These tables have now been updated with the final correct values (see Additions and Corrections, Supporting Information Updated Tables S1–S3).

## Additions to Main Figures and Text

### SDS-PAGE and Western-Blot Analysis

To ensure a more complete cryo-EM-independent characterization of the interactions involving RNCs, Western-blotting data collection was carried out in the presence of the EDC zero-length cross-linker. The results are shown in the new panels e and f of Figure 1.

These additional data provide further support for the proximity of the apoMb nascent chain to both the L23 and L29 ribosomal proteins located at the outer surface of the ribosome, near the exit tunnel. Our results, which are cryo-EM-independent, are fully consistent with the sp-cryo-EM structural analysis of apoMb nascent chains reported in the main paper.

### sp-cryo-EM Analysis

A comparison with the published structure of the empty ribosome (see Figure 4f,g,h) shows that the C-terminal residues of the L23 r-protein beyond Leu_93_ are disordered, hence undetectable, in the empty ribosome cryo-EM structural model (PDBID: 7K00). Yet, the L23 C-terminal residues beyond Leu_93_ are identifiable in our structure (PDBID: 9ZTL), showing that the nascent chain promotes decreased dynamics of the L23 C terminus. In addition, the panel labels (a, b, c, ...) of the original Figure 4 were missing. These labels are now explicitly shown in the corrected Figure 4.

Finally, the 3D structure of the apoMb-RNC/ribosome complex has now been deposited in the RCSB Protein Data Bank (PDB) under accession code 9ZTL, and in the Electron Microscopy Data Bank (EMDB) under accession code EMD-74757.

## Additions to Materials and Methods

### Generation and Validation of anti-L23 and anti-L29 Antibodies

Rabbit polyclonal antibodies against the *E. coli* L23 and L29 ribosomal proteins were generated by Genscript (Piscataway, New Jersey). The anti-L23 antibody was raised against the CGK­VKR­HGQ­RIG­RRS epitope. This peptide corresponds to amino acids 65 to 78 of the *E. coli* L23 ribosomal protein. This type of antibody (i.e., against the same L23 epitope) was validated in previous studies.^1^ Two separate anti-L29 antibodies, were raised against each of the MKA­KEL­REK­SVE­ELC and CVA­RVK­TLL­NEK­AGA epitopes, corresponding to amino acids 1 to 14 and 50 to 62 of the *E. coli* L29 ribosomal protein, respectively. Note that these epitopes are located in L29 regions that do not interact with apoMb nascent chains, according to the sp-cryo-EM structure reported in the main paper and here. The anti-L29 antibodies were validated by Western blotting in the absence of the EDC cross-linker on a sample consisting of purified *E. coli* ribosomes carrying the apoMb nascent chain. The resulting SDS-PAGE gels and Western blots are shown in Figure 1f. As expected, a single band corresponding to free L29 (7.3 kDa) was detected. In order to rule out any potential cross reactivity of the anti-L29 antibody, two L29 peptide epitopes were compared against all *E. coli* 50S-subunit ribosomal proteins using the EMBOSS Water pairwise local sequence alignment tool.^2^ This software employs the Smith-Waterman algorithm.^3^ Supporting Information Tables S6 and S7 show that most sequences of the 50S ribosomal proteins differ substantially from the sequence of the two L29 epitopes, supporting the desired validation of the anti-L29-antibody specificity. On the other hand, a small number of ribosomal proteins displays sequence identities up to 64% and sequence similarities up to 78%. The highest similarity was observed for ribosomal protein L4. The molecular weight of this protein, however, differs substantially from that of L29 (Supporting Information Tables S6 and S7), and it is therefore unlikely to account for the band observed at the expected L29 molecular weight in Figure 1f (see bands in the absence of EDC). Given that potential antibody cross-reactivity is most relevant for proteins that comigrate during SDS-PAGE, additional attention was placed on ribosomal proteins of molecular weights similar to L29. Among them, a small number of ribosomal proteins show overall sequence identities and similarities (relative to the L29 epitopes) of 50% and 57%, respectively (Supporting-Information Tables S6 and S7). Importantly, however, these values are much lower than the 100% values pertaining to the L29 epitopes. In addition, no extended contiguous matches were identified for these proteins. Moreover, sequence alignments were also performed against all 30S-subunit ribosomal proteins. None of the 30S proteins have molecular weights comparable to that of L29. Taken together, the above sequence-alignment data and the low-molecular-weight blot bands of Figure 1f support the specificity of our anti-L29 antibodies for the *E. coli* L29 ribosomal protein.

### Identification of Interactions of apoMb RNCs with L23 and L29 Ribosomal Proteins by Western Blotting

N-terminally BODIPY-FL**-**labeled apoMb RNCs were generated by *E. coli* cell-free transcription-translation as described in the main paper, in the absence or presence of EDC cross-linker (80 mM of EDC for L23, and 25 mM for L29). Samples were then analyzed by low-pH SDS-PAGE^4^ and then transferred to polyvinylidene fluoride (PVDF) membranes. Membranes were blocked with 5% (w/v) nonfat dry milk (Bio-Rad) and incubated overnight at room temperature with rabbit polyclonal antibodies against either *E. coli* L23 (1:1,000 dilution) or *E. coli* L29 (1:500 dilution) on a gently rocking platform. Following washing with tris-buffered saline containing 0.05% (v/v) Tween 20 (TBST), the membranes were incubated for 45 min at room-temperature with AlexaFluor-Plus-647-conjugated goat antirabbit secondary antibodies (Thermo Fisher; 1:10,000 dilution). Finally, fluorescence emission intensity was detected upon excitation at 635 nm with a long-pass (LPR) filter on a fluoroimager, (GE Typhoon FLA 9500).

## Supplementary Material



## Notes

The Supporting Information has been updated to include the EMDB and PDB accession codes, corrected B-factor value and revised validation-model statistics of the sp-cryo-EM data. In addition, validation of anti-L29 antibodies via sequence-based analysis was included; Summary of cryo-EM data collection and processing parameters, cryo-EM local-resolution map of apoMb RNCs, histogram and directional FSC plot (CryoSPARC) of apoMb RNCs, Ramachandran plots, secondary-structure maps of apoMb RNCs, and uncropped gel images (PDF)

## #

Department of Chemistry and Physics, Thiel College, Greenville, PA, 16125

## ∇

Department of Chemistry, Roberts Wesleyan University, NY, 14624

## □

Thermo Fisher Scientific, Middleton, WI, 53562, USA.
