# Supplementary material for: Mapping Protein–Protein Interactions at Birth: Single-Particle Cryo-EM Analysis of a Ribosome–Nascent Globin Complex
Source: ACS Cent Sci. 2024 Feb 1;10(2):385–401. doi: 10.1021/acscentsci.3c00777 (PMC10906257; doi:10.1021/acscentsci.3c00777)
Supplement: Supplementary file 1 — oc3c00777_si_001.pdf [file oc3c00777_si_001.pdf]

Supporting Information

Mapping Protein-Protein Interactions at Birth: Single-Particle Cryo-EM Analysis of a Ribosome/Nascent-Globin Complex

Meranda M. Masse,[1] Rachel B. Hutchinson,[1,†] Christopher E. Morgan,[2,§] Heather J. Allaman,[1,ǂ] Hongqing Guan,[1] Edward W. Yu[2] & Silvia Cavagnero[1*]

[1] Department of Chemistry, University of Wisconsin-Madison, Madison, Wisconsin, 53706, USA.

[2] Department of Pharmacology, Case Western Reserve University, Cleveland, Ohio, 44106, USA.

* Corresponding author

Email address of corresponding author: cavagnero@chem.wisc.edu

Present Addresses:

[§] C. E. M.: Department of Chemistry, Thiel College, Greenville, PA, 16125

[+] R.B.H.: Department of Food Science, University of Wisconsin-Madison, Madison, Wisconsin, 53706, USA.

ǂ H.J.A.: School of Pharmacy, University of Wisconsin-Madison, Madison, Wisconsin, 53706, USA.

SUPPORTING TABLES

Supporting Table S1. Summary of single-particle cryo-electron microscopy (sp-cryo-EM) data-collection parameters and equipment.

EMDB ID	[TBD]
PDB ID	[TBD]
Microscope and detector	Titan Krios G3i and Falcon 4
Magnification and pixel size	100 and 0.947 Å/ pixel
Voltage	300 kV
Defocus range	1.5 to 2.1 µm
Total electron exposure and exposure rate	50 e-/ Å^2 and 1.5 electrons/pixel/frame
Number of frames collected per movie	5
Energy filter slit width	10 eV
Automation software	EPU v2.9 (Thermo Fisher Scientific)
Number of micrographs	9,112

Supporting Table S2. Summary of sp-cryo-EM data-processing information.

Image processing packages used for reconstruction	CryoSPARC (v3.8 – 4.2)[1] and Relion (v2.1)[2]
Number of extracted particles	3,124,095
Number of particles used for 3D reconstruction	213,770
Number of particles in final map	213,770
Masked GSFSC (cutoff at 0.143)	2.91 Å
Unmasked GSFSC (cutoff at 0.143)	3.3 Å
Local Resolution Range	2.066 – 8.045 Å
3D FSC sphericity value	N/A
Map sharpening B factor	103.9

Supporting Table S3. Sp-cryo-EM validation and model statistics.

Atomic model building package	Coot v0.9.2[3]
Atomic modeling refinement package	Phenix v1.20.1-4487 [4]
Model composition	Protein and RNA
CCVolume<br>(output from Phenix)	0.59
CCmask<br>(output from Phenix)	0.58
Mean B-factors of protein residues and nucleotides<br>(output from Phenix)	Proteins: 40.77<br>Nucleotides: 56.17
R.m.s. deviations from ideal values<br>(output from Phenix)	Length (Å): 0.008<br>Angles (°): 0.941
MolProbability score<br>(output from Phenix)	1.94
Clash score<br>(output from Phenix)	9.94
Poor rotamers<br>(output from Phenix)	3.44%
Percent of Ramachandran-favored points<br>(output from Phenix)	96.08%
Percent of Ramachandran outlier points<br>(output from Phenix)	0.50%
CaBLAM outliers<br>(output from Phenix)	3.42%
EMRinger score<br>(output from Phenix)	1.72

Supporting Table S4. Output of Clustal-Omega[5] software, documenting the conservation analysis of the L23 ribosomal protein across a variety of bacteria whose genes encoding for L23 are essential for cell viability. The yellow-highlighted columns denote the sites corresponding to the nonpolar RNC-interacting amino acids of *E. coli* L23 identified in this work (Pro14, Val16, Phe51 and Leu93). The *E. coli* amino-acid sequence is labeled with black arrows. See also Figures 5, S11 and the Materials and Methods.

```
sp|P47399|RL23_MYCGE             ---------MDVTNILLKPVLTEKSYLNQMGELKKYVFAINPKATKTKVKLAFEIIY-GV  50
sp|Q0P7S6|RL23_CAMJE             -----MADI----TDIKTILYTEKSLNLQEQ--GVVVIQTSPKMTKTGLKAVLKEYF-GV  48
sp|P66119|RL23_HELPY             -----MADI----MDIKSILYTEKSLGLQEK--GVLVVQTAQNVTKNQLKEVFKTYF-GF  48
tr|B2RLZ0|B2RLZ0_PORG3           ------------MGIIIKPIISEKMTAVTEKMSERYGFRVSPNANKIEIKKAVEAMY-NV  47
tr|E1WPD1|E1WPD1_BACF6           ------------MGIIIKPLVTEKMTAITDKL-NRFGFIVRPEANKLEIKSEVEALY-NV  46
tr|Q8A478|Q8A478_BACTN           ------------MGIIIKPLVTEKMTAITDKL-NRFGFVVRPDANKLEIKKEVEALY-NV  46
sp|P9WHB9|RL23_MYCTU             -----MATLADPRDIILAPVISEKSYGLLDD--NVYTFLVRPDSNKTQIKIAVEKIF-AV  52
sp|A3CK65|RL23_STRSV             ---------MNLYDVIKKPVITEGSMAQYEA-GKYVFEVDTRAHKLLIKQAVEAAFEGV  49
sp|P42924|RL23_BACSU             --------MKDPRDVLKRPVITERSADLMTE--KKYTFEVDVRANKTEVKDAVESIF-GV  49
sp|Q7A459|RL23_STAAN             ---------MEARDILKRPVITEKSSEAMAE--DKYTFDVDTRVNKTQVKMAVEEIF-NV  48
tr|Q98PY3|Q98PY3_MYCPU           ---------MLLSEVIKYPILTEKTYGLMEK--GIYTFAVSPKTHKIEIKKAVEFIF-NV  48
sp|Q31L09|RL23_SYNE7             ---MAEANIRALADIIRRPIITEKATRLLEN--NQYTFEVDPRASKPEIKAAIEALF-QV  54
tr|A0A9J9LE58|A0A9J9LE58_RHIWR   MAKKEAVVDNRHYDVVLRPHITEKTTLLSEH--NAVVFQVAGDASKPEIKAAVEALF-NV  57
sp|B8H4D6|RL23_CAUVN             -----MAATARHYDTILSPVITEKTTLLSEQ--NKVVFKVANDATKDEIAAAVEELF-KV  52
sp|Q8UE20|RL23_AGRFC             ------MTDLRHYDVIVSPSITEKSTLVSEQ--NQVVFNVAKTASKPEIKAAVEALF-GV  51
tr|A0A6N3JFX8|A0A6N3JFX8_FRATN   -----MSSQEKLLKTVIRPHVSDKTYGLSDAN-STIVFEVARFANKQDVKNAVEKLF-EV  53
sp|Q5NHW6|RL23_FRATT             -----MSSQEKLLKTVIRPHVSDKTYGLSDAN-STIVFEVARFANKQDVKNAVEKLF-EV  53
sp|Q6F7R4|RL23_ACIAD             ------MNNERIYQVLKGPVFSEKAQVLGETA-GVQVFKVDINATKLEIKKAVEKLF-GV  52
sp|B7IA37|RL23_ACIB5             ------MNNERIYQVLKGPVFSEKAQVLGDTA-GVQVFKVDINATKLEIKKAVEKLF-GV  52
sp|Q63Q13|RL23_BURPS             -MSEIRKNDHRLMQVLLAPVISEKATLVADKN-EQVVFEVAPDATKQEVKAAVELLF-KV  57
sp|Q2SU29|RL23_BURTA             -MSEIRKNDHRLMQVLLAPVISEKATLVADKN-EQVVFEVAPDATKQEVKAAVELLF-KV  57
sp|B4E5C2|RL23_BURCJ             -MSEIRKNDHRLMQVLLAPVISEKATLVADKN-EQVVFEVAPDATKQEVKAAVELLF-KV  57
sp|Q9HWD7|RL23_PSEAE             ------MNQERVFKVLLGPHISEKATGLADGK-SQFVFKVATDATKLEIKKAVESLF-SV  52
sp|P44361|RL23_HAEIN             ------MSQERLLSVLRAPHISEKATNNAEKS-NTVVLKVALDANKAEIAAAVAQLF-EV  52
sp|Q8EK66|RL23_SHEON             -----MIREERLLKVILAPHISEKSTVNAEKH-NTVVFRVAIDATKAEIKAAVAKLF-EV  53
sp|Q9KNY6|RL23_VIBCH             -----MIREERLLKVLRAPHISEKATMSAEKS-NTIVFKVAKDATKKEIKAAVEKLF-EV  53
tr|A0A1L3IIY2|A0A1L3IIY2_PROST   -----MIREERLLKVLRAPHVSEKASTAMEKS-NTIVLKVAKDATKAEIKAAVQKLF-EV  53
sp|P0ADZ0|RL23_ECOLI             -----MIREERLLKVLRAPHVSEKASTAMEKS-NTIVLKVAKDATKAEIKAAVQKLF-EV  53
sp|Q8XGM6|RL23_SALTI             -----MIREERLLKVLRAPHVSEKASTAMEKT-NTIVLKVAKDATKAEIKAAVQKLF-EV  53
                                                :     ::              .       *  :   .   :  .

sp|P47399|RL23_MYCGE             KPLKINTLIRKPVTIRN---GTKYPGFSKLAKLAVITLPKGM-DIAITGEKTTKKETKDQ 106
sp|Q0P7S6|RL23_CAMJE             TPKSINSLRMDGKIKR----FRGRLGQRNNYKKFYVKLPEGV-SLENTEA----------  93
sp|P66119|RL23_HELPY             EPLKINSLKQEGKVKR----FRGKLGQRKSFKKFYVKVPEGA-SIAALGA----------  93
tr|B2RLZ0|B2RLZ0_PORG3           KVEDVNTMRYDGKRKNRYTKSGLIRGKEASFKKAIVTLKKGE-TIDFFSNI---------  97
tr|E1WPD1|E1WPD1_BACF6           TVVDVNTVKYAGKNKSRYTKAGIINGRTNAFKKAIVTLKEGD-TIDFYSNI---------  96
tr|Q8A478|Q8A478_BACTN           TVVDVNTVKYAGKNKSRYTKAGIINGRTNAFKKAIVTLKEGD-TIDFYSNI---------  96
sp|P9WHB9|RL23_MYCTU             KVASVNTANRQGKRKR----TRTGYGKRKSTKRAIVTLAPGSRPIDLFGAPA-------- 100
sp|A3CK65|RL23_STRSV             KVANVNTINVKPKAKR----VGRYTGFTNKTKKAIVTLTADSKAIELFGAEEE-------  98
sp|P42924|RL23_BACSU             KVDKVNIMNYKGKSKR----VGRYTGMTSRRRKAIVKLTADSKEIEIFEA----------  95
sp|Q7A459|RL23_STAAN             KVASVNIMNYKPKKKR----MGRYQGYTNKRRKAIVTLKEGSI--DLFN-----------  91
tr|Q98PY3|Q98PY3_MYCPU           KVEKVSIINIDKKPKR----VGRFNGFTNSVKKAYVYLAQGN-SINLFPQDPSVEQELKT 103
sp|Q31L09|RL23_SYNE7             KVVGLSTQLPPRKARR----VGRFAGHRAQVKRAVARLADGD-SITLFPEV--------- 100
tr|A0A9J9LE58|A0A9J9LE58_RHIWR   KVTGVNTIVSKGKTKR----WKGTPYKRSDVKKAIVTLAEGQ-SIDVTEGVR-------- 104
sp|B8H4D6|RL23_CAUVN             KVTKVNTIVTKGKTKR----FRGIVGRRNDVKKAIVTLAEGQ-SIDITTGL---------  98
sp|Q8UE20|RL23_AGRFC             KVTAVNTLIRKGKTRR----FRGFAGKLKDVKKAVVTLAEGQ-SIDVSTGL---------  97
tr|A0A6N3JFX8|A0A6N3JFX8_FRATN   KVESVNILNVKGKARR----FGRVEGRTKAWKKAYVKLAEGH-DINFVGAE---------  99
sp|Q5NHW6|RL23_FRATT             KVESVNILNVKGKARR----FGRVEGGTKAWKKAYVKLAEGH-DINFVGAE---------  99
sp|Q6F7R4|RL23_ACIAD             DVLKVNTTITKGKSKR----FGKTLGRRSDVKKAYVTLKAGQ-DVEMADLGDTAESAAE- 106
sp|B7IA37|RL23_ACIB5             EVVKVNTTITKGKTKR----FGRTLGRRSDVKKAYVTLKAGQ-DVEMADLGDTAESAAE- 106
sp|Q63Q13|RL23_BURPS             EVDSVNVLVQKGKQKR----FGRSMGRRKDVKKAYVCLKPGQ-EINFEAEAK-------- 104
sp|Q2SU29|RL23_BURTA             EVDSVNVLVQKGKQKR----FGRSMGRRKDVKKAYVCLKPGQ-EINFEAEAK-------- 104
sp|B4E5C2|RL23_BURCJ             EVDSVNVLVQKGKQKR----FGRSMGRRKDVKKAYVCLKPGQ-EINFEAEAK-------- 104
sp|Q9HWD7|RL23_PSEAE             KVQRVTTLNVKGKTKR----TARGLGKRNDWKKAYIALQPGQ-DLDFATSAE--------  99
sp|P44361|RL23_HAEIN             KVDSVRTVVVKGKTKR----RGNKMGRRSDWKKAYVTLAEGQ-NLDFVDSAE--------  99
sp|Q8EK66|RL23_SHEON             EVESVRTLVSKGKTKR----TGGRTGRRSDWKKAYVTLAAGA-DIDFVGGAE-------- 100
sp|Q9KNY6|RL23_VIBCH             EVKSVNTLIIKGKTKR----QGLRQGRRSDVKKAYVTLNEGQ-DLDFVGGAE-------- 100
tr|A0A1L3IIY2|A0A1L3IIY2_PROST   EVEGVNTLLVKGKTKR----HGQRFGRRSDWKKAYVTLKEGQ-NLDFIGGAE-------- 100
sp|P0ADZ0|RL23_ECOLI             EVEVVNTLVVKGKVKR----HGQRIGRRSDWKKAYVTLKEGQ-NLDFVGGAE-------- 100
sp|Q8XGM6|RL23_SALTI             EVEVVNTLVVKGKVKR----HGQRIGRRSDWKKAYVTLKEGQ-NLDFVGGAE-------- 100
                                     :                          :     :  .

sp|P47399|RL23_MYCGE             ---------------------------------------------------          106
sp|Q0P7S6|RL23_CAMJE             ---------------------------------------------------           93
sp|P66119|RL23_HELPY             ---------------------------------------------------           93
tr|B2RLZ0|B2RLZ0_PORG3           ---------------------------------------------------           97
tr|E1WPD1|E1WPD1_BACF6           ---------------------------------------------------           96
tr|Q8A478|Q8A478_BACTN           ---------------------------------------------------           96
sp|P9WHB9|RL23_MYCTU             ---------------------------------------------------          100
sp|A3CK65|RL23_STRSV             ---------------------------------------------------           98
sp|P42924|RL23_BACSU             ---------------------------------------------------           95
sp|Q7A459|RL23_STAAN             ---------------------------------------------------           91
tr|Q98PY3|Q98PY3_MYCPU           KEVEAESAKTKKASDAEKRAAEKIAAKNIKSTKANTSASTPKIRVRKVADK          154
sp|Q31L09|RL23_SYNE7             ---------------------------------------------------          100
tr|A0A9J9LE58|A0A9J9LE58_RHIWR   ---------------------------------------------------          104
sp|B8H4D6|RL23_CAUVN             ---------------------------------------------------           98
sp|Q8UE20|RL23_AGRFC             ---------------------------------------------------           97
tr|A0A6N3JFX8|A0A6N3JFX8_FRATN   ---------------------------------------------------           99
sp|Q5NHW6|RL23_FRATT             ---------------------------------------------------           99
sp|Q6F7R4|RL23_ACIAD             ---------------------------------------------------          106
sp|B7IA37|RL23_ACIB5             ---------------------------------------------------          106
sp|Q63Q13|RL23_BURPS             ---------------------------------------------------          104
sp|Q2SU29|RL23_BURTA             ---------------------------------------------------          104
sp|B4E5C2|RL23_BURCJ             ---------------------------------------------------          104
sp|Q9HWD7|RL23_PSEAE             ---------------------------------------------------           99
sp|P44361|RL23_HAEIN             ---------------------------------------------------           99
sp|Q8EK66|RL23_SHEON             ---------------------------------------------------          100
sp|Q9KNY6|RL23_VIBCH             ---------------------------------------------------          100
tr|A0A1L3IIY2|A0A1L3IIY2_PROST   ---------------------------------------------------          100
sp|P0ADZ0|RL23_ECOLI             ---------------------------------------------------          100
sp|Q8XGM6|RL23_SALTI             ---------------------------------------------------          100
```

Supporting Table S5. Output of Clustal-Omega[5] software, documenting the conservation analysis of the L23 ribosomal protein across *E. coli* and a variety of bacteria whose gene encoding for L23 is non-essential for cell viability. The yellow-highlighted columns denote the sites corresponding to the nonpolar RNC-interacting amino acids of *E. coli* L23 identified in this work (Pro14, Val16, Phe51 and Leu93). The *E. coli* amino-acid sequence is labeled with black arrows. See also Figures 5, S11 and the Materials and Methods.

```
   sp|A0QL16|RL23_MYCA1             ----MATVTDPRDIILAPVISEKSYSLLD-DNVYTFVVHPDSNKTQIKIAIEKIF-SVKV  54
   sp|A0R8I2|RL23_BACAH             -------MRDPRDIIKRPVITERSMEMMA-EKKYTFDVDVKSNKTEVKDALEAIF-GVKV  51
   sp|A4VSF6|RL23_STRSY             --------MNLYDVIKKPVITESSMGQLE-AGKYVFEVDTRAHKLLIKQAVEAAFEGVKV  51
   tr|A0A2J9QGG4|A0A2J9QGG4_STRMG   --------MNLYDVIKKPVVTEKSMHALE-EGKYTFEVDTRAHKLLIKQAVEAAFDGVKV  51
   sp|C1CC08|RL23_STRZJ             --------MNLYDVIKKPVITESSMAQLE-AGKYVFEVDTRAHKLLIKQAVEAAFEGVKV  51
   tr|R4Z804|R4Z804_STRAG           --------MNLYDVIKKPVITEKSMVALE-AGKYTFEVDTRAHKLLIKQAVEAAFDGVKV  51
   sp|Q9A1X2|RL23_STRP1             --------MNLYDVIKKPVITEKSMIALE-AGKYTFEVDTRAHKLLIKQAVEAAFDGVKV  51
   sp|Q6N4T7|RL23_RHOPA             ---MKSIDPRHYDVIVAPVVTEKSTMASE-HNKVVFKVQGGATKPQIKEAVEKLF-DVKV  55
   tr|A0A258HC12|A0A258HC12_9CAUL   --MAAQPTAKHYDTILSPIITEKATILSE-QNKVVFRVAGTSTKDEIAAAVESLF-KVNV  56
   sp|Q5F5S9|RL23_NEIG1             ---MGMNQQRLTQVILVPVVSEKSNVLAEKCNQMTFKVLANATKPEIKAAVELLF-GVQV  56
   sp|Q8XV14|RL23_RALN1             MTQVAKNDHRLMQVLLSPVVSEKATLVADKNEQVVFEVARDANKGEVKAAVELLF-KVEV  59
-> sp|P0ADZ0|RL23_ECOLI             ----MIREERLLKVLRAPHVSEKASTAMEKSNTIVLKVAKDATKAEIKAAVQKLF-EVEV  55
                                                .  :  * ::* :          .: *   : *  :  *::  *  *:*

   sp|A0QL16|RL23_MYCA1             ASVNTANRQGKRKRTRTGFGKRKSTKRAIVTLAPGSKPIDLFGAPA-----          100
   sp|A0R8I2|RL23_BACAH             EKVNIMNYKPKAKRVGRHAGFTSRRRKAIVKLTADSKEIEIFQGV------           96
   sp|A4VSF6|RL23_STRSY             ANVNTINVKPKTKRVGRYVGRTNKVKKAIITLAADSKAIELFATADAE---           99
   tr|A0A2J9QGG4|A0A2J9QGG4_STRMG   ASVRTVNVKPKQKRVGRYTGFTSKTKKAIITLTADSKTIDLFAAAEAE---           99
   sp|C1CC08|RL23_STRZJ             ANVNTINVKPKAKRVGRYTGFTNKTKKAIITLTADSKAIELFAAEAE----           98
   tr|R4Z804|R4Z804_STRAG           ASVNTVTVKPKAKRVGRYTGFTSKTKKAIITLTADSKAIELFAAEAE----           98
   sp|Q9A1X2|RL23_STRP1             ASVNTVNVKPKAKRVGRYTGFTSKTKKAIITLTADSKAIELFAAEAE----           98
   sp|Q6N4T7|RL23_RHOPA             KSVNTLVRKGKTKAFRGTFGTQSDVKRAVVTLEEGH-RIDVTTGL------           99
   tr|A0A258HC12|A0A258HC12_9CAUL   LKVNTLVQKGKTKRFRGIMGRRVDIKKAIVTLADGQ-SIDVTTGL------          100
   sp|Q5F5S9|RL23_NEIG1             ASVTTVTTKGKTKRFGRILGRRSDVKKAYVSLVDGQ-ELDLEAAAAAADKE          106
   sp|Q8XV14|RL23_RALN1             ESVQILNQKGKQKRFGRFMGRRDHVKKAYVSLKPGQ-EINFEAEAK-----          104
-> sp|P0ADZ0|RL23_ECOLI             EVVNTLVVKGKVKRHGQRIGRRSDWKKAYVTLKEGQ-NLDFVGGAE-----          100
                                      *     : * *      *     ::* :.*  .   ::.
```

SUPPORTING FIGURES

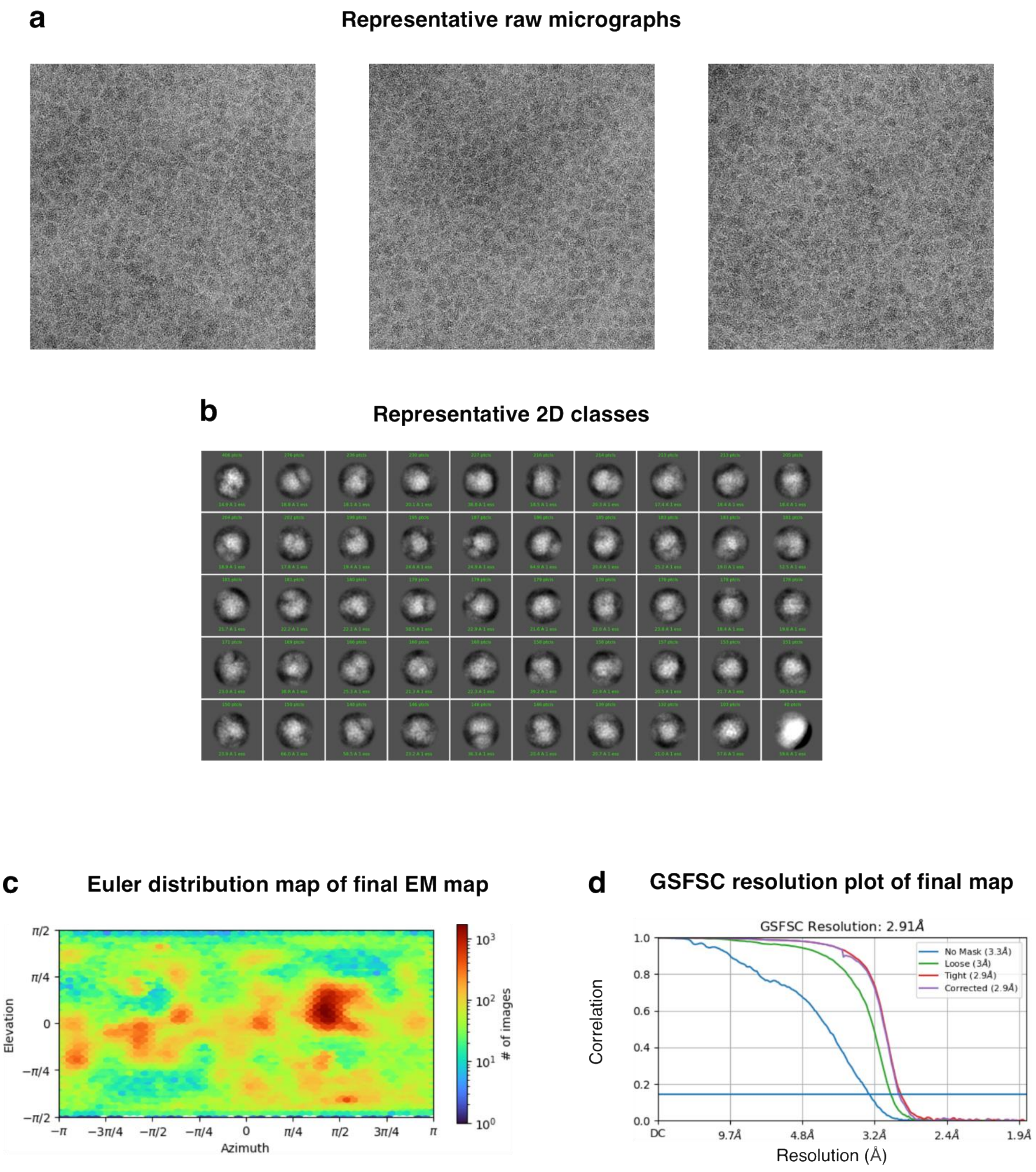

Supporting Figure S1. Sp-cryo-EM data processing information. (a) Representative raw micrographs after gain correction. (b) Representative 2D classes showing multiple projections of

the 70S ribosome. Note that, at this resolution, the nascent chain cannot be visualized. (c) Euler distribution map of the final 3D map showing that the collected particles were not in any preferred orientation, during data collection. (d) Gold Standard Fourier Shell Correlation (GSFSC) at a cutoff value of 0.143, showing that the average resolution of the solved cryo-EM map is 2.91 Å.

Data processing workflow for apoMb RNCs

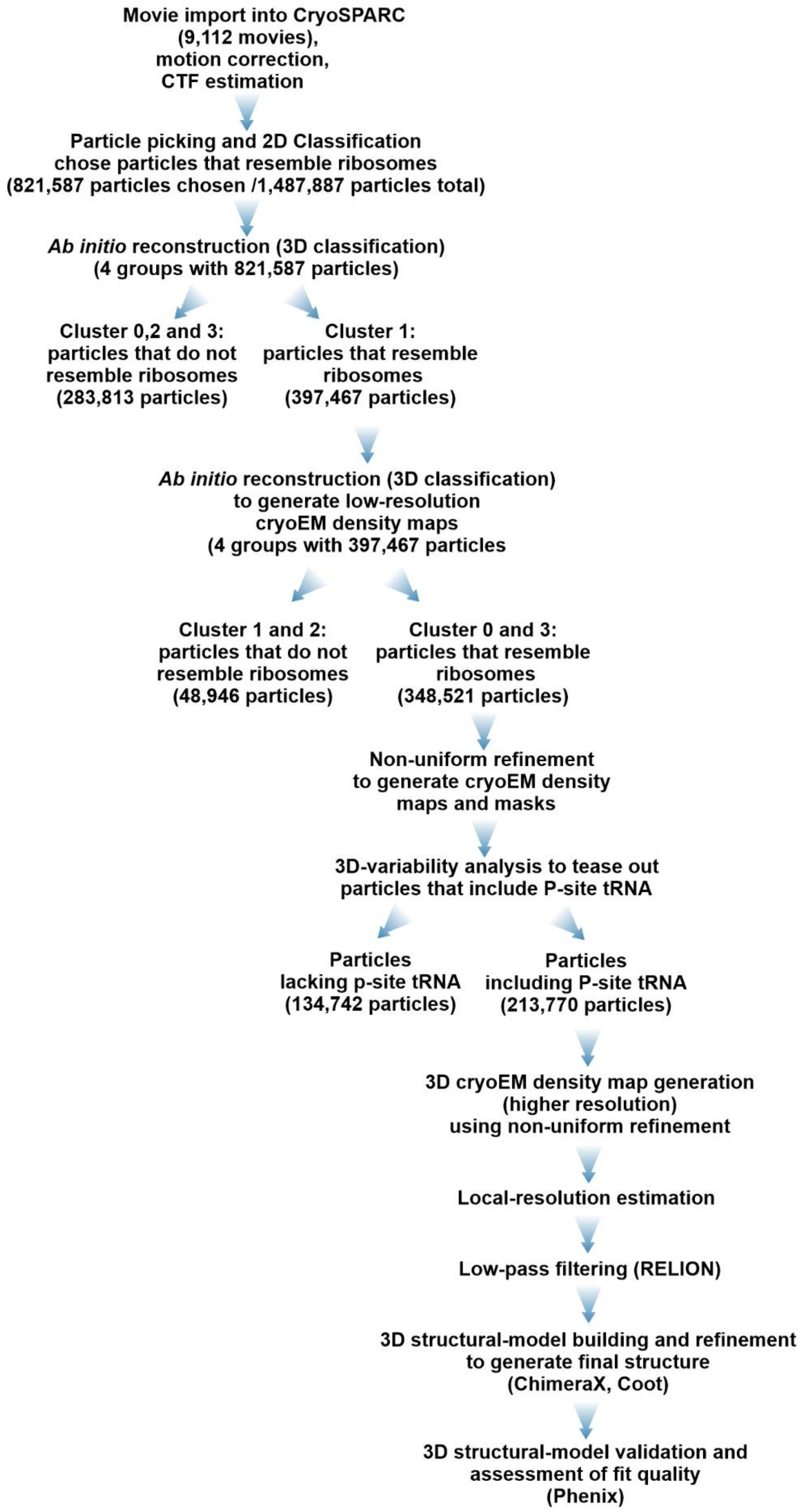

Supporting Figure S2. Cartoon illustrating the sp-cryo-EM data processing workflow. Itemized diagram illustrating the major steps involved in processing the sp-cryo-EM data presented in this study. The pertinent number of particles carried over from step to step is shown in parenthesis. The gold standard Fourier shell correlation (GSFSC) of the 3D map generated from non-uniform refinement is shown in Supplementary Figure S1.

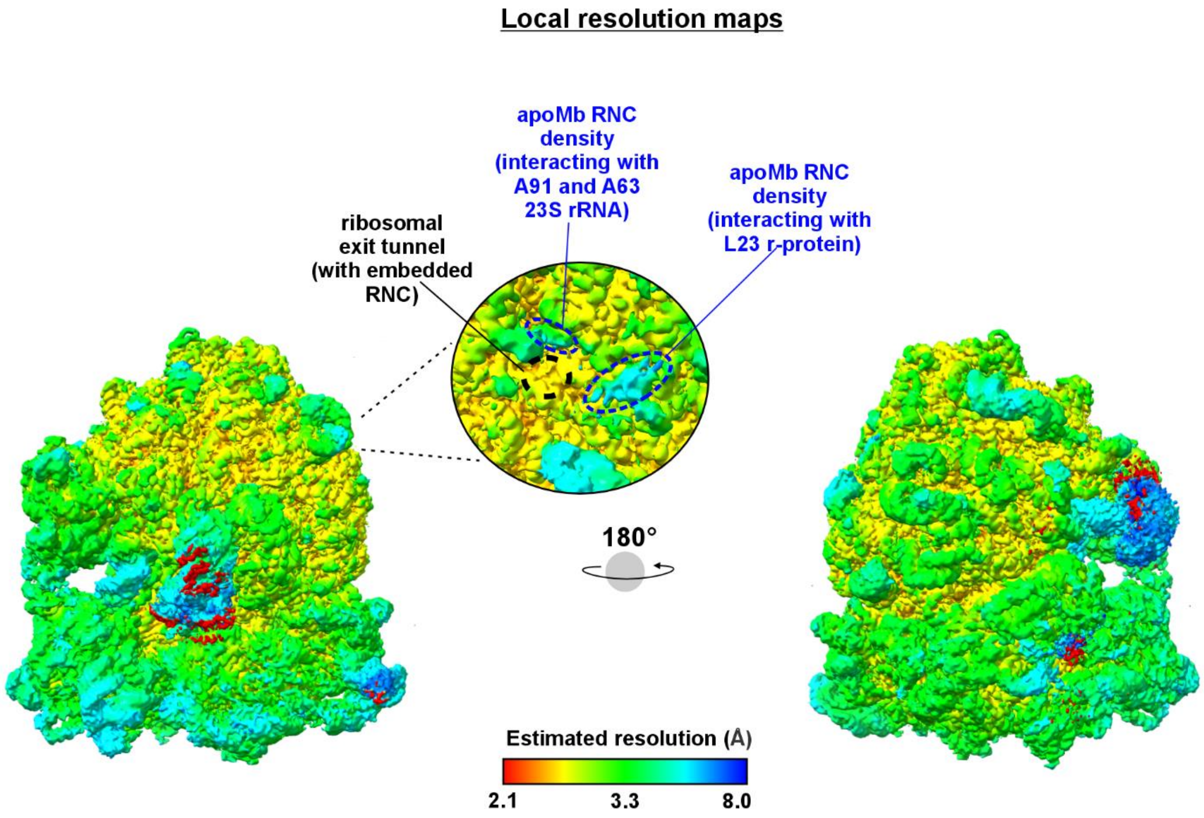

Supporting Figure S3. Sp-cryo-EM local resolution map of apoMb RNCs. The average resolution of the final map was 2.91 Å. Note that a large portion of the density corresponding to the nascent chain (RNC) has a worse resolution than the remainder of the structure. Also note that red-colored density does not correspond to a high-resolution region but is an artifact due to overfitted noise. The presence of this noise is unavoidable due to threshold requirements to adequately visualize the nascent-chain density.

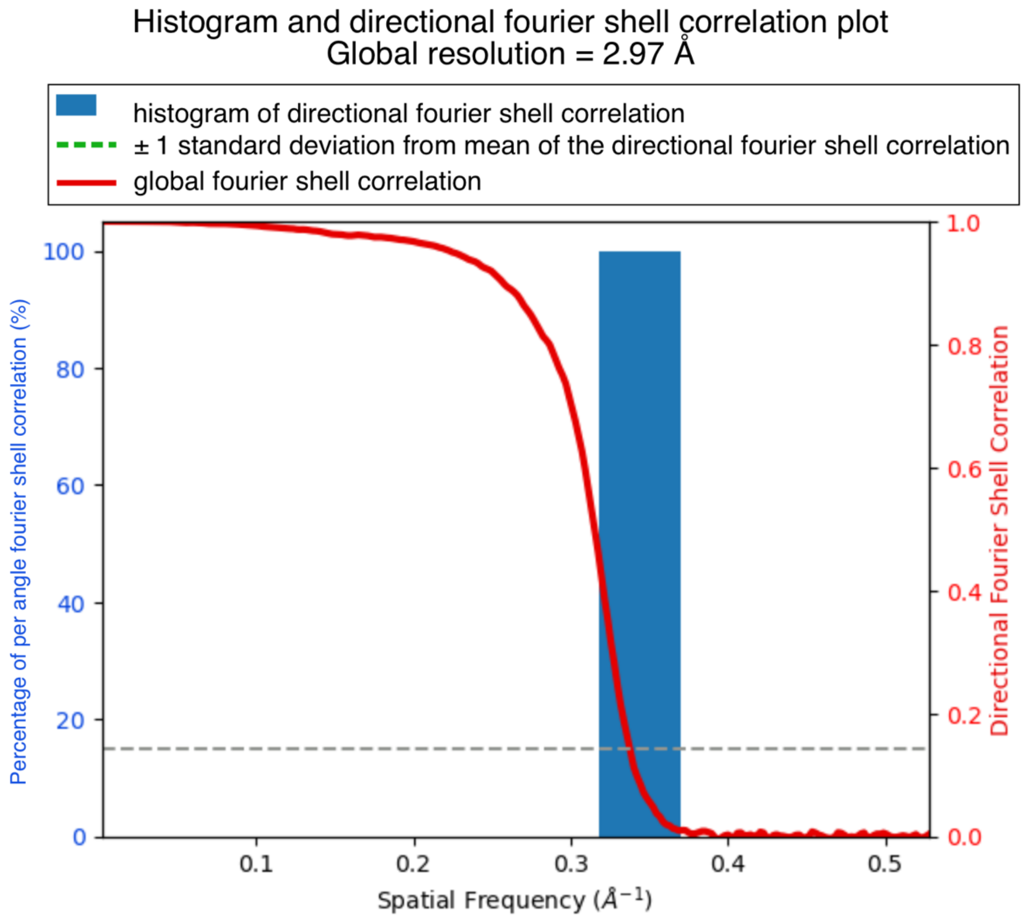

Supporting Figure S4. Histogram and directional FSC plot of final sp-cryo-EM apoMb RNCs. Plot showing the result of the 3D FSC job in CryoSPARC.

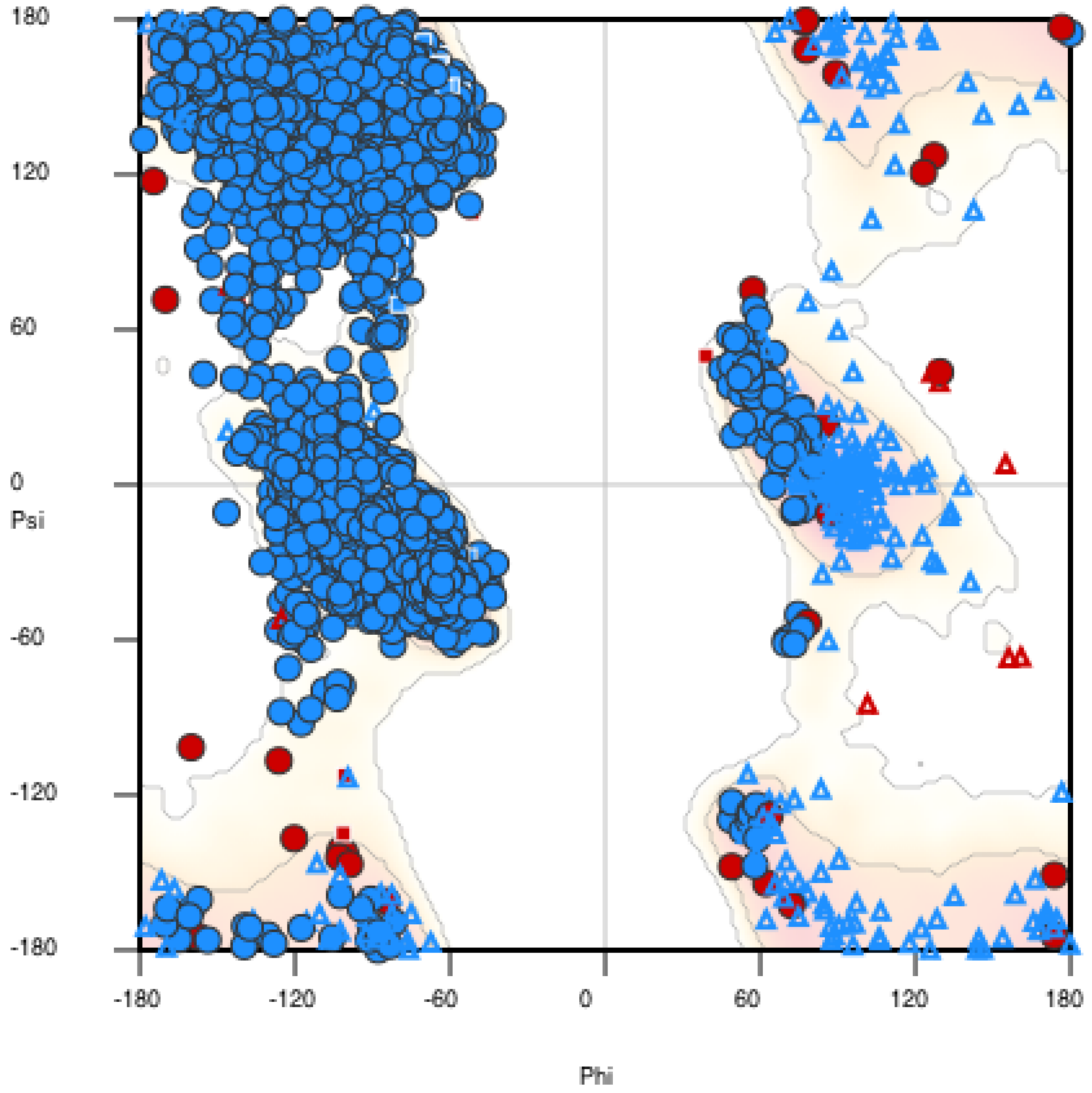

Supporting Figure S5. Ramachandran plot of the final PDB file defining the apoMb RNC/ribosome complex whose structure was determined in this work. Ramachandran plot [6] showing the Phi and Psi backbone dihedral angles of the final apoMb RNC/ribosome structural model. Blue symbols denote residues that lie within Ramachandran-compliant regions [6, 7] of the plot, while red symbols denote outlier residues. Triangles denote glycines, while squares denote prolines.

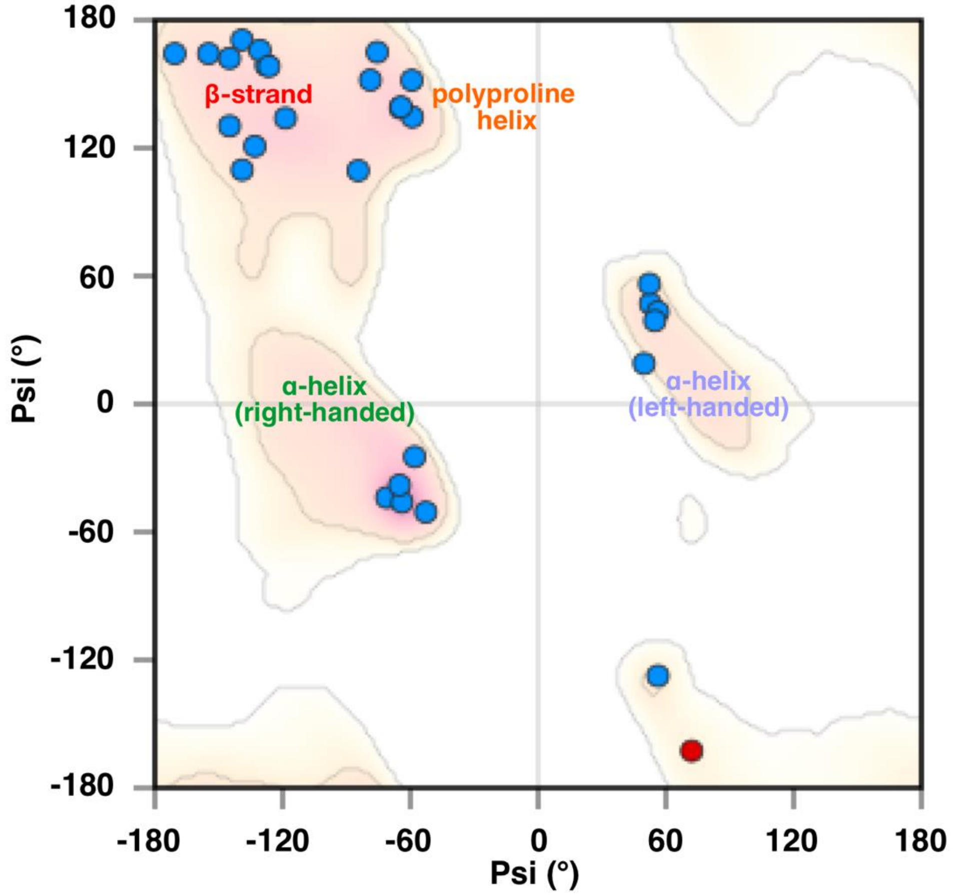

Supporting Figure S6. Ramachandran plot of the final PDB file defining the apoMb ribosome-bound nascent chain (RNC). Ramachandran plot [6] showing exclusively the Phi and Psi backbone dihedral angles of the final apoMb RNC/ribosome structural model (according to a hypothetical poly-Ala chain, as described in the main text). Blue symbols denote residues that lie within Ramachandran-compliant regions [6, 7] of the plot, while red symbols denote outlier residues.

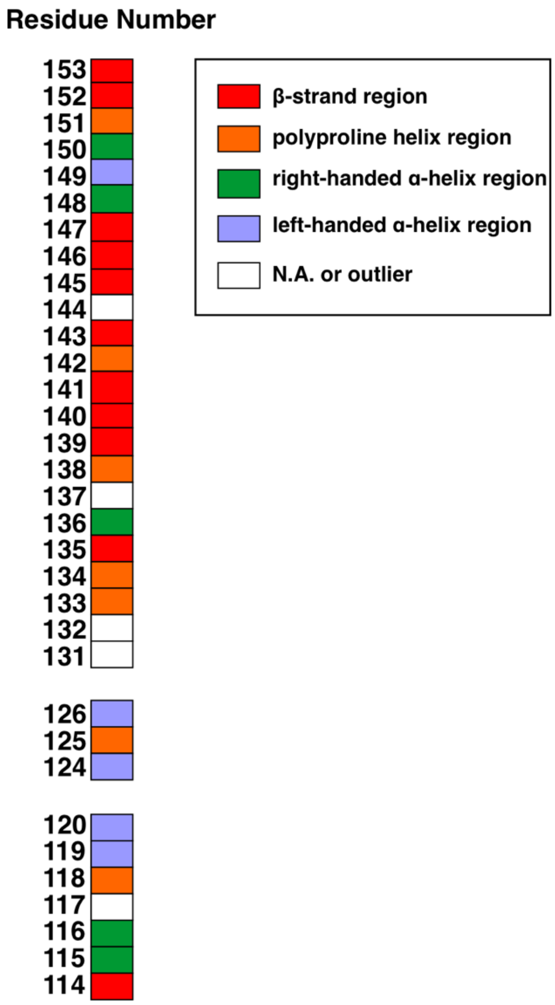

Supporting Figure S7. Map of secondary-structure regions populated by amino acids comprising the apoMb ribosome-bound nascent chain (RNC). Secondary-structure regions are defined according to Ramachandran *et al.* [6], and are deduced from the Ramachandran plot of Supplementary Figure S6. Digits on the lefthand side denote the amino acid number of the apoMb nascent-protein chain (modeled as poly-Ala). The pertinent secondary-structure regions are described in the boxed legend.

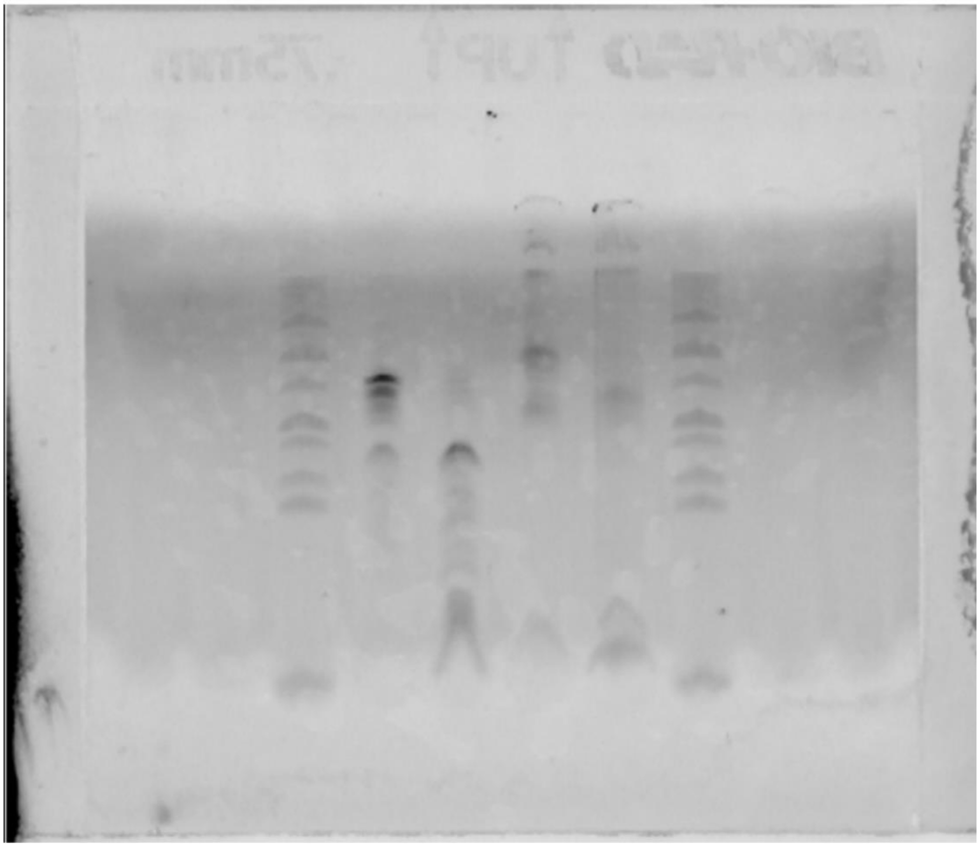

Supporting Figure S8. Uncropped gel images. Uncropped image of the low-pH SDS-PAGE gel shown in Figure 1d.

a

Fluorescence-lifetime data of apoMb ribosome-bound nascent chains (RNCs)

Non-crosslinked	EDC-crosslinked
○ Phase shift	□ Phase shift
◆ Modulation	▲ Modulation

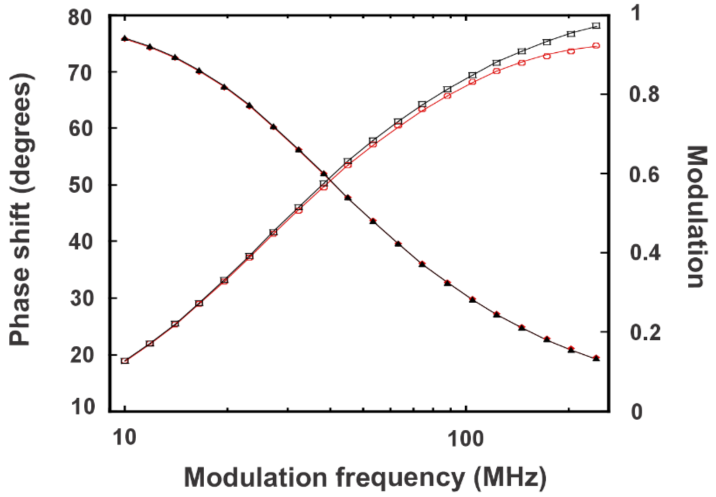

b

Fluorescence-lifetime (L1 and L2) data of apoMb ribosome-bound nascent chains (RNCs)

	Long		Short		Avg reduced χ^2	Avg life-time (ns)
	Fraction	L1 (ns)	Fraction	L2 (ns)		
Non-crosslinked	0.873 ± 0.009	5.98 ± 0.01	0.114 ± 0.007	2.0 ± 0.2	0.53 ± 0.07	5.52 ± 0.01
EDC-crosslinked	0.913 ± 0.003	5.85 ± 0.03	0.079 ± 0.003	1.76 ± 0.06	0.15 ± 0.03	5.53 ± 0.03

Supporting Figure S9. Fluorescence lifetimes of apoMb ribosome-bound nascent chains in the absence and presence of chemical crosslinking. Representative plots illustrating the experimental fluorescence-lifetime profiles of full-length apoMb RNCs in the absence (red) and presence (black) of the EDC chemical crosslinker. Data were collected by frequency-domain

fluorometry. RNC samples were first generated in an *E. coli* cell-free system in the presence of selective N-terminal labeling with the BODIPY-FL fluorophore. The RNCs were centrifuged to generate a soft ribosomal pellet, and then resuspended in buffer (see Materials and Methods), prior to spectroscopic analysis. (b) Table illustrating the fitted fluorescence lifetimes (L1 and L2) and corresponding preexponential fractions of apoMb RNCs in the absence and presence of EDC crosslinking (n=3, avg ±SE). The symbol χ^2 denotes the reduced chi-square of the fits.

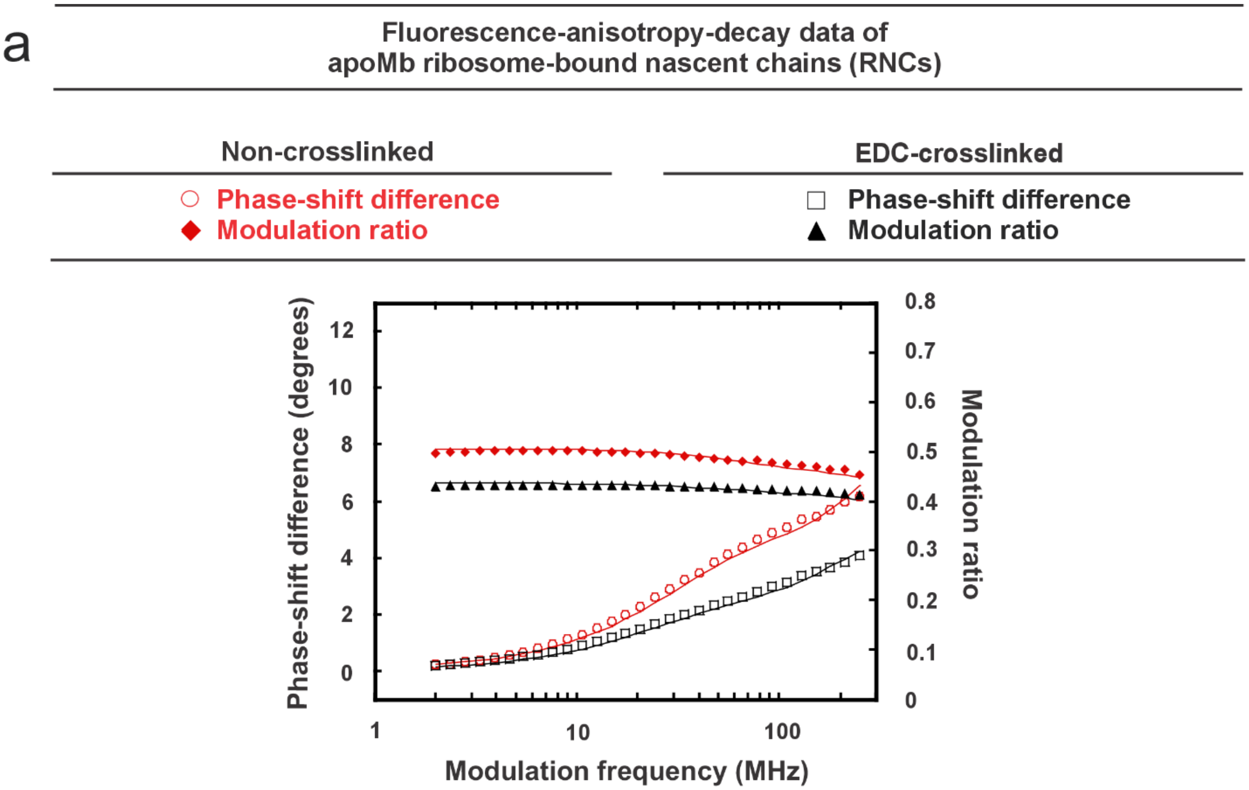

b

Fluorescence-anisotropy-decay parameters of apoMb ribosome-bound nascent chains (RNCs)

	Slow	Intermediate			Fast			Avg reduced χ^2	Predicted # of residues in compact region
	$\tau_{c,s}$ (ns)	$\tau_{c,I}$ (ns)	θ_I (°)	S_I	$\tau_{c,F}$ (ns)	θ_F (°)	S_F		
Non -cross-linked	1,000	4.0 ± 0.5	18 ± 1	0.930 ± 0.008	0.42 ± 0.03	27.6 ± 0.6	0.835 ± 0.006	2.5 ± 0.3	89 ± 10
EDC -cross-linked	1,000	9 ± 1	15.7 ± 0.2	0.945 ± 0.001	0.43 ± 0.03	19.5 ± 0.2	0.916 ± 0.002	1.02 ± 0.06	207 ± 30

Supporting Figure S10. Fluorescence anisotropy decays of apoMb ribosome-bound nascent chains in the absence and presence of chemical crosslinking. Representative plots illustrating the experimental fluorescence anisotropy decays of full-length apoMb RNCs in the absence (red) and presence (black) of the EDC 0chemical crosslinker. Data were collected by frequency-domain fluorometry.[8] RNC samples were first generated in an *E. coli* cell-free system

in the presence of selective N-terminal labeling with the BODIPY-FL fluorophore. The RNCs were centrifuged to generate a soft ribosomal pellet, and then resuspended in buffer (see Materials and Methods), prior to spectroscopic analysis. (b) Table illustrating the fitted fluorescence anisotropy-decay parameters of apoMb RNCs in the absence and presence of EDC crosslinking (n=3, avg ±SE). The symbols $t_{c,S}$, $t_{c,I}$, and $t_{c,F}$ denote rotational correlation times for the slow- (μs timescale), intermediate- (low-ns timescale), and fast- (sub-ns timescale) local motions, respectively. The symbols θ_I and θ_F denote cone semi-angles corresponding to the intermediate- and fast-timescale local motions of the RNCs, respectively. Finally, χ^2 denotes the reduced chi-square of the fits. The predicted number of residues in the compact domain, determined as described,[9] is also shown in the Table.

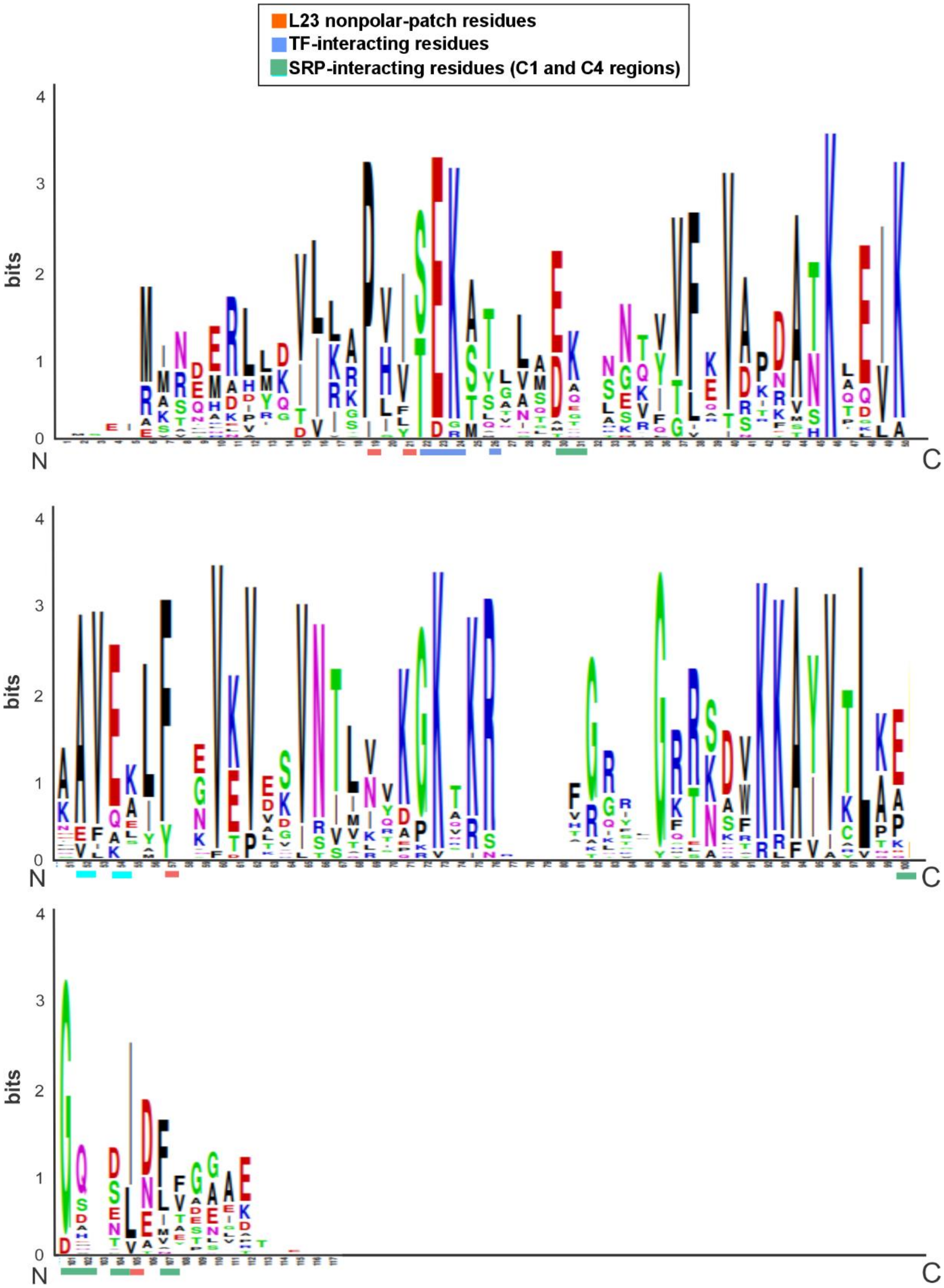

Supporting Figure S11. Conservation analysis of L23 ribosomal protein. Diagram illustrating the amino-acid conservation of the L23 ribosomal protein across bacteria whose L23 gene is essential for cell viability (based on the DEG database, see details in Materials and Methods). Conservation patterns are rendered as a logo plot.

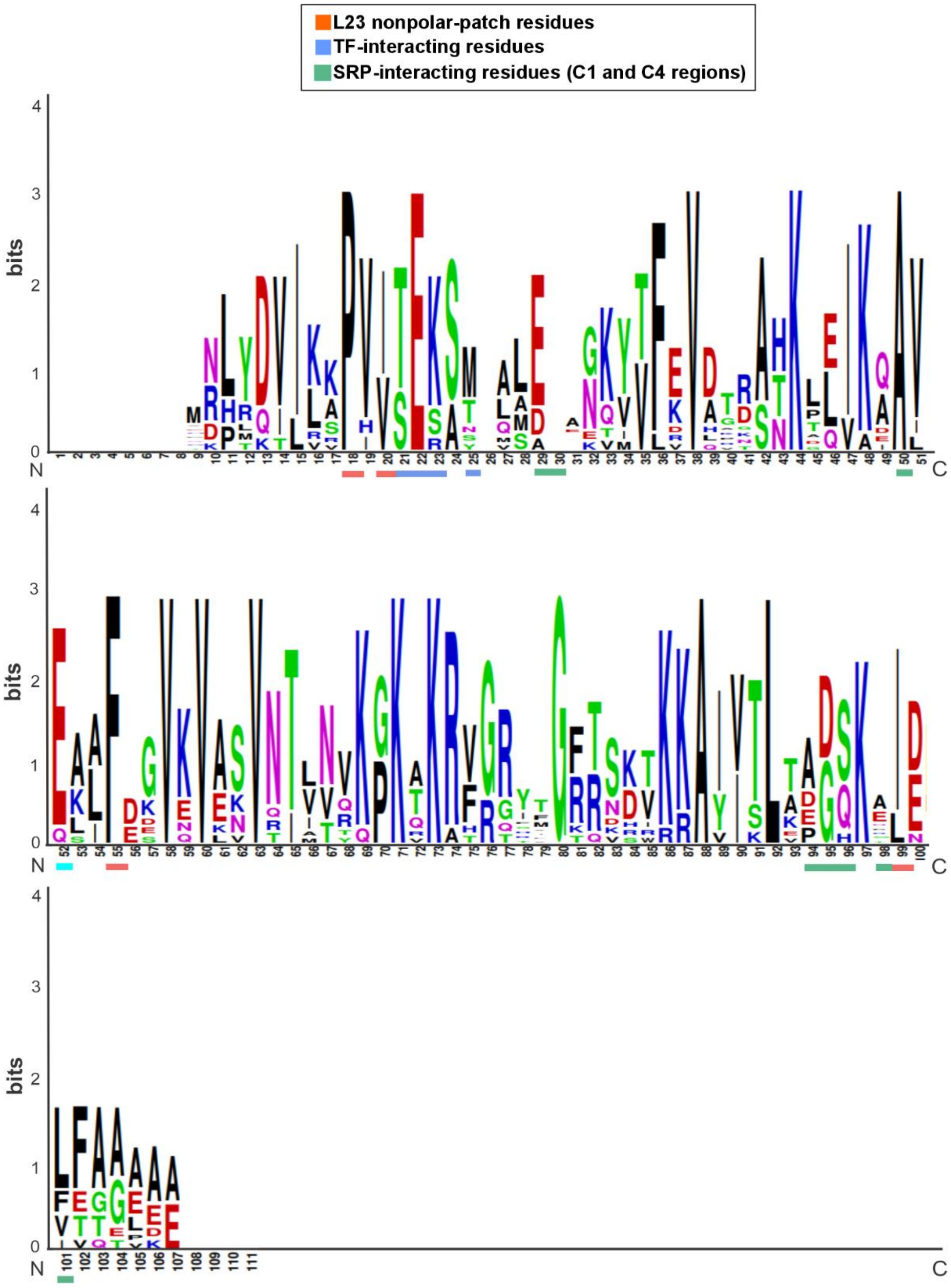

Supporting Figure S12. Additional conservation analysis of L23 ribosomal protein. Diagram illustrating the amino-acid conservation of L23 ribosomal protein across *E. coli* and other bacteria (out of the DEG database) whose L23 gene is non-essential for cell viability (see details in Materials and Methods). Conservation patterns are rendered as a logo plot.

SUPPORTING REFERENCES

(1) Punjani, A.; Rubinstein, J. L.; Fleet, D. J.; Brubaker, M. A. Cryosparc: Algorithms for Rapid Unsupervised Cryo-Em Structure Determination. *Nat. Methods* **2017**, *14* (3), 290-296.

(2) Scheres, S. H. Relion: Implementation of a Bayesian Approach to Cryo-Em Structure Determination. *J. Struct. Biol.* **2012**, *180* (3), 519-530.

(3) Emsley, P.; Cowtan, K. Coot: Model-Building Tools for Molecular Graphics. *Acta Cryst. sect. D: Biol. Cryst.* **2004**, *60* (12), 2126-2132.

(4) Adams, P. D.; Afonine, P. V.; Bunkóczi, G.; Chen, V. B.; Davis, I. W.; Echols, N.; Headd, J. J.; Hung, L.-W.; Kapral, G. J.; Grosse-Kunstleve, R. W. Phenix: A Comprehensive Python-Based System for Macromolecular Structure Solution. *Acta Cryst. Sect. D: Biol. Cryst.* **2010**, *66* (2), 213-221.

(5) Sievers, F.; Wilm, A.; Dineen, D.; Gibson, T. J.; Karplus, K.; Li, W.; Lopez, R.; McWilliam, H.; Remmert, M.; Söding, J. Fast, Scalable Generation of High-Quality Protein Multiple Sequence Alignments Using Clustal Omega. *Mol. Syst. Biol.* **2011**, *7* (1), 539.

(6) Ramachandran, G.; Ramakrishnan, C.; Sasisekharan, V. Stereochemistry of Polypeptide Chain Configurations. *J. Mol. Biol* **1963**, *7*, 95-99.

(7) Lovell, S. C.; Davis, I. W.; Adrendall, W. B.; de Bakker, P. I. W.; Word, J. M.; Prisant, M. G.; Richardson, J. S.; Richardson, D. C. Structure Validation by C Alpha Geometry: Phi,Psi and C Beta Deviation. *Proteins: Struct. Funct. Gen.* **2003**, *50* (3), 437-450.

(8) Jameson, D. M.; Gratton, E.; Hall, R. D. The Measurement and Analysis of Heterogeneous Emissions by Multifrequency Phase and Modulation Fluorometry. *Appl. Spectrosc. Rev.* **1984**, *20* (1), 55-106.

(9) Hutchinson, R. B.; Chen, X.; Zhou, N.; Cavagnero, S. Fluorescence Anisotropy Decays and Microscale-Volume Viscometry Reveal the Compaction of Ribosome-Bound Nascent Proteins. *J. Phys. Chem. B* **2021**, *125* (24), 6543-6558.
