# Supplementary material for: Mapping Protein–Protein Interactions at Birth: Single-Particle Cryo-EM Analysis of a Ribosome–Nascent Globin Complex
Source: ACS Cent Sci. 2024 Feb 1;10(2):385–401. doi: 10.1021/acscentsci.3c00777 (PMC10906257; doi:10.1021/acscentsci.3c00777)
Supplement: Supplementary file 2 — oc3c00777_si_002.pdf [file oc3c00777_si_002.pdf]

oc-2023-00777q.R1

Name: Peer Review Information for "Mapping Protein-Protein Interactions at Birth: Single-Particle Cryo-EM Analysis of a Ribosome/Nascent-Globin Complex"

First Round of Reviewer Comments

Reviewer: 1

Comments to the Author

I find it difficult when the authors write: "Finally, frequency-domain fluorescence anisotropy decay 46 experiments were carried out, to identify N-terminal dynamic regions that were undetectable by sp-cryo-EM." and these measurements have not been carried out during the preparation of the current manuscript. this is misleading.

It would be more scholarly to transparently state that the authors relate to an earlier publication - which they do. That needs be clearly stated.

Reviewer: 2

Comments to the Author

The ribosome influences the folding trajectory of nascent polypeptides as they traverse the ribosome exit tunnel and immediately adjacent to the ribosome. Studies have shown the effects of the ribosome on nascent polypeptide folding in structural and thermodynamic terms, primarily by using stalling sequences embedded in the nascent chain (NC) sequence towards the C-terminus. Although these studies have revealed numerous insights into ribosome-nascent chain interactions, much remains unknown about the molecular contacts between nascent chains and the ribosome, and their effects on protein folding. Here the authors use a truncated mRNA to trap a completed apomyoglobin (apoMb) nascent chain on the E. coli 70S ribosome, as representative of single-domain protein folding. This approach avoids the use of stalling sequences, such that a nominally more representative sequence resides in the ribosome exit tunnel near the peptidyl transferase center (PTC) and the PTC-proximal ("core") region. The authors also use a zero-length carbodiimide crosslinker EDC to trap transient NC interactions with proteins in the ribosome exit tunnel and possibly the surface of the ribosome. Using samples prepared in this way, the authors present a cryo-EM reconstruction of the RNC complex at an overall resolution of 2.9 Å, with the NC having a lower resolution as expected for a dynamic chain. The authors also use previously published fluorescence anisotropy frequency domain data to help interpret the RNC interactions they observe.

In the structure, the authors identify similar contacts to the ribosome that have been observed previously in the vicinity of the PTC and the exit tunnel core. What is new here are NC interactions with the ribosome exit tunnel vestibule region and the surface of the ribosome near the end of the exit tunnel. Based on the size of crosslinked NC species and cryo-EM density, the authors propose a model in which a nonpolar or hydrophobic patch on ribosomal protein L23 might contribute to early steps of protein folding in a manner similar to a chaperone. This region of L23 is not an overlapping binding site with that for trigger factor (TF) but is a major site of interaction with the signal recognition particle (SRP). Given the transient nature of ribosome-NC interactions, it is not yet clear whether competition between NC binding to this region of L23 and SRP would be relevant to protein folding trajectories for soluble protein domains.

Overall, the observation of surface interactions between the NC and L23 provide new structural insights, but the functional implications of the interaction and whether they are in some sense general are not clear. It is also not clear exactly what is new by adding the previously published fluorescence anisotropy data, as one of three possible models for NC folding is inconsistent with the new cryo-EM structural model independent of the fluorescence anisotropy data. The authors also don't delve into whether the nonpolar region of L23 observed to interact with the NC here is conserved across bacteria and/or across archaea and eukaryotes as well. For these reasons, the manuscript is probably better suited to another journal such as Biochemistry in its present form.

Some other issues the authors should consider addressing are given below.

1. The authors provide arguments for why the crosslinked species in Figure 1d likely involves L23 and L29. In their prior work (ref. 45, i.e.) they used western blots against L23 and L29. Why not use those approaches here?

2. In Figures 2-4, it would be helpful to show the NC density in the absence of ribosome density, i.e. mask away the ribosome density. This would help the reader see more clearly how much density is available to be filled by the NC model.

3. Supporting figure S3 is a little confusing. Presumably the red density is very low, not high as indicated. Furthermore, a slice through the exit tunnel would be more helpful to see compared to showing the left and right panel. The top panel is helpful for the surface interactions.

4. Supporting figure S5 is not really useful in the context of this paper. However supporting figures 6 and 7 could be. It is not clear, however, how well constrained the Ramachandran plot could be based on the

lower-resolution NC density. The authors should consider an alternative approach of generating a family of models consistent with the density, perhaps using Rosetta-based tools or others developed by Tom Terwilliger's group.

Author's Response to Peer Review Comments:

Silvia Cavagnero,
Professor
Department of Chemistry, Room 5357a
1101 University Avenue
Madison, WI 53706

Phone: (608) 262-5430
FAX: (608) 262-9918
E-mail:cavagnero@chem.wisc.edu

Prof. Dr. Editor
Senior Editor of *ACS Central Science*

RE: oc-2023-00777q – Manuscript Revisions

Madison, October 3 2023

Dear Prof. Editor,

I am writing to submit a revised version of the manuscript oc-2023-00777q titled "Mapping Protein-Protein Interactions at Birth: Single-Particle Cryo-EM Analysis of a Ribosome/Nascent-Globin Complex" co-authored by, Meranda Masse, Rachel B. Hutchinson, Christopher E. Morgan, Heather Allaman, Hongqing Guan, Edward Yu, and myself. This manuscript is to be considered as an Article for *ACS Central Science*. All authors have approved the submission of this revised version.

A detailed list of our responses to the reviewers' comments is provided below. For added clarity, the modifications implemented to the manuscript as a result of the comments by the reviewers are underlined. In addition, we also uploaded a marked-up copy of the revised manuscript, showing in red the changes that we implemented as a result of the reviewers' comments.

In response to Reviewer #1: This reviewer greatly appreciated the significance of our work. He/she had a constructive suggestion on how to improve the quality of the manuscript. Only minor revisions were requested. Specific responses to the comment by this reviewer are listed below.

Comment #1: I find it difficult when the authors write: "Finally, frequency-domain fluorescence anisotropy decay 46 experiments were carried out, to identify N-terminal dynamic regions that

were undetectable by sp-cryo-EM." and these measurements have not been carried out during the preparation of the current manuscript. This is misleading. It would be more scholarly to transparently state that the authors relate to an earlier publication - which they do. That needs to be clearly stated.

Response #1: We completely agree with this comment by the reviewer and we sincerely apologize about the ambiguous formulation of the original wording in the manuscript. We have now modified the pertinent sentence of the manuscript as follows: "Finally, previously acquired [46] frequency-domain fluorescence anisotropy decay experiments were critically evaluated, to help identifying N-terminal dynamic regions that were undetectable by sp-cryo-EM". We hope that the above updated wording sounds transparent, appropriate and fair.

In response to Reviewer #2: This reviewer had several constructive comments on how to improve the quality of the manuscript. Specific responses to the comments by this reviewer are listed below.

Comment #1: Overall, the observation of surface interactions between the NC and L23 provide new structural insights, but the functional implications of the interaction and whether they are in some sense general are not clear.

Response #1: Indeed, our work represents the first time the interaction between ribosomal protein L23 and a nascent protein chain outside the vestibule of the ribosomal exit tunnel has ever been reported. While the functional role of this interaction has admittedly not been elucidated yet, the fact that the interaction site on L23 is highly hydrophobic in nature is entirely unique. The highly nonpolar character of this interaction strongly suggests that its role is related to cotranslational protein folding and/or cotranslational nascent chain solubility. While future studies will be devoted to unequivocally and directly assessing the functional significance of this interaction, its mere existence and its clearly identified nonpolar character represent a significant step forward is our knowledge of the ribosomal characteristics in the context of translation.

In support of the importance of this newly identified interaction, recent publications listed below, showed that solubilization of nascent chains is one of the major functions of the ribosome.

1. Niwa, T.; Ying, B. W.; Saito, K.; Jin, W.; Takada, S.; Ueda, T.; Taguchi, H. 'Bimodal protein solubility distribution revealed by an aggregation analysis of the entire ensemble of *Escherichia coli* proteins' *Proc. Natl. Acad. Sci. USA* **106**, 4201-4206 (2009).
2. Addabbo, R.M., Hutchinson, R.B., Allaman, H.J., Dalphin, M.D., Mecha, M.F., Yue Liu, Y., Staikos, A., Cavagnero, S. 'Critical Beginnings: Selective Tuning of Solubility and Structural Accuracy of Newly Synthesized Proteins by the Hsp70 Chaperone System', *J. Phys. Chem B*, **127**, 3990-4014 (2023).
3. Addabbo, R.M., Dalphin, M.D., Mecha, M.F., Liu, Y., Staikos, A., Guzman-Luna, V., Cavagnero, S. 'Complementary Role of Co- and Post-Translational Events in *De Novo* Protein Biogenesis' *J. Phys. Chem. B* **124**, 6488-6507 (2020).

Therefore, it is highly suggestive – though not proven as of now -- that the L23-nascent-chain interaction identified here, given its nonpolar character, may contribute to preserve the solubility of nascent-chains by interacting with their nonpolar regions. While this study does not prove the functional

nature of the newly identified RNC/L23 interaction, our research moves an important step in the right direction by linking the nonpolar character of the newly discovered interaction with the known nascentchain solubilizing action of the ribosome. To take the reviewer's comment into account and to better illustrate the above concepts, we adjusted and expanded the manuscript text in the section titled "The L23 r-protein may serve as a molecular chaperone".

Comment #2: The authors also don't delve into whether the nonpolar region of L23 observed to interact with the NC here is conserved across bacteria and/or across archaea and eukaryotes as well.

Response #2: We completely agree with the reviewer on the fact that Conservation Analysis of the novel interaction site within the L23 r-protein was a notable missing aspect in our manuscript. To address this shortcoming, we carried out Conservation Analysis of the L23 ribosomal protein. The results are graphically illustrated in the following new portions of the manuscript: panel "e" of Figure 5, supporting Figure S9, supporting Table S4 and a new section of the Results titled "The nonpolar nascent-chain interaction site of L23 is highly conserved". A new Methods section describing how the analysis was performed has also been added to the manuscript. Very interestingly, the new conservation analysis shows that the L23 RNC interaction-site residues identified in this work are highly conserved across a variety of species. This finding adds significance to the results presented in this work by showing the nonpolar L23 region interacting with RNCs is recurrently present in multiple organisms. There is no doubt that addition of the conservation analysis adds value to our work, and we are extremely grateful to the reviewer for the suggestion to perform it.

Comment #3: [listed as comment 1 by the reviewer] The authors provide arguments for why the crosslinked species in Figure 1d likely involves L23 and L29. In their prior work (ref. 45, i.e.) they used western blots against L23 and L29. Why not use those approaches here?

Response #3: Indeed, as mentioned by the reviewer, a Western blot (WB) approach could have also been adopted here. However, the presence of an interaction of the apoMb RNC with the L23 ribosomal protein is already clearly established by the cryo-EM work presented here. Therefore, we believe that performing WB analysis is unnecessary in our work. In addition, the WB approach already showed RNC interactions with L23 in the case of an intrinsically disordered protein and a globin protein with similar structure to apoMb, in previously published studies. So, while certainly feasible, WB studies seem unnecessary and would not add any new information.

Comment #4: [listed as comment 2 by the reviewer] In Figures 2-4, it would be helpful to show the NC density in the absence of ribosome density, i.e. mask away the ribosome density. This would help the reader see more clearly how much density is available to be filled by the NC model.

Response #4: We thank the reviewer for this interesting comment. On the other hand, showing the nascent-chain alone would not reveal any information that is not currently available in the present display. We tried, and the density visualization ends up being very similar to that of the present display. For this reason, we elected to preserve the current display. On the other hand, we are certainly willing

to display both versions (with and without the surrounding ribosome) if the reviewer insists on this point.

Comment #5: *[listed as comment 3 by the reviewer]* *Supporting figure S3 is a little confusing. Presumably the red density is very low, not high as indicated. Furthermore, a slice through the exit tunnel would be more helpful to see compared to showing the left and right panel. The top panel is helpful for the surface interactions.*

Response #5: This is an important point and we are extremely grateful to the reviewer for bringing this item up. The red-colored density in supporting figure S3 does not correspond to a very high-resolution region but is, unfortunately, an artifact resulting from overfitted noise. The presence of this noise is unavoidable due to threshold requirements to adequately visualize the nascent-chain density. There was no way that this artifact could be avoided and visualization of ribosome slices actually produced worselooking results. Therefore, we have left the image in supporting figure s3 as is. However, to avoid incorrect interpretations and to address the reviewer's comment, we added the following text to the legend of this figure: "Note that red-colored density does not correspond to a high-resolution region but is an artifact due to overfitted noise. The presence of this noise is unavoidable due to threshold requirements to adequately visualize the nascent-chain density". We hope that this action adequately takes care of the reviewer's concern.

Comment #6: *[listed as comment 4 by the reviewer]* *Supporting figure S5 is not really useful in the context of this paper. However supporting figures 6 and 7 could be. It is not clear, however, how well constrained the Ramachandran plot could be based on the lower-resolution NC density. The authors should consider an alternative approach of generating a family of models consistent with the density, perhaps using Rosetta-based tools or others developed by Tom Terwilliger's group.*

Response #6: This is another interesting suggestion by the reviewer. We have indeed examined Rosettabased tools as suggested by the reviewer. Here are our resulting thoughts. According to Rosetta's user guide, there are three different scenarios where Rosetta could potentially be useful. The first option is using model refinement against a low-resolution structure. Unfortunately, option this requires an initial model, and while we have models of the ribosome, there are no models of the apoMb nascent chain tethered to the ribosome within the literature. The second scenario is the use of Rosetta to rebuild missing portions of a model. Unfortunately, this alternative option also requires the input of previously solved structures, which again, are not available within the literature. The third scenario, *de novo* building within a density using only a known sequence, would be the most valuable tool within Rosetta for us to use. However, this tool requires near atomic resolution and the ability to fit known sequences within the density. Unfortunately, our data are not at a sufficiently high enough resolution to use this tool. As a reference, here is the source web site that we studied in detail, to provide the above answer: https://www.rosettacommons.org/demos/latest/public/electron_density_structure_refinement/structure_re finement

Regarding the Ramachandran map in supporting figure S5, we believe that it is overall useful to retain it, and keep it as a supporting figure (but of course not in the main manuscript). Our reasoning is behind this choice is that it seems useful to the readers, at least to some extent, to appreciate that, considering

all dihedral angles of both RNC and hosting ribosome shown in this figure, there are very few dihedralangle violations in our final structure.

In summary, we made our best efforts to address the reviewers’ concerns and believe that the manuscript has substantially improved as a result of their comments. We sincerely hope that this revised version meets the requirements for publication in *ACS Central Science*. Please do not hesitate to contact me in case you have any questions.

Kindest Regards,

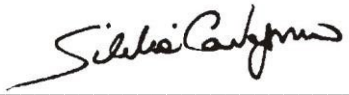

Silvia Cavagnero
Professor of Chemistry and Biochemistry,

Associate Director of Biophysics Graduate Program

oc-2023-00777q.R2

Name: Peer Review Information for "Mapping Protein-Protein Interactions at Birth: Single-Particle Cryo-EM Analysis of a Ribosome/Nascent-Globin Complex"

Second Round of Reviewer Comments

Reviewer: 2

Comments to the Author

In the revised manuscript by Masse et al., the authors now provide a conservation analysis of the nonpolar surface residues in L23 identified in the cryoEM study interacting with the apoMb N-terminal region. This improves the manuscript, but the methods do need to be clearer. How did the authors choose the L23 sequences to include in the alignment and conservation analysis? Presumably there are thousands of such sequences from bacteria that could be used, not just the 20 or so listed in the methods? It would be helpful to know how widely and evenly distributed the sequences are across bacterial phylogeny. The authors should ensure this is the case and use enough sequences for their analysis to be meaningful.

Other comments:

1. In Figure 5e, the authors could spread out the sequences a bit in the horizontal direction to make the more readable and to utilize the available space.

2. On p. 18 the authors present data that are not shown. This should be discouraged, and the authors could put it in the supplement. The caveat due to EDC crosslinking make sense, and this can be noted while still including the data in the manuscript.

3. In Figure S3, the authors can reduce the significant figures from 4 to 2 in the resolution key.

Author's Response to Peer Review Comments:

Silvia Cavagnero,
Professor
Department of Chemistry, Room 5357a
1101 University Avenue
Madison, WI 53706

Phone: (608) 262-5430
FAX: (608) 262-9918
E-mail:cavagnero@chem.wisc.edu

Prof. Dr. Editor
Senior Editor of *ACS Central Science*

RE: oc-2023-00777q.R1 – Manuscript Revision #2

Madison, December 20 2023

Dear Prof. Editor,

I am writing to submit a newly revised version of manuscript oc-2023-00777q.R1 titled "Mapping Protein-Protein Interactions at Birth: Single-Particle Cryo-EM Analysis of a Ribosome/Nascent-Globin Complex" co-authored by, Meranda Masse, Rachel B. Hutchinson, Christopher E. Morgan, Heather Allaman, Hongqing Guan, Edward Yu, and myself. This manuscript is to be considered as an Article for *ACS Central Science*. All authors have approved the submission of this 2nd revision.

A detailed list of our responses to the reviewers' comments is provided below. For added clarity, the modifications implemented to the manuscript as a result of the comments by the reviewers are underlined. In addition, we also uploaded a marked-up copy of the revised manuscript, showing in red the changes that we implemented as a result of the reviewers' comments.

In response to Reviewer #2: This reviewer had a few additional helpful comments on how to further improve the quality of the manuscript. Specific responses to the comments by this reviewer are listed below.

Comment #1: In the revised manuscript by Masse et al., the authors now provide a conservation analysis of the nonpolar surface residues in L23 identified in the cryoEM study interacting with the apoMb N-terminal region. This improves the manuscript, but the methods do need to be clearer. How did the authors choose the L23 sequences to include in the alignment and conservation analysis? Presumably there are thousands of such sequences from bacteria that could be used, not just the 20 or so listed in the methods? It would be helpful to know how widely and evenly distributed the sequences are across bacterial phylogeny. The authors should ensure this is the case and use enough sequences for their analysis to be meaningful.

Response #1: We agree with this comment, and we have now provided a more comprehensive conservation analysis, including more organisms. Importantly, we have included more detailed written descriptions to justify the choice of the bacteria employed in the conservation analysis. The text accompanying the improved conservation analysis is provided both in the Results and Discussion (pages 16-17) and in the Materials and Methods (pages 25-26). In addition, we have generated improved and new Tables (supporting Tables 4 and 5) and Figures (supporting Figures S11 and S12) to better describe the choices made and provide additional details. Briefly, the gene encoding the L23 ribosomal protein is essential for cell viability in *E. coli*. Thus, we carried out the conservation analysis across all bacteria of a database known as DEG (Database of Essential Genes), which contains genomes of organisms that bear one or more essential genes. First, we focused on the bacteria in the DEG database where L23 serves as an essential protein. Next, we performed an additional new conservation analysis comparing *E. coli* L23 to all L23s in bacteria (within the DEG database) where L23 is non-essential. It is worth notig that, while the conservation analysis reported in this work is limited to the bacteria in DEG database, more comprehensive comparisons across a wider set of bacteria could and should also be carried out in the future. However, these comparisons are beyond the scope of the present study, which focuses on the RNC structure and dynamics in *E. coli* and on RNC interactions with the ribosomal surface.

Comment #2: In Figure 5e, the authors could spread out the sequences a bit in the horizontal direction to make the more readable and to utilize the available space.

Response #2: We are thankful to the reviewer for this comment, and we have updated the conservationanalysis diagrams in Figure 5e so that they are now horizontally spread as much as possible, to facilitate inspection and readability.

Comment #3: On p. 18 the authors present data that are not shown. This should be discouraged, and the authors could put it in the supplement. The caveat due to EDC crosslinking make sense, and this can be noted while still including the data in the manuscript.

Response #3: We are glad about this request by the reviewer, and we have now added supporting Figures S9 and S10, which include the lifetime and anisotropy results originally mentioned in the manuscript but not explicitly shown before. We have also cited these new figures and properly adjusted the text in the Results and Discussion (page 19). Finally, we have added a new section in the Materials and Methods, to properly describe how the fluorescence lifetime and anisotropy-decay experiments were carried out.

Comment #4: In Figure S3, the authors can reduce the significant figures from 4 to 2 in the resolution key.

Response #4: In response to this comment by the reviewer, we were happy to reduce the number of significant figures in the index bar of Figure S3 (Supporting Information) , as requested by the reviewer.

In response to the Formatting Updates requested by the ACS Central Science Editorial Office:

Comment #1: SI PG#S: The supporting information pages must be numbered consecutively, starting with page S1.

Response #1: The pages of the Supporting Information have now been appropriately renumbered, as requested.

Comment #2: SI AU EMAIL: Please include the email address of the corresponding author on the first the Supporting Information, with an asterisk next to their name in the author list.

Response #2: We have taken care of this requested update.

Comment #3: AU EMAIL: Please include the email address of the corresponding author on the first page of the manuscript, and the Supporting Information if submitted, with an asterisk next to their name in the author list. Please be sure to label "email."

Response #3: We have taken care of this requested update.

In addition to addressing the above comments by the reviewers and the requested formatting changes, we have also corrected some typographical errors and made small adjustments to Figures 6 and 7 and the accompanying text, to further improve clarity and better summarize our results.

In summary, we made our best efforts to address the comments by reviewer #2, and believe that the manuscript has substantially improved as a result of our manuscript updates. We sincerely hope that this newly revised version meets the requirements for publication in *ACS Central Science*. Please do not hesitate to contact me if you have any questions.

Kindest Regards,

Silvia Cavagnero
Professor of Chemistry and Biochemistry,
Associate Director of Biophysics Graduate Program
