# Supplementary material for: Correction to “Mapping Protein–Protein Interactions at Birth: Single-Particle Cryo-EM Analysis of a Ribosome–Nascent Globin Complex”
Source: ACS Cent Sci. 2026 Sep 2;12(9):1550–4. doi: 10.1021/acscentsci.6c01105 (PMC13614039; doi:10.1021/acscentsci.6c01105)
Supplement: Supplementary file 1 [file oc6c01105_si_001.pdf]

Correction to

Supporting Information

Mapping Protein-Protein Interactions at Birth: Single-Particle Cryo-EM Analysis of a Ribosome/Nascent-Globin Complex

Meranda M. Masse,[1] Rachel B. Hutchinson,[1,†] Christopher E. Morgan,[2,§] Neomi Millan,[1] Joyati Das,[1] Heather J. Allaman,[1,ǂ] Hongqing Guan,[1] Edward W. Yu[2] & Silvia Cavagnero[1]

ACS Cent. Sci. 2024 10, 385-401. DOI: 10.1021/acscentsci.3c00777

Coauthors Neomi Millan and Joyati Das were added.

[1] Department of Chemistry, University of Wisconsin-Madison, Madison, Wisconsin, 53706, USA.

[2] Department of Pharmacology, Case Western Reserve University, Cleveland, Ohio, 44106, USA.

* Corresponding author

Email address of corresponding author: cavagnero@chem.wisc.edu

Present Addresses:

[§] C. E. M.: Department of Chemistry and Physics, Thiel College, Greenville, PA, 16125

[†] R.B.H.: Department of Chemistry, Roberts Wesleyan University, NY, 14624

[ǂ] H.J.A.: Thermo Fisher Scientific, Middleton, WI, 53562, USA.

NOTE OF INTEREST

This is an updated version of the Supplementary Information (complete document) associated with the original article. The Supporting Information has been updated to include the EMDB and PDB accession codes of the apomyoglobin (apoMb) ribosome-bound nascent chain (RNC, Table S1), the corrected B-factor value for the density map (Table S2) and the revised validation-model statistics of the sp-cryo-EM data (Table S3). In addition, we created new tables (Tables S6 and S7) documenting the validation of the anti-L29 antibodies based on sequence-similarity and identity analysis.

In order to clearly indicate the implemented changes, the newly added text is underlined throughout the document.

SUPPORTING TABLES

Updated Supporting Table S1. Summary of single-particle cryo-electron microscopy (sp-cryo-EM) data-collection parameters and equipment. As a summary, in this table the EMDB and PDB accession codes were updated.

EMDB ID	EMD-74757
PDB ID	9ZTL
Microscope and detector	Titan Krios G3i and Falcon 4
Magnification and pixel size	100 and 0.947 Å/pixel
Voltage	300 kV
Defocus range	1.5 to 2.1 µm
Total electron exposure and exposure rate	50 e-/Å^2 and 1.5 electrons/pixel/frame
Number of frames collected per movie	5
Energy filter slit width	10 eV
Automation software	EPU v2.9 (Thermo Fisher Scientific)
Number of micrographs	9,112

Updated Supporting Table S2. Sp-cryo-EM data-processing information. As a summary, in this table, the map sharpening B factor value was updated.

Image processing packages used for reconstruction	CryoSPARC (v3.8 – 4.2)[1] Relion (v2.1)[2]
Number of extracted particles	3,124,095
Number of particles used for 3D reconstruction	213,770
Number of particles in final map	213,770
Masked GSFSC (cutoff at 0.143)	2.91 Å[a]
Unmasked GSFSC (cutoff at 0.143)	3.3 Å
Local Resolution Range	2.066 – 8.045 Å
3D FSC sphericity value	N/A
Map sharpening B factor	55.6

[a] Final map used for reconstruction low-pass filtered to 4.0 Å for model building and refinement.

Updated Supporting Table S3. Sp-cryo-EM validation and model statistics. As a summary, in this table, the CCVolume, CCmask, mean B-factors of pr0otein residues and nucleotides, R.m.s. deviations from ideal values, MolProbity score, clash score, poor rotamers, percent of Ramachandran-favored points, percent of Ramachandran-outlier points, and CaBLAM values were updated.

Atomic model building package	Coot v0.9.2[3]
Atomic modeling refinement package	Phenix v1.20.1-4487[4]
Model composition	Protein and RNA
CCVolume<br>(output from Phenix)	0.90
CCmask<br>(output from Phenix)	0.92
Mean B-factors of protein residues and nucleotides<br>(output from Phenix)	Proteins: 59.35<br>Nucleotides: 60.43
R.m.s deviations from ideal values<br>(output from Phenix)	Length (Å): 0.009<br>Angles (˚): 0.928
MolProbity score<br>(output from Phenix)	2.17
Clash score<br>(output from Phenix)	9.12
Poor rotamers<br>(output from Phenix)	2.50%
Percent of Ramachandran-favored points<br>(output from Phenix)	94.37%
Percent of Ramachandran outlier points<br>(output from Phenix)	0.16%
CaBLAM outliers<br>(output from Phenix)	2.56%

Supporting Table S4. Output of Clustal-Omega[5] software, documenting the conservation analysis of the L23 ribosomal protein across a variety of bacteria whose genes encoding for L23 are essential for cell viability. The yellow-highlighted columns denote the sites corresponding to the nonpolar RNC-interacting amino acids of *E. coli* L23 identified in this work (Pro14, Val16, Phe51 and Leu93). The *E. coli* amino-acid sequence is labeled with black arrows. See also Figures 5, S11 and the Materials and Methods.

```
  sp|P47399|RL23_MYCGE           ---------MDVTNILLKPVLTEKSYLNQMGELKKYVFAINPKATKTKVKLAFEIIY-GV  50
  sp|Q0P7S6|RL23_CAMJE           -----MADI----TDIKTILYTEKSLNLQEQ--GVVVIQTSPKMTKTGLKAVLKEYF-GV  48
  sp|P66119|RL23_HELPY           -----MADI----MDIKSILYTEKSLGLQEK--GVLVVQTAQNVTKNQLKEVFKTYF-GF  48
  tr|B2RLZ0|B2RLZ0_PORG3         ------------MGIIIKPIISEKMTAVTEKMSERYGFRVSPNANKIEIKKAVEAMY-NV  47
  tr|E1WPD1|E1WPD1_BACF6         ------------MGIIIKPLVTEKMTAITDKL-NRFGFIVRPEANKLEIKSEVEALY-NV  46
  tr|Q8A478|Q8A478_BACTN         ------------MGIIIKPLVTEKMTAITDKL-NRFGFVVRPDANKLEIKKEVEALY-NV  46
  sp|P9WHB9|RL23_MYCTU           -----MATLADPRDIILAPVISEKSYGLLDD--NVYTFLVRPDSNKTQIKIAVEKIF-AV  52
  sp|A3CK65|RL23_STRSV           ---------MNLYDVIKKPVITEGSMAQYEA—GKYVFEVDTRAHKLLIKQAVEAAFEGV   49
  sp|P42924|RL23_BACSU           --------MKDPRDVLKRPVITERSADLMTE--KKYTFEVDVRANKTEVKDAVESIF-GV  49
  sp|Q7A459|RL23_STAAN           ---------MEARDILKRPVITEKSSEAMAE--DKYTFDVDTRVNKTQVKMAVEEIF-NV  48
  tr|Q98PY3|Q98PY3_MYCPU         ---------MLLSEVIKYPILTEKTYGLMEK--GIYTFAVSPKTHKIEIKKAVEFIF-NV  48
  sp|Q31L09|RL23_SYNE7           ---MAEANIRALADIIRRPIITEKATRLLEN--NQYTFEVDPRASKPEIKAAIEALF-QV  54
  tr|A0A9J9LE58|A0A9J9LE58_RHIWR MAKKEAVVDNRHYDVVLRPHITEKTTLLSEH--NAVVFQVAGDASKPEIKAAVEALF-NV  57
  sp|B8H4D6|RL23_CAUVN           -----MAATARHYDTILSPVITEKTTLLSEQ--NKVVFKVANDATKDEIAAAVEELF-KV  52
  sp|Q8UE20|RL23_AGRFC           ------MTDLRHYDVIVSPSITEKSTLVSEQ--NQVVFNVAKTASKPEIKAAVEALF-GV  51
  tr|A0A6N3JFX8|A0A6N3JFX8_FRATN -----MSSQEKLLKTVIRPHVSDKTYGLSDAN-STIVFEVARFANKQDVKNAVEKLF-EV  53
  sp|Q5NHW6|RL23_FRATT           -----MSSQEKLLKTVIRPHVSDKTYGLSDAN-STIVFEVARFANKQDVKNAVEKLF-EV  53
  sp|Q6F7R4|RL23_ACIAD           ------MNNERIYQVLKGPVFSEKAQVLGETA-GVQVFKVDINATKLEIKKAVEKLF-GV  52
  sp|B7IA37|RL23_ACIB5           ------MNNERIYQVLKGPVFSEKAQVLGDTA-GVQVFKVDINATKLEIKKAVEKLF-GV  52
  sp|Q63Q13|RL23_BURPS           -MSEIRKNDHRLMQVLLAPVISEKATLVADKN-EQVVFEVAPDATKQEVKAAVELLF-KV  57
  sp|Q2SU29|RL23_BURTA           -MSEIRKNDHRLMQVLLAPVISEKATLVADKN-EQVVFEVAPDATKQEVKAAVELLF-KV  57
  sp|B4E5C2|RL23_BURCJ           -MSEIRKNDHRLMQVLLAPVISEKATLVADKN-EQVVFEVAPDATKQEVKAAVELLF-KV  57
  sp|Q9HWD7|RL23_PSEAE           ------MNQERVFKVLLGPHISEKATGLADGK-SQFVFKVATDATKLEIKKAVESLF-SV  52
  sp|P44361|RL23_HAEIN           ------MSQERLLSVLRAPHISEKATNNAEKS-NTVVLKVALDANKAEIAAAVAQLF-EV  52
  sp|Q8EK66|RL23_SHEON           -----MIREERLLKVILAPHISEKSTVNAEKH-NTVVFRVAIDATKAEIKAAVAKLF-EV  53
  sp|Q9KNY6|RL23_VIBCH           -----MIREERLLKVLRAPHISEKATMSAEKS-NTIVFKVAKDATKKEIKAAVEKLF-EV  53
  tr|A0A1L3IIY2|A0A1L3IIY2_PROST -----MIREERLLKVLRAPHVSEKASTAMEKS-NTIVLKVAKDATKAEIKAAVQKLF-EV  53
→ sp|P0ADZ0|RL23_ECOLI           -----MIREERLLKVLRAPHVSEKASTAMEKS-NTIVLKVAKDATKAEIKAAVQKLF-EV  53
  sp|Q8XGM6|RL23_SALTI           -----MIREERLLKVLRAPHVSEKASTAMEKT-NTIVLKVAKDATKAEIKAAVQKLF-EV  53
                                                :     ::              .       *  :   .   :  .

  sp|P47399|RL23_MYCGE           KPLKINTLIRKPVTIRN---GTKYPGFSKLAKLAVITLPKGM-DIAITGEKTTKKETKDQ 106
  sp|Q0P7S6|RL23_CAMJE           TPKSINSLRMDGKIKR----FRGRLGQRNNYKKFYVKLPEGV-SLENTEA----------  93
  sp|P66119|RL23_HELPY           EPLKINSLKQEGKVKR----FRGKLGQRKSFKKFYVKVPEGA-SIAALGA----------  93
  tr|B2RLZ0|B2RLZ0_PORG3         KVEDVNTMRYDGKRKNRYTKSGLIRGKEASFKKAIVTLKKGE-TIDFFSNI---------  97
  tr|E1WPD1|E1WPD1_BACF6         TVVDVNTVKYAGKNKSRYTKAGIINGRTNAFKKAIVTLKEGD-TIDFYSNI---------  96
  tr|Q8A478|Q8A478_BACTN         TVVDVNTVKYAGKNKSRYTKAGIINGRTNAFKKAIVTLKEGD-TIDFYSNI---------  96
  sp|P9WHB9|RL23_MYCTU           KVASVNTANRQGKRKR----TRTGYGKRKSTKRAIVTLAPGSRPIDLFGAPA-------- 100
  sp|A3CK65|RL23_STRSV           KVANVNTINVKPKAKR----VGRYTGFTNKTKKAIVTLTADSKAIELFGAEEE-------  98
  sp|P42924|RL23_BACSU           KVDKVNIMNYKGKSKR----VGRYTGMTSRRRKAIVKLTADSKEIEIFEA----------  95
  sp|Q7A459|RL23_STAAN           KVASVNIMNYKPKKKR----MGRYQGYTNKRRKAIVTLKEGSI--DLFN-----------  91
  tr|Q98PY3|Q98PY3_MYCPU         KVEKVSIINIDKKPKR----VGRFNGFTNSVKKAYVYLAQGN-SINLFPQDPSVEQELKT 103
  sp|Q31L09|RL23_SYNE7           KVVGLSTQLPPRKARR----VGRFAGHRAQVKRAVARLADGD-SITLFPEV--------- 100
  tr|A0A9J9LE58|A0A9J9LE58_RHIWR KVTGVNTIVSKGKTKR----WKGTPYKRSDVKKAIVTLAEGQ-SIDVTEGVR-------- 104
  sp|B8H4D6|RL23_CAUVN           KVTKVNTIVTKGKTKR----FRGIVGRRNDVKKAIVTLAEGQ-SIDITTGL---------  98
  sp|Q8UE20|RL23_AGRFC           KVTAVNTLIRKGKTRR----FRGFAGKLKDVKKAVVTLAEGQ-SIDVSTGL---------  97
  tr|A0A6N3JFX8|A0A6N3JFX8_FRATN KVESVNILNVKGKARR----FGRVEGRTKAWKKAYVKLAEGH-DINFVGAE---------  99
  sp|Q5NHW6|RL23_FRATT           KVESVNILNVKGKARR----FGRVEGGTKAWKKAYVKLAEGH-DINFVGAE---------  99
  sp|Q6F7R4|RL23_ACIAD           DVLKVNTTITKGKSKR----FGKTLGRRSDVKKAYVTLKAGQ-DVEMADLGDTAESAAE- 106
  sp|B7IA37|RL23_ACIB5           EVVKVNTTITKGKTKR----FGRTLGRRSDVKKAYVTLKAGQ-DVEMADLGDTAESAAE- 106
  sp|Q63Q13|RL23_BURPS           EVDSVNVLVQKGKQKR----FGRSMGRRKDVKKAYVCLKPGQ-EINFEAEAK-------- 104
  sp|Q2SU29|RL23_BURTA           EVDSVNVLVQKGKQKR----FGRSMGRRKDVKKAYVCLKPGQ-EINFEAEAK-------- 104
  sp|B4E5C2|RL23_BURCJ           EVDSVNVLVQKGKQKR----FGRSMGRRKDVKKAYVCLKPGQ-EINFEAEAK-------- 104
  sp|Q9HWD7|RL23_PSEAE           KVQRVTTLNVKGKTKR----TARGLGKRNDWKKAYIALQPGQ-DLDFATSAE--------  99
  sp|P44361|RL23_HAEIN           KVDSVRTVVVKGKTKR----RGNKMGRRSDWKKAYVTLAEGQ-NLDFVDSAE--------  99
  sp|Q8EK66|RL23_SHEON           EVESVRTLVSKGKTKR----TGGRTGRRSDWKKAYVTLAAGA-DIDFVGGAE-------- 100
  sp|Q9KNY6|RL23_VIBCH           EVKSVNTLIIKGKTKR----QGLRQGRRSDVKKAYVTLNEGQ-DLDFVGGAE-------- 100
  tr|A0A1L3IIY2|A0A1L3IIY2_PROST EVEGVNTLLVKGKTKR----HGQRFGRRSDWKKAYVTLKEGQ-NLDFIGGAE-------- 100
→ sp|P0ADZ0|RL23_ECOLI           EVEVVNTLVVKGKVKR----HGQRIGRRSDWKKAYVTLKEGQ-NLDFVGGAE-------- 100
  sp|Q8XGM6|RL23_SALTI           EVEVVNTLVVKGKVKR----HGQRIGRRSDWKKAYVTLKEGQ-NLDFVGGAE-------- 100
                                     :                          :     :  .

  sp|P47399|RL23_MYCGE           ---------------------------------------------------          106
  sp|Q0P7S6|RL23_CAMJE           ---------------------------------------------------           93
  sp|P66119|RL23_HELPY           ---------------------------------------------------           93
  tr|B2RLZ0|B2RLZ0_PORG3         ---------------------------------------------------           97
  tr|E1WPD1|E1WPD1_BACF6         ---------------------------------------------------           96
  tr|Q8A478|Q8A478_BACTN         ---------------------------------------------------           96
  sp|P9WHB9|RL23_MYCTU           ---------------------------------------------------          100
  sp|A3CK65|RL23_STRSV           ---------------------------------------------------           98
  sp|P42924|RL23_BACSU           ---------------------------------------------------           95
  sp|Q7A459|RL23_STAAN           ---------------------------------------------------           91
  tr|Q98PY3|Q98PY3_MYCPU         KEVEAESAKTKKASDAEKRAAEKIAAKNIKSTKANTSASTPKIRVRKVADK          154
  sp|Q31L09|RL23_SYNE7           ---------------------------------------------------          100
  tr|A0A9J9LE58|A0A9J9LE58_RHIWR ---------------------------------------------------          104
  sp|B8H4D6|RL23_CAUVN           ---------------------------------------------------           98
  sp|Q8UE20|RL23_AGRFC           ---------------------------------------------------           97
  tr|A0A6N3JFX8|A0A6N3JFX8_FRATN ---------------------------------------------------           99
  sp|Q5NHW6|RL23_FRATT           ---------------------------------------------------           99
  sp|Q6F7R4|RL23_ACIAD           ---------------------------------------------------          106
  sp|B7IA37|RL23_ACIB5           ---------------------------------------------------          106
  sp|Q63Q13|RL23_BURPS           ---------------------------------------------------          104
  sp|Q2SU29|RL23_BURTA           ---------------------------------------------------          104
  sp|B4E5C2|RL23_BURCJ           ---------------------------------------------------          104
  sp|Q9HWD7|RL23_PSEAE           ---------------------------------------------------           99
  sp|P44361|RL23_HAEIN           ---------------------------------------------------           99
  sp|Q8EK66|RL23_SHEON           ---------------------------------------------------          100
  sp|Q9KNY6|RL23_VIBCH           ---------------------------------------------------          100
  tr|A0A1L3IIY2|A0A1L3IIY2_PROST ---------------------------------------------------          100
→ sp|P0ADZ0|RL23_ECOLI           ---------------------------------------------------          100
  sp|Q8XGM6|RL23_SALTI           ---------------------------------------------------          100
```

Supporting Table S5. Output of Clustal-Omega[5] software, documenting the conservation analysis of the L23 ribosomal protein across *E. coli* and a variety of bacteria whose gene encoding for L23 is non-essential for cell viability. The yellow-highlighted columns denote the sites corresponding to the nonpolar RNC-interacting amino acids of *E. coli* L23 identified in this work (Pro14, Val16, Phe51 and Leu93). The *E. coli* amino-acid sequence is labeled with black arrows. See also Figures 5, S11 and the Materials and Methods.

```
  sp|A0QL16|RL23_MYCA1              ----MATVTDPRDIILAPVISEKSYSLLD-DNVYTFVVHPDSNKTQIKIAIEKIF-SVKV   54
  sp|A0R8I2|RL23_BACAH              -------MRDPRDIIKRPVITERSMEMMA-EKKYTFDVDVKSNKTEVKDALEAIF-GVKV   51
  sp|A4VSF6|RL23_STRSY              --------MNLYDVIKKPVITESSMGQLE-AGKYVFEVDTRAHKLLIKQAVEAAFEGVKV   51
  tr|A0A2J9QGG4|A0A2J9QGG4_STRMG    --------MNLYDVIKKPVVTEKSMHALE-EGKYTFEVDTRAHKLLIKQAVEAAFDGVKV   51
  sp|C1CC08|RL23_STRZJ              --------MNLYDVIKKPVITESSMAQLE-AGKYVFEVDTRAHKLLIKQAVEAAFEGVKV   51
  tr|R4Z804|R4Z804_STRAG            --------MNLYDVIKKPVITEKSMVALE-AGKYTFEVDTRAHKLLIKQAVEAAFDGVKV   51
  sp|Q9A1X2|RL23_STRP1              --------MNLYDVIKKPVITEKSMIALE-AGKYTFEVDTRAHKLLIKQAVEAAFDGVKV   51
  sp|Q6N4T7|RL23_RHOPA              ---MKSIDPRHYDVIVAPVVTEKSTMASE-HNKVVFKVQGGATKPQIKEAVEKLF-DVKV   55
  tr|A0A258HC12|A0A258HC12_9CAUL    --MAAQPTAKHYDTILSPIITEKATILSE-QNKVVFRVAGTSTKDEIAAAVESLF-KVNV   56
  sp|Q5F5S9|RL23_NEIG1              ---MGMNQQRLTQVILVPVVSEKSNVLAEKCNQMTFKVLANATKPEIKAAVELLF-GVQV   56
  sp|Q8XV14|RL23_RALN1              MTQVAKNDHRLMQVLLSPVVSEKATLVADKNEQVVFEVARDANKGEVKAAVELLF-KVEV   59
→ sp|P0ADZ0|RL23_ECOLI              ----MIREERLLKVLRAPHVSEKASTAMEKSNTIVLKVAKDATKAEIKAAVQKLF-EVEV   55
                                                . :  * ::* :          .: *   : *  :  *::  *  *:*

  sp|A0QL16|RL23_MYCA1              ASVNTANRQGKRKRTRTGFGKRKSTKRAIVTLAPGSKPIDLFGAPA-----         100
  sp|A0R8I2|RL23_BACAH              EKVNIMNYKPKAKRVGRHAGFTSRRRKAIVKLTADSKEIEIFQGV------          96
  sp|A4VSF6|RL23_STRSY              ANVNTINVKPKTKRVGRYVGRTNKVKKAIITLAADSKAIELFATADAE---          99
  tr|A0A2J9QGG4|A0A2J9QGG4_STRMG    ASVRTVNVKPKQKRVGRYTGFTSKTKKAIITLTADSKTIDLFAAAEAE---          99
  sp|C1CC08|RL23_STRZJ              ANVNTINVKPKAKRVGRYTGFTNKTKKAIITLTADSKAIELFAAEAE----          98
  tr|R4Z804|R4Z804_STRAG            ASVNTVTVKPKAKRVGRYTGFTSKTKKAIITLTADSKAIELFAAEAE----          98
  sp|Q9A1X2|RL23_STRP1              ASVNTVNVKPKAKRVGRYTGFTSKTKKAIITLTADSKAIELFAAEAE----          98
  sp|Q6N4T7|RL23_RHOPA              KSVNTLVRKGKTKAFRGTFGTQSDVKRAVVTLEEGH-RIDVTTGL------          99
  tr|A0A258HC12|A0A258HC12_9CAUL    LKVNTLVQKGKTKRFRGIMGRRVDIKKAIVTLADGQ-SIDVTTGL------         100
  sp|Q5F5S9|RL23_NEIG1              ASVTTVTTKGKTKRFGRILGRRSDVKKAYVSLVDGQ-ELDLEAAAAAADKE         106
  sp|Q8XV14|RL23_RALN1              ESVQILNQKGKQKRFGRFMGRRDHVKKAYVSLKPGQ-EINFEAEAK-----         104
→ sp|P0ADZ0|RL23_ECOLI              EVVNTLVVKGKVKRHGQRIGRRSDWKKAYVTLKEGQ-NLDFVGGAE-----         100
                                      *     : * *      *     ::* :.*  .   ::.
```

Supporting Table S6: Sequence similarity and identity of L29 epitope 1 (MKAKELREKSVEEL) across ribosomal proteins from *E.coli* (K12 strain). As a summary, pairwise local sequence alignment was performed between the L29 epitope-1 peptide sequence and the *E.coli* large- and small-subunit ribosomal proteins via EMBOSS Water (Smith-Waterman algorithm). The table reports the percentages of similar and identical residues relative to the 14-residue epitope sequence, together with the molecular weights of the corresponding ribosomal proteins. This analysis was performed as part of the validation of the anti-L29 antibodies, to assess the likelihood of potential sequence-based-cross-reactivity with other *E.coli* ribosomal proteins. The ribosomal proteins with comparable molecular weights to L29 are shown in green.

r-Proteins	Parent *E. coli* ribosomal subunit	Mol. weight (kDa)	# of similar residues/total # of residues	% Similarity	# of identical residues/total # of residues	% Identity
L1	50S	29.7	6/14	42%	5/14	35%
L2	50S	24.6	7/14	50%	2/14	14%
L3	50S	22.2	4/14	28%	2/14	14%
L4	50S	22.1	11/14	78%	3/14	21%
L5	50S	20.2	4/14	28%	4/14	28%
L6	50S	18.8	7/14	50%	3/14	21%
L7	50S	12.2	7/14	50%	4/14	28%
L9	50S	15.8	7/14	50%	4/14	28%
L10	50S	17.6	6/14	42%	5/14	35%
L11	50S	14.7	7/14	50%	4/14	28%
L13	50S	16.0	6/14	42%	5/14	35%
L14	50S	13.5	6/14	42%	4/14	28%
L15	50S	15.0	3/14	21%	2/14	14%
L16	50S	15.3	4/14	28%	3/14	21%
L17	50S	14.4	10/14	71%	9/14	64%
L18	50S	12.8	8/14	57%	3/14	21%
L19	50S	13.0	6/14	42%	3/14	21%
L20	50S	13.4	6/14	42%	4/14	28%
L21	50S	11.6	5/14	35%	2/14	14%
L22	50S	12.2	7/14	50%	5/14	35%
L23	50S	11.2	5/14	35%	5/14	35%
L24	50S	11.2	6/14	42%	5/14	35%
L25	50S	10.7	6/14	42%	3/14	21%
L27	50S	9.0	5/14	35%	4/14	28%
L28	50S	8.9	5/14	35%	3/14	21%
L29	50S	7.3	14/14	100%	14/14	100%
L30	50S	6.4	3/14	21%	3/14	21%
L31	50S	7.9	5/14	35%	3/14	21%
L32	50S	6.3	3/14	21%	3/14	21%
L33	50S	6.2	6/14	42%	5/14	35%
L34	50S	5.4	4/14	28%	3/14	21%
L35	50S	7.2	7/14	50%	3/14	21%

L36	50S	4.4	5/14	35%	3/14	21%
S1	30S	61.1	9/14	64%	4/14	28%
S2	30S	26.7	6/14	42%	3/14	21%
S3	30S	25.9	7/14	50%	5/14	35%
S4	30S	23.4	5/14	35%	4/14	28%
S5	30S	17.6	11/14	78%	8/14	57%
S6	30S	15.7	7/14	50%	4/14	28%
S7	30S	20.0	7/14	50%	3/14	21%
S8	30S	14.1	6/14	42%	4/14	28%
S9	30S	14.8	6/14	42%	3/14	21%
S10	30S	11.7	7/14	50%	5/14	35%
S11	30S	13.8	5/14	35%	3/14	21%
S12	30S	13.7	5/14	35%	4/14	28%
S13	30S	13.0	8/14	57%	5/14	35%
S14	30S	11.5	10/14	71%	4/14	28%
S15	30S	10.2	4/14	28%	3/14	21%
S16	30S	9.1	3/14	21%	3/14	21%
S17	30S	9.7	4/14	28%	3/14	21%
S18	30S	8.9	5/14	35%	3/14	21%
S19	30S	10.4	5/14	35%	4/14	28%
S20	30S	9.6	5/14	35%	4/14	28%
S21	30S	8.5	7/14	50%	4/14	28%

Supporting Table S7: Sequence similarity and identity of L29 epitope 2 (VARVKTLLNEKAGA) across ribosomal proteins from *E.coli* (K12 strain). As a summary, pairwise local sequence alignment was performed between the L29 epitope-2 peptide sequence and *E.coli* large and small subunit ribosomal proteins via EMBOSS Water (Smith-Waterman algorithm). This table reports the percentages of similar and identical residues relative to the 14-residue epitope sequence, together with the molecular weights of the corresponding ribosomal proteins. This analysis was performed as part of the validation of the anti-L29 antibodies, to assess the likelihood of potential sequence-based-cross-reactivity with other *E.coli* ribosomal proteins. The ribosomal proteins with comparable molecular weights to L29 are shown in green.

r-Proteins	Parent *E. coli* ribosomal subunit	Mol. Wt (kDa)	# of similar residues/total # of residues	% Similarity	# of identical residues/total # of residues	% identity
L1	50S	29.7	4/14	28%	4/14	28%
L2	50S	24.6	4/14	28%	4/14	28%
L3	50S	22.2	7/14	50%	5/14	35%
L4	50S	22.1	7/14	50%	3/14	21%
L5	50S	20.2	5/14	35%	4/14	28%
L6	50S	18.8	4/14	28%	3/14	21%
L7	50S	12.2	5/14	35%	4/14	28%
L9	50S	15.8	10/14	71%	7/14	50%
L10	50S	17.6	6/14	42%	3/14	21%
L11	50S	14.7	5/14	35%	4/14	28%
L13	50S	16.0	3/14	21%	3/14	21%
L14	50S	13.5	9/14	64%	5/14	35%
L15	50S	15.0	9/14	64%	6/14	42%
L16	50S	15.3	3/14	21%	2/14	14%
L17	50S	14.4	4/14	28%	4/14	28%
L18	50S	12.8	8/14	57%	5/14	35%
L19	50S	13.0	6/14	42%	5/14	35%
L20	50S	13.4	7/14	50%	5/14	35%
L21	50S	11.6	7/14	50%	4/14	28%
L22	50S	12.2	6/14	42%	4/14	28%
L23	50S	11.2	5/14	35%	3/14	21%
L24	50S	11.2	6/14	42%	3/14	21%
L25	50S	10.7	6/14	42%	3/14	21%
L27	50S	9.0	6/14	42%	3/14	21%
L28	50S	8.9	5/14	35%	3/14	21%
L29	50S	7.3	14/14	100%	14/14	100%
L30	50S	6.4	3/14	21%	3/14	21%
L31	50S	7.9	5/14	35%	1/14	7%
L32	50S	6.3	2/14	14%	2/14	14%
L33	50S	6.2	5/14	35%	5/14	35%
L34	50S	5.4	4/14	28%	3/14	21%
L35	50S	7.2	8/14	57%	4/14	28%
L36	50S	4.4	4/14	28%	4/14	28%

S1	30S	61.1	8/14	57%	6/14	42%
S2	30S	26.7	5/14	35%	3/14	21%
S3	30S	25.9	9/14	64%	3/14	21%
S4	30S	23.4	7/14	50%	4/14	28%
S5	30S	17.6	8/14	57%	5/14	35%
S6	30S	15.7	5/14	35%	4/14	28%
S7	30S	20.0	8/14	57%	4/14	28%
S8	30S	14.1	8/14	57%	3/14	21%
S9	30S	14.8	7/14	50%	2/14	14%
S10	30S	11.7	4/14	28%	2/14	14%
S11	30S	13.8	4/14	28%	3/14	21%
S12	30S	13.7	4/14	28%	4/14	28%
S13	30S	13.0	7/14	50%	4/14	28%
S14	30S	11.5	7/14	50%	2/14	14%
S15	30S	10.2	8/14	57%	7/14	50%
S16	30S	9.1	5/14	35%	4/14	28%
S17	30S	9.7	5/14	35%	2/14	14%
S18	30S	8.9	6/14	42%	3/14	21%
S19	30S	10.4	3/14	21%	3/14	21%
S20	30S	9.6	8/14	57%	4/14	28%
S21	30S	8.5	4/14	28%	4/14	28%

SUPPORTING FIGURES

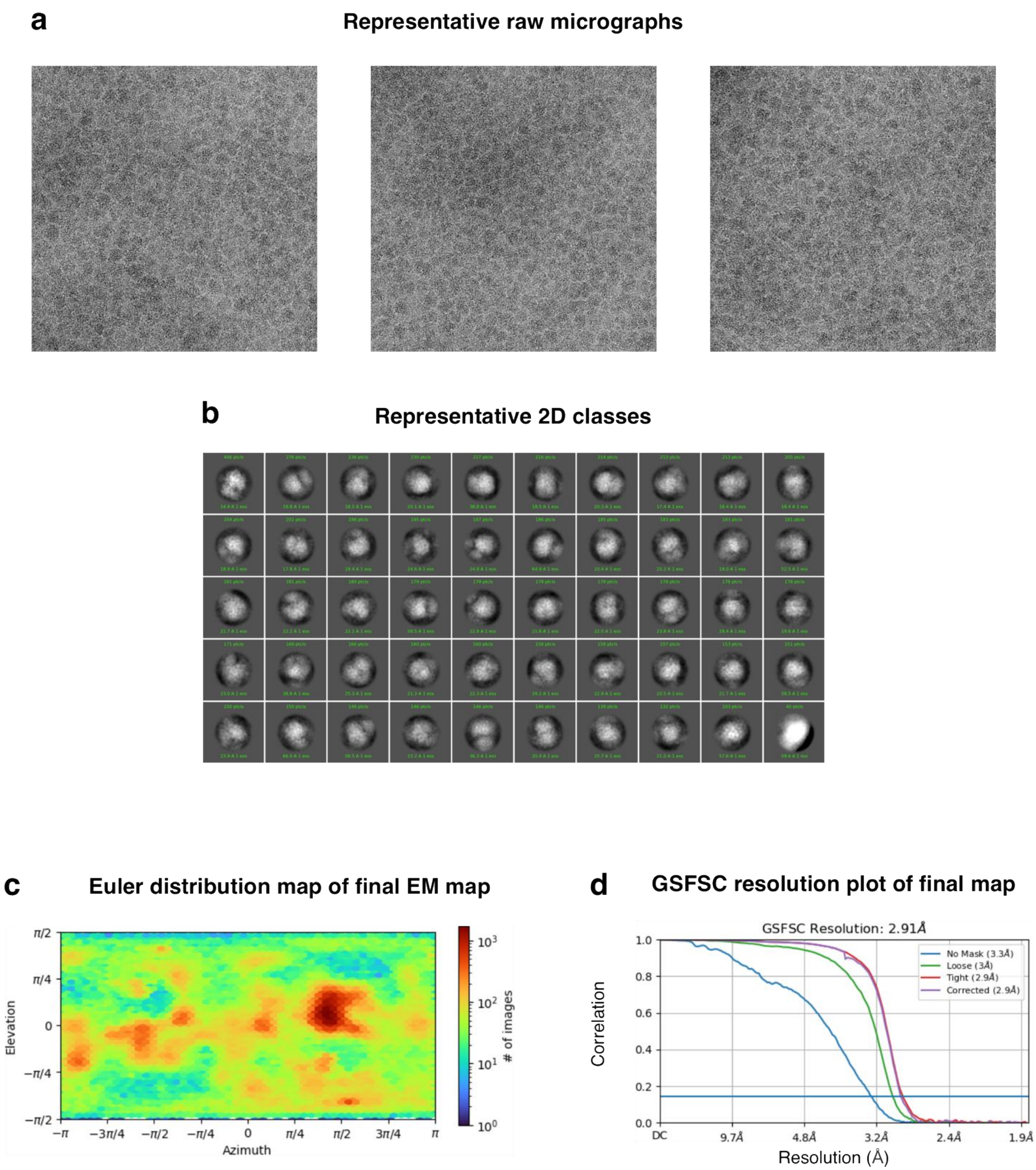

Supporting Figure S1. Sp-cryo-EM data processing information. (a) Representative raw micrographs after gain correction. (b) Representative 2D classes showing multiple projections of

the 70S ribosome. Note that, at this resolution, the nascent chain cannot be visualized. (c) Euler distribution map of the final 3D map showing that the collected particles were not in any preferred orientation, during data collection. (d) Gold Standard Fourier Shell Correlation (GSFSC) at a cutoff value of 0.143, showing that the average resolution of the solved cryo-EM map is 2.91 Å.

Data processing workflow for apoMb RNCs

Movie import into CryoSPARC
(9,112 movies),
motion correction,
CTF estimation

↓

Particle picking and 2D Classification
chose particles that resemble ribosomes
(821,587 particles chosen /1,487,887 particles total)

↓

***Ab initio* reconstruction (3D classification)**
(4 groups with 821,587 particles)

↙ ↘

Cluster 0,2 and 3:
particles that do not
resemble ribosomes
(283,813 particles)

Cluster 1:
particles that resemble
ribosomes
(397,467 particles)

↓

***Ab initio* reconstruction (3D classification)**
to generate low-resolution
cryoEM density maps
(4 groups with 397,467 particles

↙ ↘

Cluster 1 and 2:
particles that do not
resemble ribosomes
(48,946 particles)

Cluster 0 and 3:
particles that resemble
ribosomes
(348,521 particles)

↓

Non-uniform refinement
to generate cryoEM density
maps and masks

↓

3D-variability analysis to tease out
particles that include P-site tRNA

↙ ↘

Particles
lacking p-site tRNA
(134,742 particles)

Particles
including P-site tRNA
(213,770 particles)

↓

3D cryoEM density map generation
(higher resolution)
using non-uniform refinement

↓

Local-resolution estimation

↓

Low-pass filtering (RELION)

↓

3D structural-model building and refinement
to generate final structure
(ChimeraX, Coot)

↓

3D structural-model validation and
assessment of fit quality
(Phenix)

Supporting Figure S2. Cartoon illustrating the sp-cryo-EM data processing workflow. Itemized diagram illustrating the major steps involved in processing the sp-cryo-EM data presented in this study. The pertinent number of particles carried over from step to step is shown in parenthesis. The gold standard Fourier shell correlation (GSFSC) of the 3D map generated from non-uniform refinement is shown in Supplementary Figure S1.

Local resolution maps

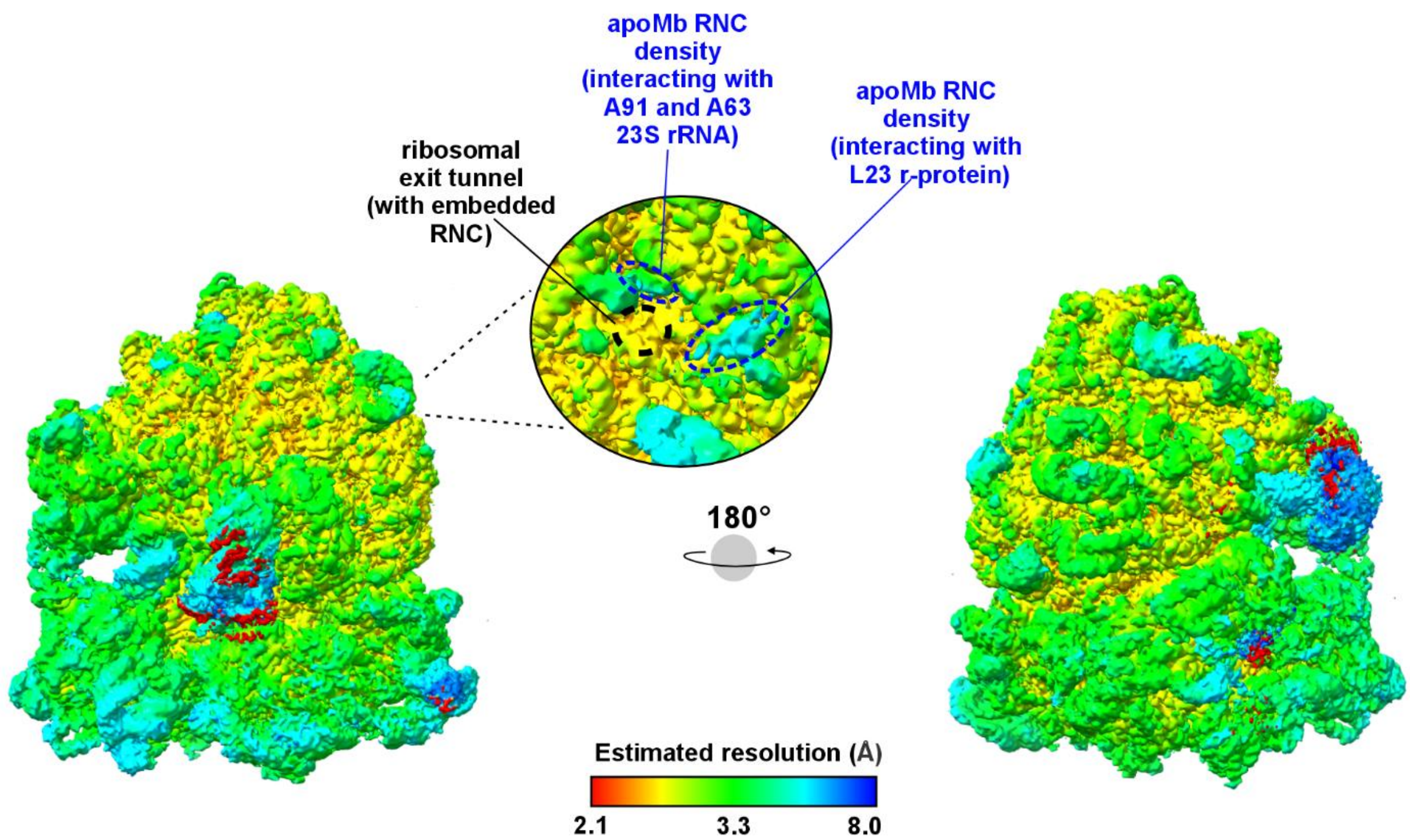

Supporting Figure S3. Sp-cryo-EM local resolution map of apoMb RNCs. The average resolution of the final map was 2.91 Å. Note that a large portion of the density corresponding to the nascent chain (RNC) has a worse resolution than the remainder of the structure. Also note that red-colored density does not correspond to a high-resolution region but is an artifact due to overfitted noise. The presence of this noise is unavoidable due to threshold requirements to adequately visualize the nascent-chain density.

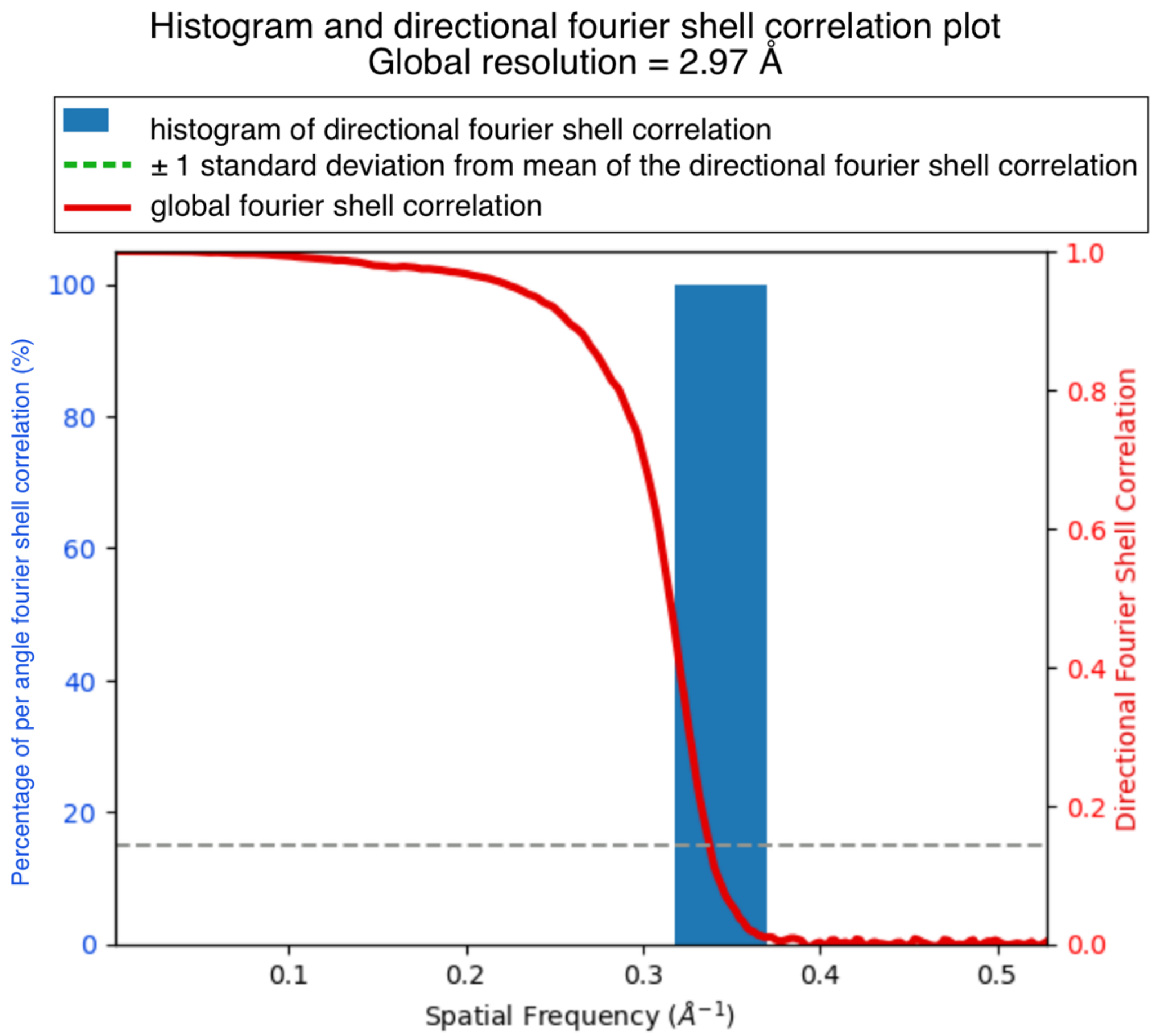

Supporting Figure S4. Histogram and directional FSC plot of final sp-cryo-EM apoMb RNCs. Plot showing the result of the 3D FSC job in CryoSPARC.

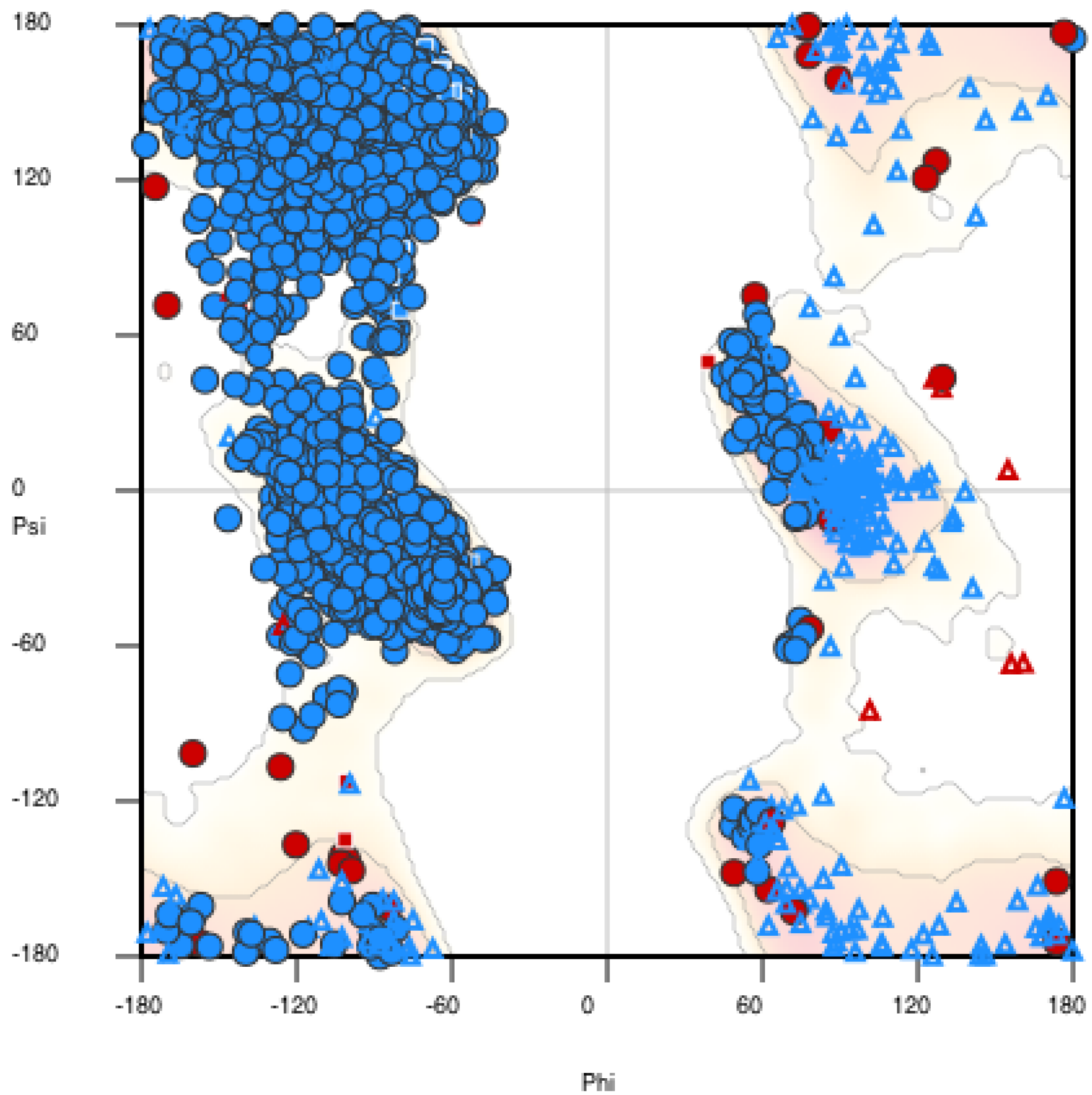

Supporting Figure S5. Ramachandran plot of the final PDB file defining the apoMb RNC/ribosome complex whose structure was determined in this work. Ramachandran plot [6] showing the Phi and Psi backbone dihedral angles of the final apoMb RNC/ribosome structural model. Blue symbols denote residues that lie within Ramachandran-compliant regions [6, 7] of the plot, while red symbols denote outlier residues. Triangles denote glycines, while squares denote prolines.

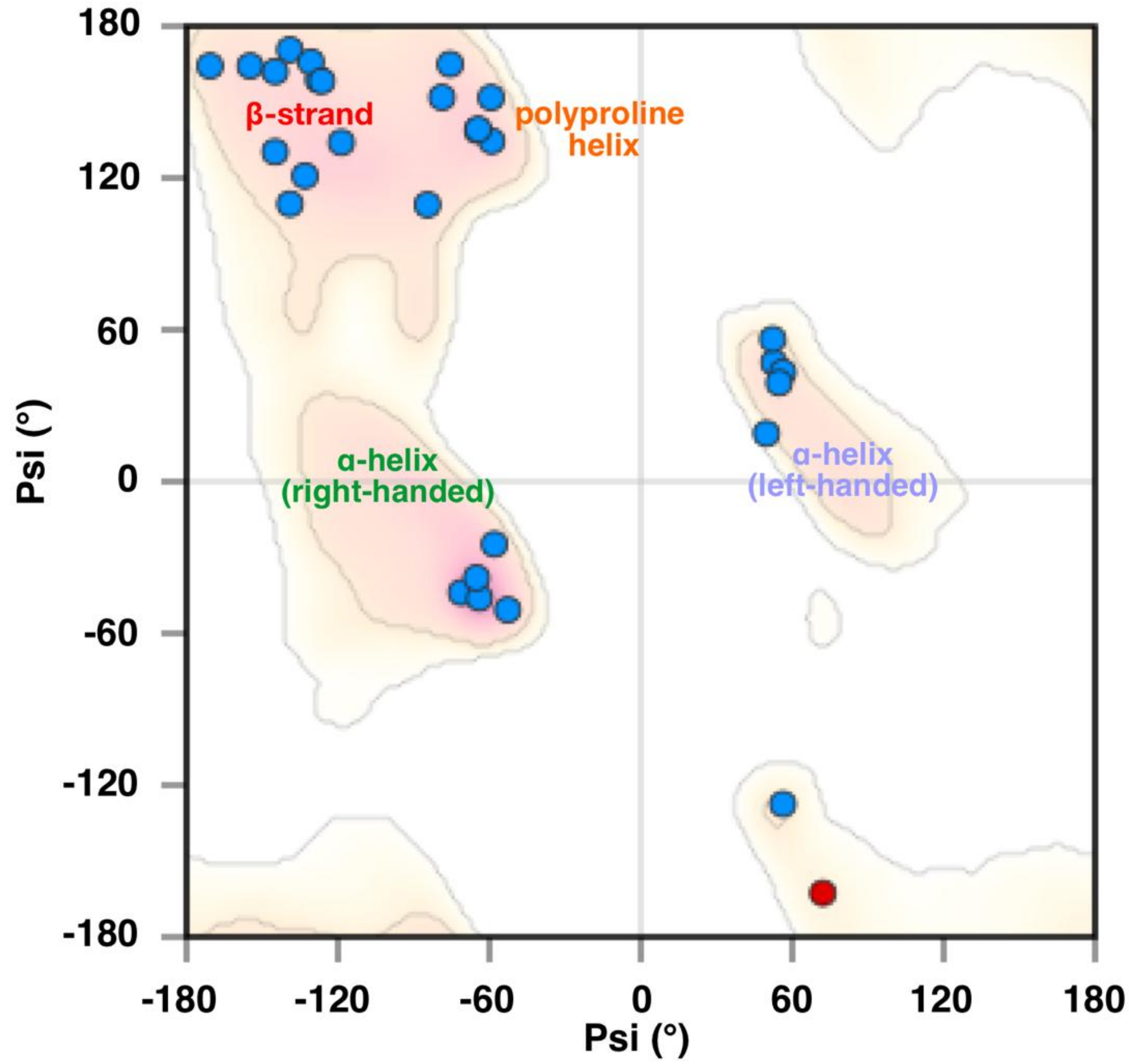

Supporting Figure S6. Ramachandran plot of the final PDB file defining the apoMb ribosome-bound nascent chain (RNC). Ramachandran plot [6] showing exclusively the Phi and Psi backbone dihedral angles of the final apoMb RNC/ribosome structural model (according to a hypothetical poly-Ala chain, as described in the main text). Blue symbols denote residues that lie within Ramachandran-compliant regions [6, 7] of the plot, while red symbols denote outlier residues.

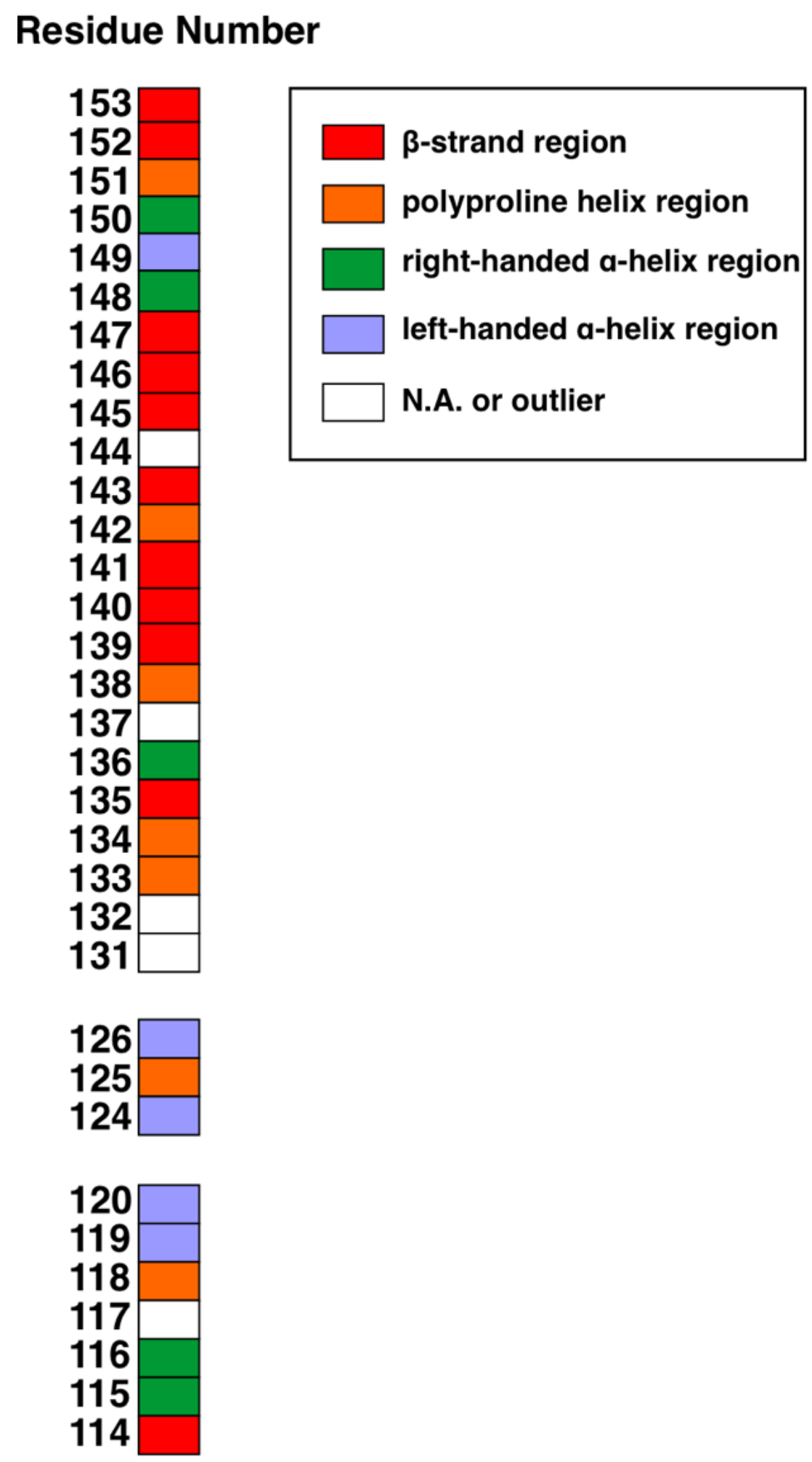

Supporting Figure S7. Map of secondary-structure regions populated by amino acids comprising the apoMb ribosome-bound nascent chain (RNC). Secondary-structure regions are defined according to Ramachandran *et al.* [6], and are deduced from the Ramachandran plot of Supplementary Figure S6. Digits on the lefthand side denote the amino acid number of the apoMb nascent-protein chain (modeled as poly-Ala). The pertinent secondary-structure regions are described in the boxed legend.

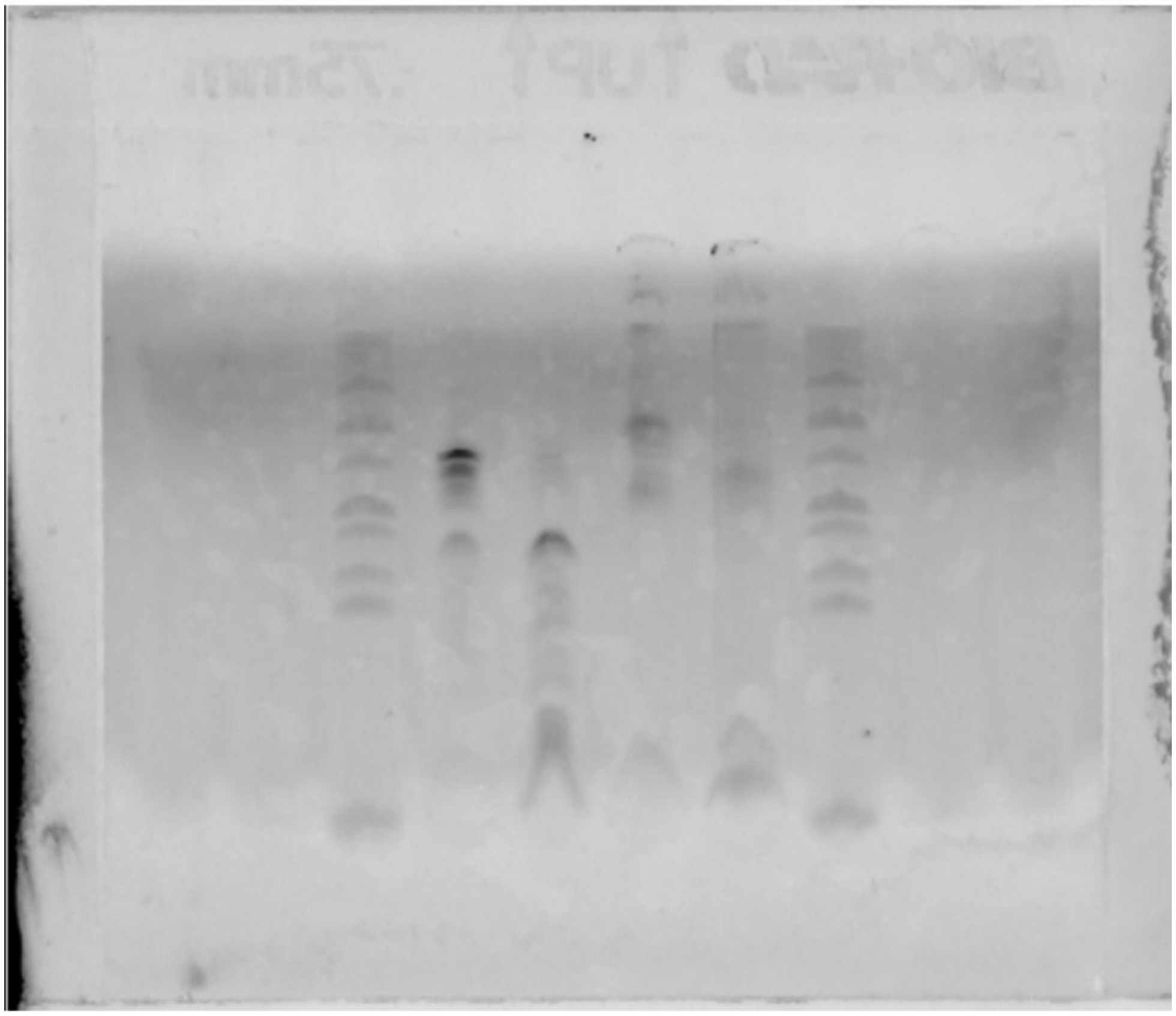

Supporting Figure S8. Uncropped gel images. Uncropped image of the low-pH SDS-PAGE gel shown in Figure 1d.

a

Fluorescence-lifetime data of apoMb ribosome-bound nascent chains (RNCs)

Non-crosslinked	EDC-crosslinked
○ Phase shift<br>◆ Modulation	□ Phase shift<br>▲ Modulation

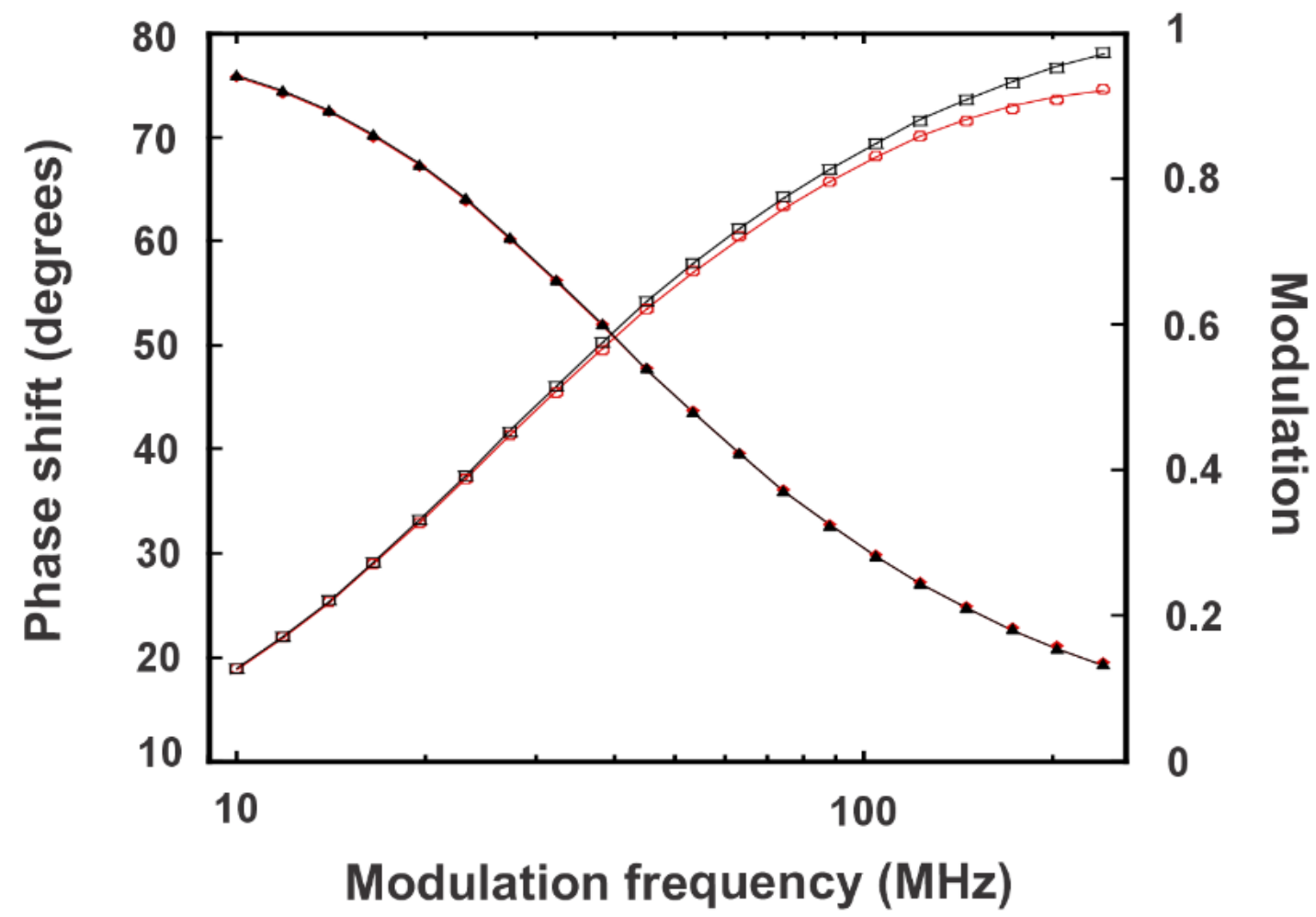

b

Fluorescence-lifetime (L1 and L2) data of apoMb ribosome-bound nascent chains (RNCs)

	Long		Short		Avg reduced χ^2	Avg life-time (ns)
	Fraction	L1 (ns)	Fraction	L2 (ns)		
Non-crosslinked	0.873 ± 0.009	5.98 ± 0.01	0.114 ± 0.007	2.0 ± 0.2	0.53 ± 0.07	5.52 ± 0.01
EDC-crosslinked	0.913 ± 0.003	5.85 ± 0.03	0.079 ± 0.003	1.76 ± 0.06	0.15 ± 0.03	5.53 ± 0.03

Supporting Figure S9. Fluorescence lifetimes of apoMb ribosome-bound nascent chains in the absence and presence of chemical crosslinking. Representative plots illustrating the experimental fluorescence-lifetime profiles of full-length apoMb RNCs in the absence (red) and presence (black) of the EDC chemical crosslinker. Data were collected by frequency-domain

fluorometry. RNC samples were first generated in an *E. coli* cell-free system in the presence of selective N-terminal labeling with the BODIPY-FL fluorophore. The RNCs were centrifuged to generate a soft ribosomal pellet, and then resuspended in buffer (see Materials and Methods), prior to spectroscopic analysis. (b) Table illustrating the fitted fluorescence lifetimes (L1 and L2) and corresponding preexponential fractions of apoMb RNCs in the absence and presence of EDC crosslinking (n=3, avg ±SE). The symbol χ^2 denotes the reduced chi-square of the fits.

a

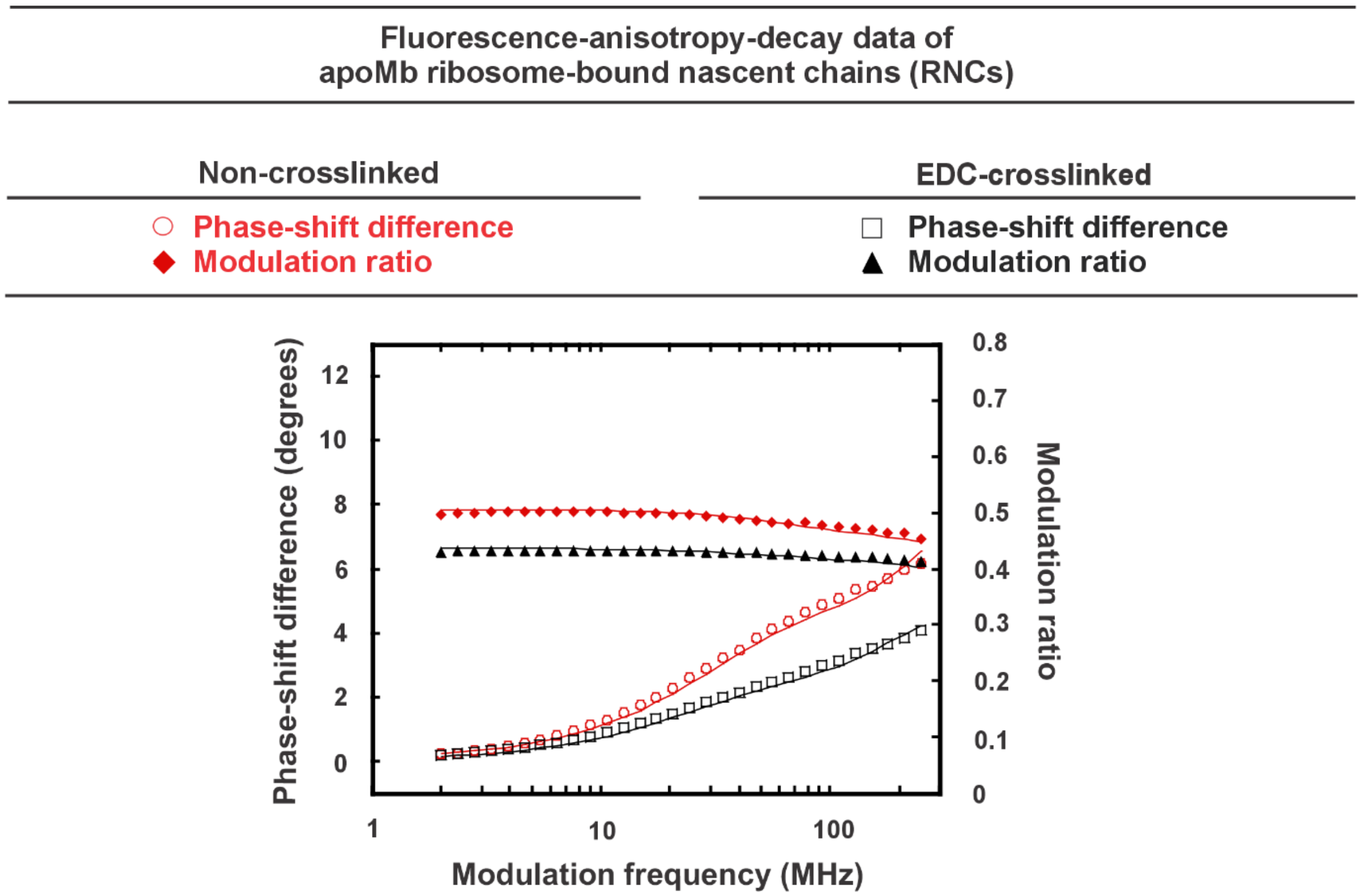

b

Fluorescence-anisotropy-decay parameters of apoMb ribosome-bound nascent chains (RNCs)

	Slow	Intermediate			Fast			Avg reduced χ^2	Predicted # of residues in compact region
	$\tau_{c,s}$ (ns)	$\tau_{c,I}$ (ns)	θ_I (°)	S_I	$\tau_{c,F}$ (ns)	θ_F (°)	S_F		
Non-cross-linked	1,000	4.0 ± 0.5	18 ± 1	0.930 ± 0.008	0.42 ± 0.03	27.6 ± 0.6	0.835 ± 0.006	2.5 ± 0.3	89 ± 10
EDC-cross-linked	1,000	9 ± 1	15.7 ± 0.2	0.945 ± 0.001	0.43 ± 0.03	19.5 ± 0.2	0.916 ± 0.002	1.02 ± 0.06	207 ± 30

Supporting Figure S10. Fluorescence anisotropy decays of apoMb ribosome-bound nascent chains in the absence and presence of chemical crosslinking. Representative plots illustrating the experimental fluorescence anisotropy decays of full-length apoMb RNCs in the absence (red) and presence (black) of the EDC 0chemical crosslinker. Data were collected by frequency-domain fluorometry.[8] RNC samples were first generated in an *E. coli* cell-free system

in the presence of selective N-terminal labeling with the BODIPY-FL fluorophore. The RNCs were centrifuged to generate a soft ribosomal pellet, and then resuspended in buffer (see Materials and Methods), prior to spectroscopic analysis. (b) Table illustrating the fitted fluorescence anisotropy-decay parameters of apoMb RNCs in the absence and presence of EDC crosslinking (n=3, avg ±SE). The symbols $t_{c,S}$, $t_{c,I}$, and $t_{c,F}$ denote rotational correlation times for the slow- (μs timescale), intermediate- (low-ns timescale), and fast- (sub-ns timescale) local motions, respectively. The symbols θ_I and θ_F denote cone semi-angles corresponding to the intermediate- and fast-timescale local motions of the RNCs, respectively. Finally, χ^2 denotes the reduced chi-square of the fits. The predicted number of residues in the compact domain, determined as described,[9] is also shown in the Table.

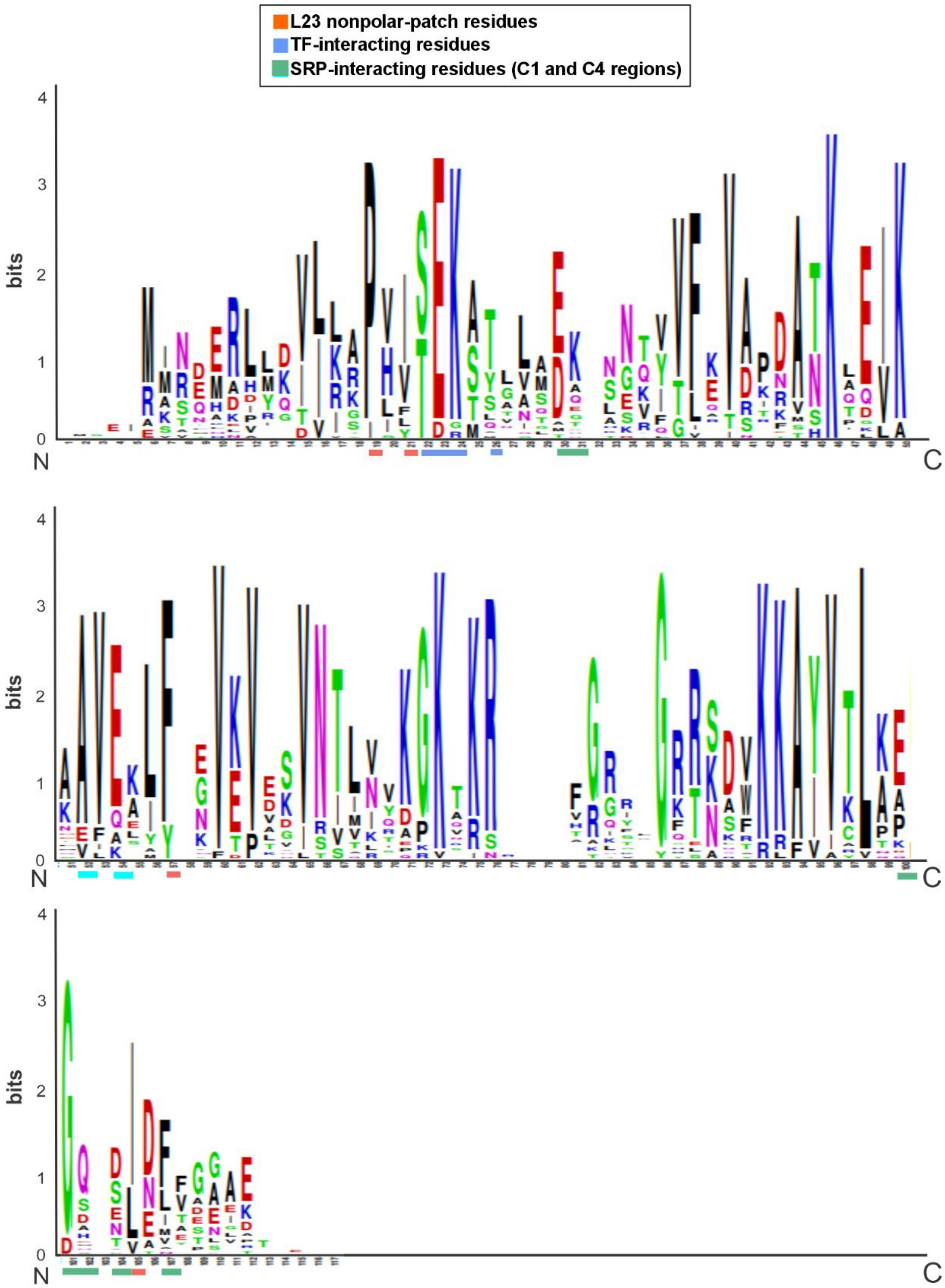

Supporting Figure S11. Conservation analysis of L23 ribosomal protein. Diagram illustrating the amino-acid conservation of the L23 ribosomal protein across bacteria whose L23 gene is essential for cell viability (based on the DEG database, see details in Materials and Methods). Conservation patterns are rendered as a logo plot.

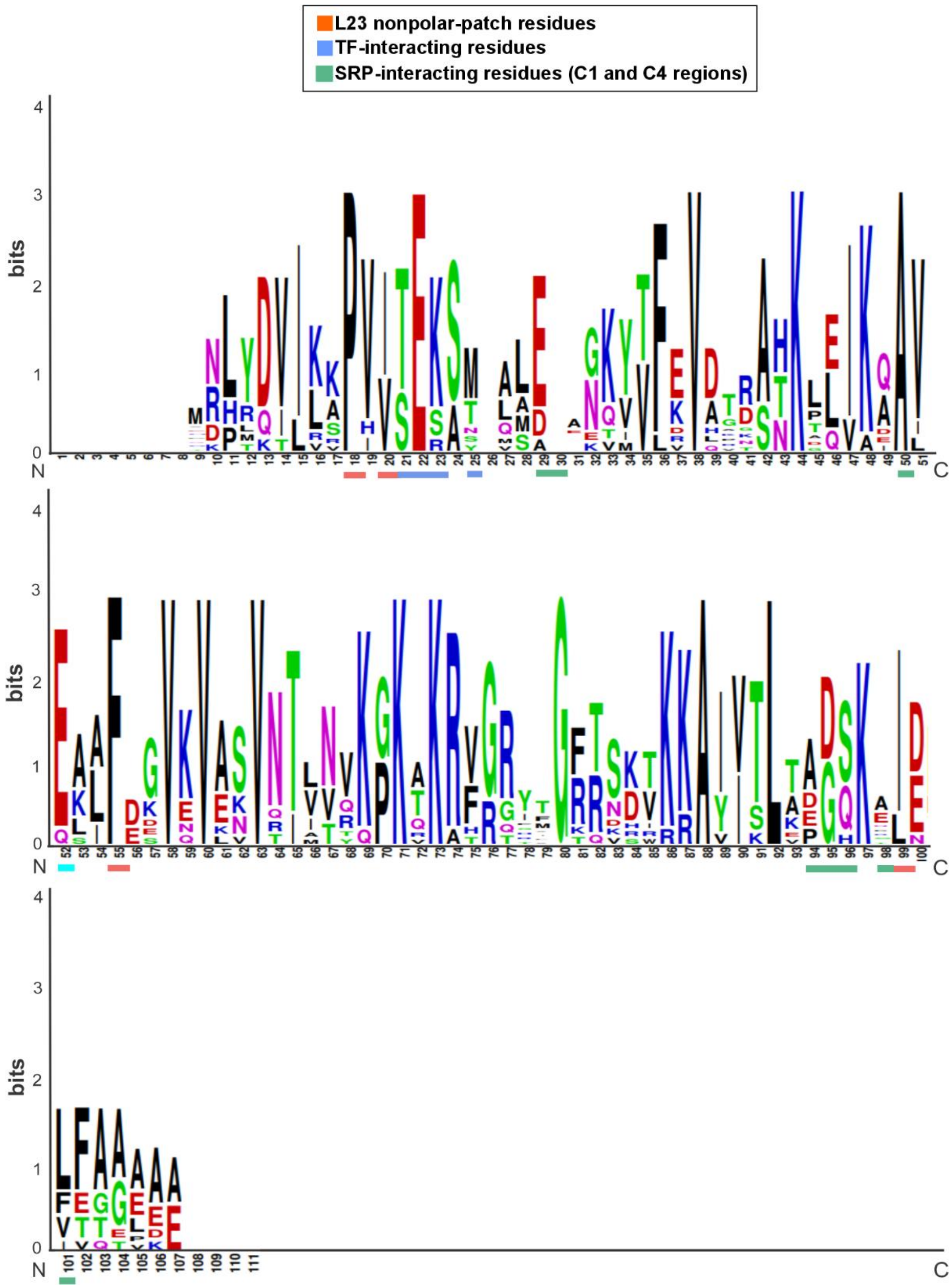

Supporting Figure S12. Additional conservation analysis of L23 ribosomal protein. Diagram illustrating the amino-acid conservation of L23 ribosomal protein across *E. coli* and other bacteria (out of the DEG database) whose L23 gene is non-essential for cell viability (see details in Materials and Methods). Conservation patterns are rendered as a logo plot.

SUPPORTING REFERENCES

(1) Punjani, A.; Rubinstein, J. L.; Fleet, D. J.; Brubaker, M. A. Cryosparc: Algorithms for Rapid Unsupervised Cryo-Em Structure Determination. *Nat. Methods* **2017**, *14* (3), 290-296.

(2) Scheres, S. H. Relion: Implementation of a Bayesian Approach to Cryo-Em Structure Determination. *J. Struct. Biol.* **2012**, *180* (3), 519-530.

(3) Emsley, P.; Cowtan, K. Coot: Model-Building Tools for Molecular Graphics. *Acta Cryst. sect. D: Biol. Cryst.* **2004**, *60* (12), 2126-2132.

(4) Adams, P. D.; Afonine, P. V.; Bunkóczi, G.; Chen, V. B.; Davis, I. W.; Echols, N.; Headd, J. J.; Hung, L.-W.; Kapral, G. J.; Grosse-Kunstleve, R. W. Phenix: A Comprehensive Python-Based System for Macromolecular Structure Solution. *Acta Cryst. Sect. D: Biol. Cryst.* **2010**, *66* (2), 213-221.

(5) Sievers, F.; Wilm, A.; Dineen, D.; Gibson, T. J.; Karplus, K.; Li, W.; Lopez, R.; McWilliam, H.; Remmert, M.; Söding, J. Fast, Scalable Generation of High-Quality Protein Multiple Sequence Alignments Using Clustal Omega. *Mol. Syst. Biol.* **2011**, *7* (1), 539.

(6) Ramachandran, G.; Ramakrishnan, C.; Sasisekharan, V. Stereochemistry of Polypeptide Chain Configurations. *J. Mol. Biol* **1963**, *7*, 95-99.

(7) Lovell, S. C.; Davis, I. W.; Adrendall, W. B.; de Bakker, P. I. W.; Word, J. M.; Prisant, M. G.; Richardson, J. S.; Richardson, D. C. Structure Validation by C Alpha Geometry: Phi,Psi and C Beta Deviation. *Proteins: Struct. Funct. Gen.* **2003**, *50* (3), 437-450.

(8) Jameson, D. M.; Gratton, E.; Hall, R. D. The Measurement and Analysis of Heterogeneous Emissions by Multifrequency Phase and Modulation Fluorometry. *Appl. Spectrosc. Rev.* **1984**, *20* (1), 55-106.

(9) Hutchinson, R. B.; Chen, X.; Zhou, N.; Cavagnero, S. Fluorescence Anisotropy Decays and Microscale-Volume Viscometry Reveal the Compaction of Ribosome-Bound Nascent Proteins. *J. Phys. Chem. B* **2021**, *125* (24), 6543-6558.
